# Novel Steroidal[17,16-*d*]pyrimidines Derived from Epiandrosterone and Androsterone: Synthesis, Characterization and Configuration-Activity Relationships

**DOI:** 10.3390/molecules28062691

**Published:** 2023-03-16

**Authors:** Fei Yang, Fang Liu, Yong Min, Liqiao Shi, Manli Liu, Kaimei Wang, Shaoyong Ke, Yan Gong, Ziwen Yang

**Affiliations:** 1 College of Life Sciences, Wuhan University, Wuhan 430072, China; 2 Key Laboratory of Microbial Pesticides, Ministry of Agriculture and Rural Affairs, National Biopesticide Engineering Research Centre, Hubei Biopesticide Engineering Research Centre, Hubei Academy of Agricultural Sciences, Wuhan 430064, China

**Keywords:** epiandrosterone, androsterone, steroidal[17,16-*d*]pyrimidines, synthesis, bioactivity, molecular docking, SARs

## Abstract

Two series of novel steroidal[17,16-*d*]pyrimidines derived from natural epiandrosterone and androsterone were designed and synthesized, and these compounds were screened for their potential anticancer activities. The preliminary bioassay indicated that some of these prepared compounds exhibited significantly good cytotoxic activities against human gastric cancer (SGC-7901), lung cancer (A549), and hepatocellular liver carcinoma (HepG2) cell lines compared with 5-fluorouracil (5-FU), epiandrosterone, and androsterone. Especially the respective pairs from epiandrosterone and androsterone showed significantly different inhibitory activities, and the possible configuration-activity relationships have also been summarized and discussed based on kinase assay and molecular docking, which indicated that the inhibition activities of these steroidal[17,16-*d*]pyrimidines might obviously be affected by the configuration of the hydroxyl group in the part of the steroidal scaffold.

## 1. Introduction

Cancer is a disorder that rigorously affects the human population worldwide despite significant improvements in new treatment options [1,2], which is the second leading cause of death in the world. The increasing multidrug resistance and side effects have become an important cause of clinical death in the twenty-first century. Although there are many effective therapies for cancer control, chemotherapy remains the main option to treat cancer disease.

**Figure 1:**
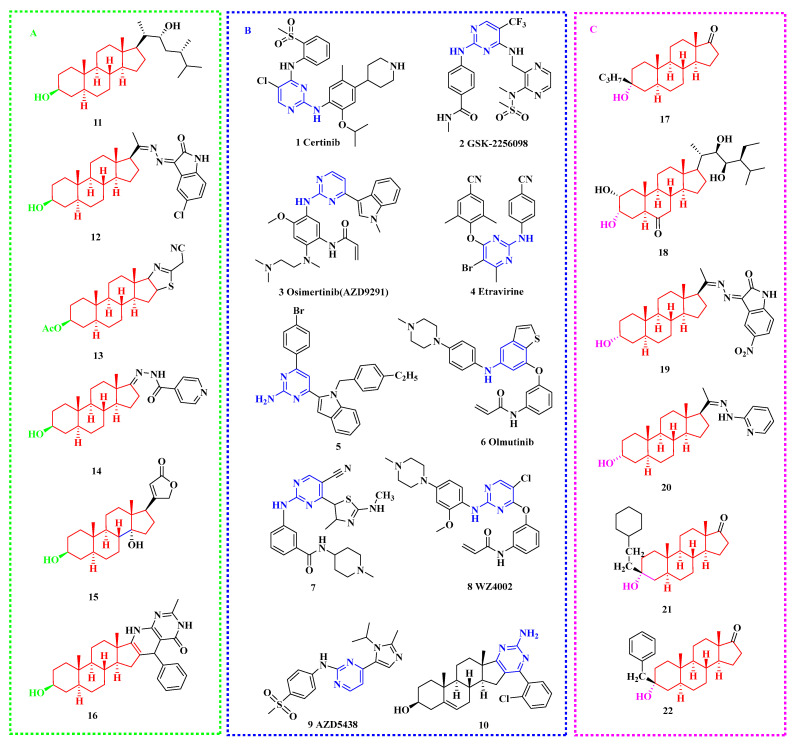
Chemical structures of typical compounds with antiproliferative activity (**A**) epiandrosterone derivatives; (**B**) 2-aminopyrimidine derivatives; (**C**) androsterone derivatives.

It is well known that pyrimidine is a class of aromatic heterocyclic compounds that contain two nitrogen atoms at positions 1 and 3 of the six-membered ring. Heterocyclic compounds bearing the pyrimidine core are of tremendous interest because the pyrimidine structure, which is an important part of many endogenous substances, can easily interact with enzymes, genetic materials, and bio-components within the cell [3,4]. Up to now, many natural and synthetic pyrimidines derivatives have been of enormous importance and demonstrate a variety of pharmacological activities, including anticancer [5,6,7,8,9,10,11], antiviral [12,13,14,15], antifungal [16,17,18], antioxidant [19,20,21,22,23], antibacterial [24,25,26], antituberculosis [27,28], anticonvulsant [29,30,31,32,33,34], antimalarial [35,36,37,38,39,40], antihypertensive [41,42,43,44,45], and anti-inflammatory [46,47,48,49,50,51]. Pyrimidine scaffolds are privileged heterocycles in drug discovery because they have considerable pharmacological and chemical significance and are also easily soluble in water [41]. Moreover, many disubstituted pyrimidines are also used as successful moieties to construct novel functional molecules [4]. Many scientists have focused on the discovery and structural optimization of pyrimidine derivatives, and so many novel pyrimidine derivatives shown in Figure 1 have been discovered [2,3,52,53,54,55,56]. Pyrimidine derivatives have gained a great deal of attention, especially for their considerable antiproliferative activity [2,57,58,59].

In addition, natural steroids from animal and plant metabolites are types of important secondary metabolites that are widely distributed in marine environments and are extremely important biomarkers in the field of marine chemistry. In the marine sedimentary environment, the growth and reproduction of in-situ plankton and the input of terrestrial higher plant debris are the main sources of steroids. In the past few years, many steroids have been used as prototype scaffolds for constructing diverse molecules with extensive pharmaceutical activities, especially for dehydroepiandrosterone (DHEA), epiandrosterone (EPIA), and androsterone (AND) steroids [60,61,62]. Some of these novel steroidal derivatives showed significant inhibitory activities on human tumor cells in culture, suggesting that the natural four fused rings scaffold of steroids might be closely bound up with the cytotoxic activity [63,64,65,66,67]. Some of the EPIA and AND derivatives [67,68,69,70,71,72,73,74,75] that exhibited significant inhibition on cancer cell lines are shown in Figure 1A and Figure 1C, respectively.

**Figure 2:**
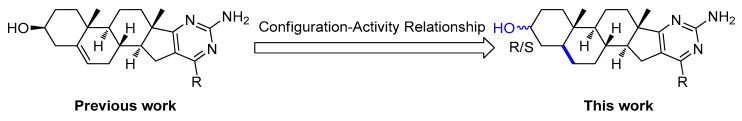
Design strategy of steroidal[17,16-*d*]pyrimidines.

Based on these observations, two series of steroidal[17,16-*d*]pyrimidines derived from epiandrosterone and androsterone were also designed and synthesized, which integrate the structural features of pyrimidines and EPIA/AND unit to a core molecule as indicated in Figure 2, and their cytotoxic effects on tumor cell lines (HepG2, A549, SGC-7901) were fully investigated by MTT colorimetric method, and the possible configuration-activity relationships have also been summarized and discussed based on the activity data, kinase assay, and molecular docking. These results may provide much useful information for the discovery of novel cytotoxic agents from natural steroids.

## 2. Results and Discussion

### 2.1. Chemistry

**Scheme 1:**
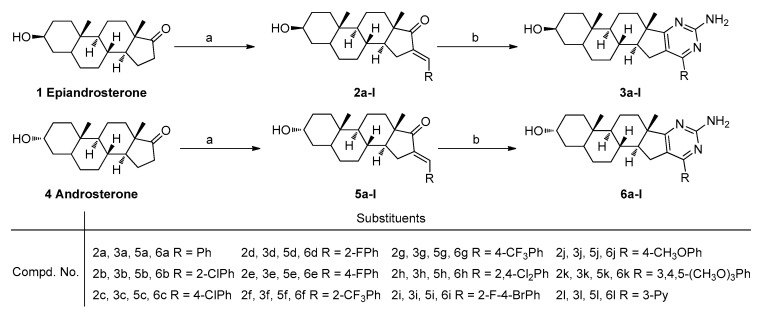
Synthetic route for steroidal[17,16-*d*]pyrimidine derivatives. Reagents and conditions: a. RCHO, NaOH, MeOH, rt, yield 83–94%; b. Guanidine nitrate, ^t^BuOK, ^t^BuOH, reflux, yield 61–91%.

In this work, two series of heterocyclic steroidal[17,16-*d*]pyrimidines derived from epiandrosterone and androsterone were conveniently prepared, and the general method for the preparation of these steroidal[17,16-*d*]pyrimidines derivatives **3a**–**l** and **6a**–**l** is described in Scheme 1.

According to Scheme 1, these steroidal[17,16-*d*]pyrimidines derivatives **3a**–**l** and **6a**–**l** have been conveniently synthesized via a two-step transformation from epiandrosterone or androsterone, respectively. First, the various substituted aromatic aldehydes were treated with epiandrosterone or androsterone via aldol condensation to obtain the intermediates **2a**–**l** and **5a**–**l**. Subsequently, the reaction of intermediates **2a**–**l** and **5a**–**l** with guanidine nitrate in the presence of potassium tert-butoxide have been conveniently processed to obtain the target molecules.

All the newly synthesized steroidal[17,16-*d*]pyrimidines **3a**–**l** and **6a**–**l** gave satisfactory chemical analyses, including ^1^H NMR, ^13^C NMR, and ESI-MS spectra analyses, and the chemical structures and physiochemical properties of these compounds were summarized in the experimental (Some of them have been collected in the Supplementary Materials). For NMR analyses, the assignments of different signals are based on the chemical shifts and intensity patterns. All ^1^H NMR spectra of molecules **3a**–**l** and **6a**–**l** presented distinctive signals of methine proton attached to the hydroxyl group, which always indicated a multiplet or broad singlet at about 3.29–3.55 ppm and 3.77–3.82 ppm, respectively. The signal for protons of the hydroxyl group showed doublet peaks at about 4.43–4.48 ppm in compounds **3a**–**l**, but at 4.16–4.19 ppm in compounds **6a**–**l**. The signal for protons of the 2-amino group attached to the pyrimidine ring resonated almost the same as a singlet between 5.36 ppm and 6.58 ppm. The other set of signals that appeared in their ^1^H NMR spectra in the ranges 2.79–0.69 ppm belonged to the protons of epiandrosterone or androsterone scaffold, and the signals at lower fields were assigned to the signals of aromatic protons as described in general structures in Scheme 1. The ^13^C NMR analysis of molecules **3a**–**l** and **6a**–**l** display obvious peaks in the alkyl region, indicating the presence of the epiandrosterone or androsterone scaffold, respectively. Other peaks appearing at lower fields were assigned to the carbon signals of the aromatic and heterocyclic moieties. The electron spray impact mass spectra (ESI-MS) for compounds **2a**–**l** and **6a**–**l** were measured on a WATERS ACQUITY UPLC^®^ H-CLASS PDA (Waters^®^) instrument, and the ESI-MS of target molecules exhibited obvious molecular peak [M + H]^+^ in the positive ion mode. All the characteristic peaks observed within the ^1^H NMR and ^13^C NMR spectra for title compounds are given in the experimental section.

### 2.2. Pharmacology Evaluation

#### 2.2.1. Inhibitory Effects of the Target Compounds

**Figure 3:**
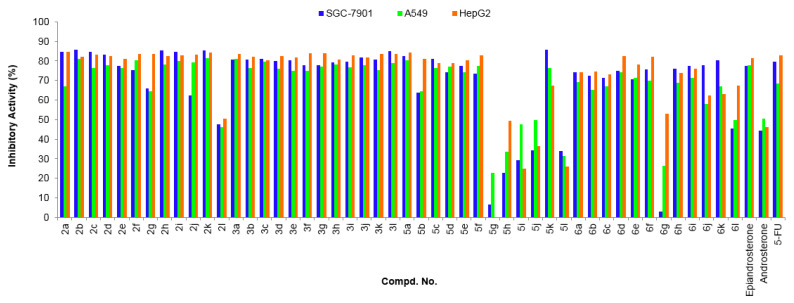
Antitumor activities of compounds **2a**–**l**, **3a**–**l**, **5a**–**l**, and **6a**–**l** at 40 µg/mL. Abbreviations: SGC-7901—Human gastric cancer cell line, A549—Human lung adenocarcinoma cell line; HepG2—human hepatocellular liver carcinoma cell line; 5-FU—5-Fluorouracil, used as a positive control.

All newly synthesized steroidal[17,16-*d*]pyrimidines derivatives **3a**–**l**, **6a**–**l** and the intermediates **2a**–**l**, **5a**–**l** were evaluated for their potential in vitro cytotoxic effects on human gastric cancer (SGC-7901), lung cancer (A549), and hepatocellular liver carcinoma (HepG2) cell lines by the standard MTT (3-(4,5-dimethylthiazol-2-yl)-2,5-diphenyl tetrazolium bromide) assay [76] using 5-FU (5-Fluorouracil) as a positive control. The preliminary screening results are summarized in the following Figure 3. Generally, as shown in Figure 3, we can find most of these steroidal derivatives displayed well in vitro cytotoxic activities against three human cancer cell lines except compounds **2l**, **5g**, **5h**, **5i**, **5j**, **5l**, **6g**, and **6l**. Notably, the compounds **3a**–**l** exhibited obviously inhibitory activities against all tested cell lines with 75.0–84.1% growth inhibition at the concentration of 40 µg/mL. From Figure 3, we also can observe that the steroidal[17,16-*d*]pyrimidines (**3a**–**l**) derived from epiandrosterone presented significantly better inhibitory activities than that of steroidal[17,16-*d*]pyrimidines (**6a**–**l**) derived from androsterone.

The preliminary assay demonstrated that many of these novel steroidal derivatives displayed good inhibitory activities (Figure 3), so in order to further clarify the potential activities, the IC_50_ values for all molecules were fully evaluated. The cytotoxic activities expressed as IC_50_ values for all molecules are described in Table 1, which further confirmed that all the steroidal[17,16-*d*]pyrimidine derivatives **3a**–**l** exhibited higher inhibition activity than that of compounds **6a**–**l** and the commercial 5-FU under the same conditions, respectively.

As indicated in Table 1, compounds **2b**, **2d**, **2h**, **2i**, **2k**, **3a**–**l**, **5c**–**e**, and **6b** have higher cytotoxicity activities (Entries 2, 4, 8, 9, 13–24, 27–29, and 38) against all tested cell lines. In particular, the compounds **3a**, **3b**, **3d**, and **3k** derived from epiandrosterone exhibited significant inhibition (Entries 13, 14, 16, and 23) on all cancer cell lines compared to the positive control 5-FU, the activities of which were also more successful than those of the corresponding **6a**, **6b**, **6d**, and **6k** derived from androsterone. On the whole, the activities of molecules **3a**–**l** are superior to that of molecules **6a**–**l**, which confirms that the configuration of the molecule might have a significant effect on the inhibition and that the compounds with epiandrosterone scaffold are favorable for inhibition activities.

**Figure 4:**
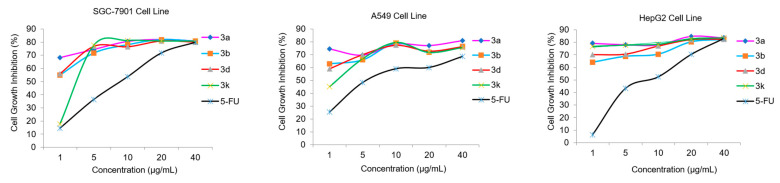
Dose-response analysis of cell growth inhibition activity for the potential compounds **3a**, **3b**, **3d**, **3k**, and **5-FU** (positive control) against SGC-7901, A549, and HepG2 cell lines.

Moreover, the dose-response analysis of cell growth inhibition activities for high potential compounds **3a**, **3b**, **3d**, **3k**, and 5-FU have been displayed in Figure 4, which indicated that the target compounds significantly inhibited SGC-7901, A549, and HepG2 cell proliferation in a concentration-dependent manner. In particular, compound **3a** containing a phenyl unit exhibited the highest potential inhibitory activities against all tested cell lines with the IC_50_ values of 1.07 ± 0.22, 0.61 ± 0.19, and 0.51 ± 0.13 µg·mL^−1^ (Entry 13 in Table 1), respectively, which was significantly better than that of the control 5-FU and epiandrosterone.

#### 2.2.2. Selectivity Profiling of Compound **3a**

Compound **3a** was selected as a representative to further investigate the kinase selectivity profile against a panel of tyrosine kinases (Table 2). The kinase inhibitory activities of compound **3a** (at the concentration of 10 μM) were determined by ADP-Glo Protocol or LANCE Protocol. As shown in Table 2, compounds **3a** displayed some inhibitory effect on the kinases of CDK1/CyclinA2, ALK, FGFR1, and FAK with inhibition rates of 22.51%, 17.36%, 11.82%, and 10.52%, respectively.

#### 2.2.3. Structure and Activity Relationships (SARs) and Molecular Docking

**Figure 5:**
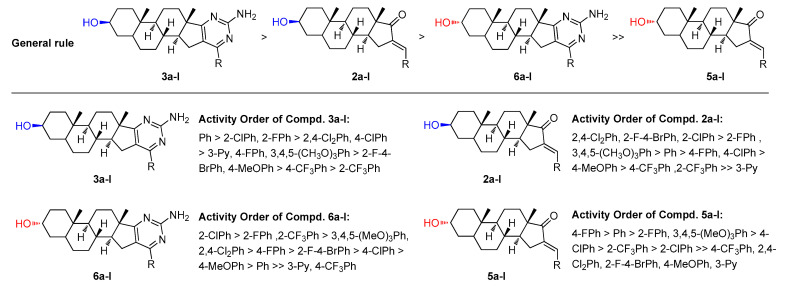
General structure-activity profile for the steroidal derivatives used in this study.

The structure evolution here was to modify epiandrosterone or androsterone scaffold with a pyrimidine ring system (**3a**–**1**, **6a**–**l**) and aromatic enones (**2a**–**l**, **5a**–**l**), respectively. According to the aforementioned results indicated in Table 1, we can obtain the general structure-activity profile for these novel steroidal[17,16-*d*]pyrimidine derivatives (Figure 5).

Generally, as indicated in Figure 5, the compounds containing pyrimidine ring systems exhibit better inhibitory activity than the compounds modified by aromatic enones, which testifies to the potential importance of a pyrimidine core. We also can find that compounds **3a**–**l** exhibited higher inhibition activity than the derivatives **6a**–**l**. In addition, for the compounds containing a pyrimidine ring system, the compounds bearing 2-ClPh, 2-FPh, 2-CF_3_Ph, 2,4-Cl_2_Ph, 4-ClPh, 4-FPh, 3,4,5-(MeO)_3_Ph and 2-F-4-BrPh group present the higher potential activities. However, when the substituents R are Ph and 4-CH_3_OPh, there is a significant difference in efficacy between heterocyclic steroidal[17,16-*d*]pyrimidines derived from epiandrosterone and androsterone. With substituents R are 3-Py and 4-CF_3_Ph especially, the compounds derived from androsterone obviously decreased the cytotoxic activity. On the other hand, for the compounds containing aromatic enone moiety, the results showed that compounds containing 2-FPh, Ph, 4-FPh, 4-ClPh, and 3,4,5-(MeO)_3_Ph group exhibited higher activity than the compounds bearing other substituents. However, the two series of substituted benzylidene derived from epiandrosterone and androsterone showed a significant difference in efficacy when the substituents R are 4-CF_3_Ph, 2,4-Cl_2_Ph, 2-F-4-BrPh, 4-CH_3_OPh and 3-Py. From Table 1, we also can find that, within the series of halogen derivatives, it is clear that the ortho-substituted compounds **2b**, **2d**, **3b**, **3d**, **6b**, and **6d** perform better than the para-substituted compounds **2c**, **2e**, **3c**, **3e**, **6c**, **6e**, respectively. In particular, most of the compounds containing 4-ClPh, 2-FPh, 4-FPh, and 3,4,5-(MeO)_3_Ph group (**2c**, **2d**, **2e**, **2k**, **3c**, **3d**, **3e**, **3k**, **5c**, **5d**, **5e**, **5k**, **6c**, **6d**, **6e** and **6k**) present good inhibitory activities in these four systems, which may be due to the steric size of substituted groups are favourable for the binding to the potential receptor. Compared with steroidal[17,16-*d*]pyrimidines derived from dehydroepiandrosterone, we also can find that the double bond between C_5_ and C_6_ in the B ring decreases the cytotoxic activity [65].

**Figure 6:**
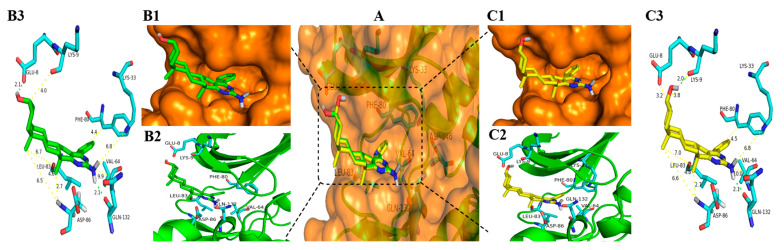
Docking modes of **3a** and **6a** with CDK1 (PDB code: 6GU6). (**A**) Overview of the binding site of **3a** (green stick) and **6a** (yellow stick) with CDK1 (orange transparent surface structure fused with a sticks structure of white hydrogen atoms, cyan carbon atoms, blue nitrogen atoms, and red oxygen atoms); (**B1**) Position of **3a** in the orange surface structure of CDK1; (**B2**) Position of **3a** in cartoon structure of CDK1; (**B3**) Binding model for **3a** bond to CDK1 (Hydrogen bonds are drawn as green dashed lines with distances labelled in Å while the yellow dashed lines just display the distance between the atoms); (**C1**) Position of **6a** in the orange surface structure of CDK1; (**C2**) Position of **6a** in cartoon structure of CDK1; (**C3**) Binding model for **6a** bond to CDK1 (Hydrogen bonds are drawn as green dashed lines with distances labelled in Å while the yellow dashed lines just display the distance between the atoms). Figures were drawn with Pymol (Schrödinger).

**Figure 7:**
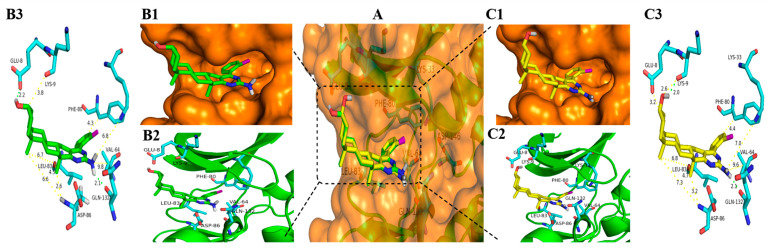
Docking modes of **3b** and **6b** with CDK1 (PDB code: 6GU6). (**A**) Overview of the binding site of **3b** (green stick) and **6b** (yellow stick) with CDK1 (orange transparent surface structure fused with a sticks structure of white hydrogen atoms, cyan carbon atoms, blue nitrogen atoms, red oxygen atoms, and purple chlorine atoms); (**B1**) Position of **3b** in the orange surface structure of CDK1; (**B2**) Position of **3b** in cartoon structure of CDK1; (**B3**) Binding model for the **3b** bond to CDK1 (Hydrogen bonds are drawn as green dashed lines with distances labelled in Å while the yellow dashed lines just display the distance between the atoms); (**C1**) Position of **6b** in the orange surface structure of CDK1; (**C2**) Position of **6b** in cartoon structure of CDK1; (**C3**) Binding model for the **6b** bond to CDK1 (Hydrogen bonds are drawn as green dashed lines with distances labelled in Å while the yellow dashed lines just display the distance between the atoms). Figures were drawn with Pymol (Schrödinger).

**Figure 8:**
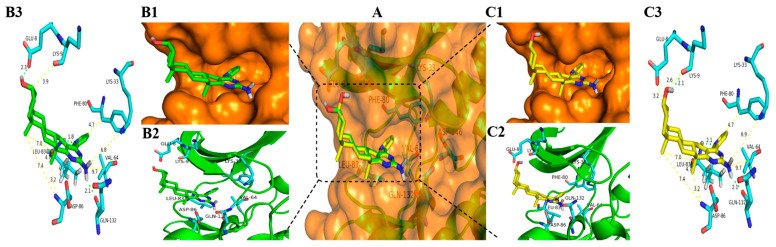
Docking modes of **3l** and **6l** with CDK1 (PDB code: 6GU6). (**A**) Overview of the binding site of **3l** (green stick) and **6l** (yellow stick) with CDK1 (orange transparent surface structure fused with a sticks structure of white hydrogen atoms, cyan carbon atoms, blue nitrogen atoms, and red oxygen atoms); (**B1**) Position of **3l** in the orange surface structure of CDK1; (**B2**) Position of **3l** in cartoon structure of CDK1; (**B3**) Binding model for **3l** bond to CDK1 (Hydrogen bonds are drawn as green dashed lines with distances labelled in Å while the yellow dashed lines just display the distance between the atoms); (**C1**) Position of **6l** in the orange surface structure of CDK1; (**C2**) Position of **6l** in cartoon structure of CDK1; (**C3**) Binding model for **6l** bond to CDK1 (Hydrogen bonds are drawn as green dashed lines with distances labelled in Å while the yellow dashed lines just display the distance between the atoms). Figures were drawn with Pymol (Schrödinger).

**Figure 9:**
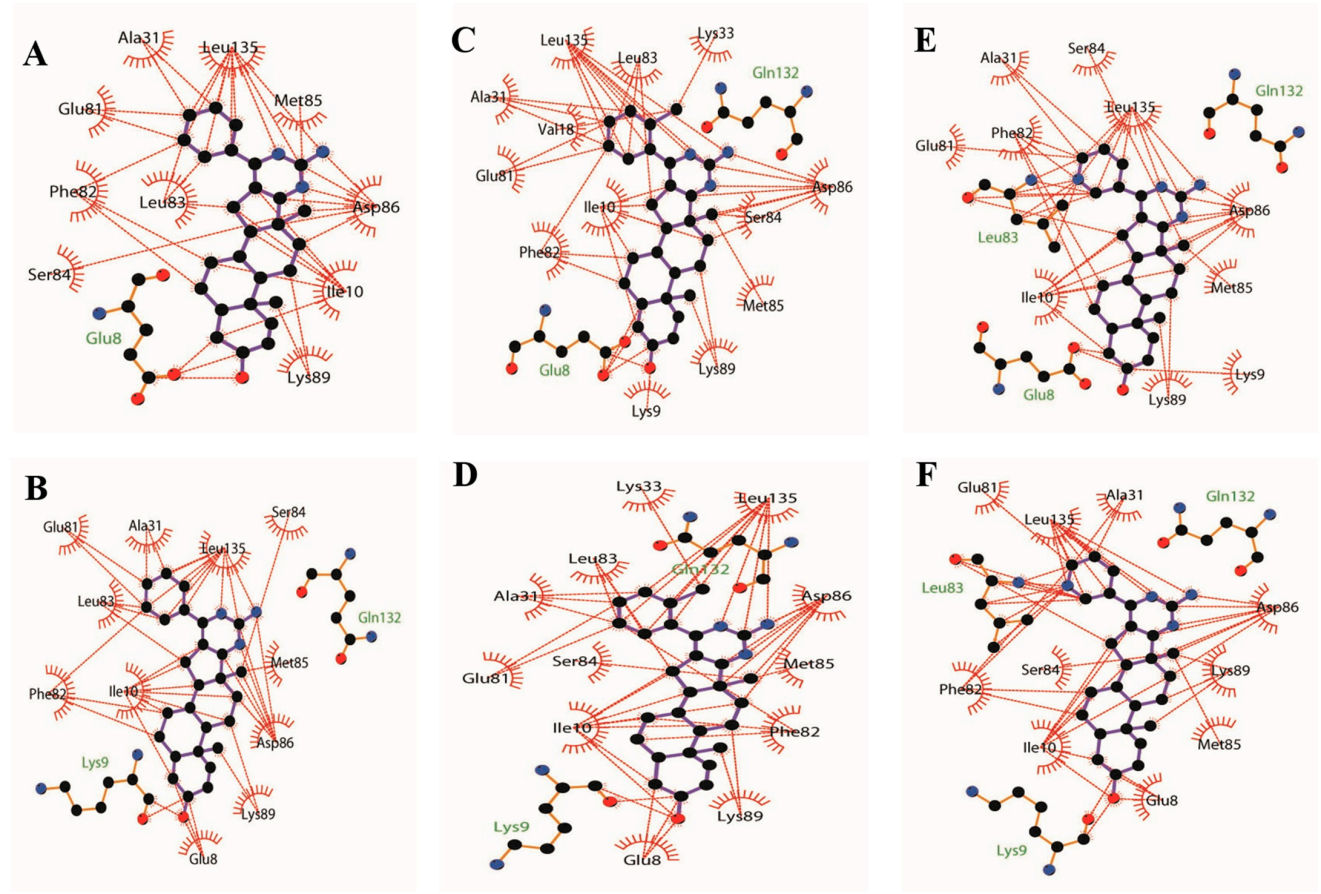
The 2D docked model of compounds **3a**, **3b**, **3l**, **6a**, **6b**, and **6l** into the binding pocket of CDK1 kinase (PDB code: 6GU6, black carbon atoms, red oxygen atoms, yellow sulphur atoms, and blue nitrogen atoms. Brick red dashed lines just display the hydrophobic interaction between the atoms and the amino acids of CDK1). (**A**) Hydrophobic interaction between the atoms of **3a** and the amino acids of CDK1; (**B**) Hydrophobic interaction between the atoms of **6a** and the amino acids of CDK1; (**C**) Hydrophobic interaction between the atoms of **3b** and the amino acids of CDK1; (**D**) Hydrophobic interaction between the atoms of **6b** and the amino acids of CDK1; (**E**) Hydrophobic interaction between the atoms of **3l** and the amino acids of CDK1; (**F**) Hydrophobic interaction between the atoms of **6l** and the amino acids of CDK1. Figures were drawn with LigPlot^+^.

As indicated in Table 2, compound **3a** displayed some inhibitory effect on the kinases of CDK1/CyclinA2, ALK, FGFR1, and FAK. In addition, it is widely known that the cyclin-dependent protein kinase (CDK) has diverse cellular roles, including regulation of the cell cycle and transcription and differentiation. It also plays a crucial role in regulation of the growth, proliferation, and differentiation of cancer cells. Blocking the CDK-driven pathway by inhibiting the intracellular tyrosine kinase domain of CDK has resulted in considerable improvements in tumor therapy. In particular, a variety of pyrimidine derivatives were synthesized and evaluated for their abilities to target CDK tyrosine kinases, such as AZD5438 (shown in Figure 1B) and dinaciciclib. In order to find a deeper explanation of the structure and inhibitory activities of these steroidal[17,16-*d*]pyrimidine derivatives, the possible docking modes of compounds **3a**, **3b**, **3l**, **6a**, **6b**, and **6l** with CDK1 (PDB code: 6GU6) were modeled (Figure 6, Figure 7, Figure 8 and Figure 9).

As shown in Figure 6B1,C1, Figure 7B1,C1 and Figure 8B1,C1, the six compounds **3a**, **3b**, **3l**, **6a**, **6b**, and **6l** almost bound to the outer edge of the ATP-binding pocket sandwiched between the P-loop (Gly 11-Glu 12-Gly 13-Thr 14-Phe 15-Gly 16) and active loop with the protein backbone from Gln 132 to Gly 145, but were far from the active segment spans with the protein backbone from Asp 146 to Glu 173 that can form a platform recognizing the CDK1 substrate residues to either side of the site phosphotransfer. In addition, the Lys 33-Glu12-Asp 146 triad in the bottom of the binding pocket of CDK1 also impacts the binding of the ATP adenine ring and phosphate moieties [77]. The hydrogen atom from the amidogen of the pyrimidine ring of compounds **3a**, **3b**, **3l**, **6a**, **6b**, and **6l** was far from Lys 33 with a different distance from 6.8 Å to 7.0 Å. These may be the reasons that compounds displayed lower inhibitory effects on the kinases of CDK1/CyclinA2.

It is clear that hydrogen bonds play an important role in the inhibition activities of compounds. The hydrogen atom from the amidogen of the pyrimidine ring of compounds **3a**, **3b**, **3l**, **6a**, **6b**, and **6l** can form one hydrogen bond with the oxygen atom of Gln 132 with the same distance of 2.1 Å shown in Figure 6B3,C3, Figure 7B3,C3 and Figure 8B3,C3. As indicated in Figure 6, Figure 7 and Figure 8, the hydroxyl group of compounds **3a**, **3b**, and **3l** was oriented toward the Glu8 of CDK1, but the hydroxyl group of compounds **6a**, **6b**, and **6l** was oriented toward the Lys 9 of CDK1. As a result, the hydrogen atom of the hydroxyl group of compounds **3a**, **3b**, and **3l** from epiandrosterone and compounds **6a**, **6b**, and **6l** from androsterone can form one different hydrogen bond with the oxygen atom of Glu 8 and Lys 9 with different distances, respectively. The hydrogen bond of the hydroxyl group from compound **3a** is stronger than **3b** and **3l** with a shorter length of 2.1 Å, 2.2 Å, and 2.3 Å, respectively. Meanwhile, the significant difference in efficacy of compound **6l** was mainly due to the longest distance (2.1 Å) of the hydrogen bond in the hydroxyl group, while the length of the hydrogen bond of the hydroxyl group of compounds **6a** and **6b** was 2.0 Å.

Additionally, hydrophobic forces also influence the interaction of inhibitors with kinases. A hydrophobic interaction between the non-polar residues of CDK1 and the atoms of steroidal[17,16-*d*]pyrimidine derivatives is shown in Figure 9. In this example, Ile 10, Phe 82, Lys 89, Asp 86, and Leu 83 interact with A, B, C, and D, four fused ring scaffolds of steroids. The methyl of C_19_ only interacts with Lys 89, while the methyl of C_18_ interacts with Ser 84, Met 85, and Asp 86. Both the methyls are oriented toward the hinge region of CDK1 with the protein backbone of Phe 80, Glu 81, Phe 82, Leu 83, Ser 84, Met 85, and Asp 86 (shown in Figure 6B2,C2, Figure 7B2,C2 and Figure 8B2,C2). The pyrimidine ring interacted with Ile10, Asp 86, and Leu 135, while the R-substituent group interacted with Ala 31, Glu 81, Phe 82, Leu 83, and Leu 135.

The analysis from above helps to explain that all the steroidal[17,16-*d*]pyrimidine derivatives **3a**–**l** exhibited higher inhibition activity than that of compounds **6a**–**l**, respectively. On the one hand, it occupied more space in the binding pocket when the hydroxyl group was oriented towards Glu 8 (shown in Figure 6B1,C1, Figure 7B1,C1 and Figure 8B1,C1). On the other hand, it had stronger hydrophobic force due to the skeleton of steroidal[17,16-*d*]pyrimidines near the hinge region of CDK1, especially the methyl of C_18_ (shown in Figure 6B3,C3, Figure 7B3,C3 and Figure 8B3,C3 and Figure 9). The above analysis also helped to explain why compound **3a** exhibited the highest potent inhibitory activities on all tested cell lines (Entry 13 in Table 1).

The analysis indicated that the configuration of hydroxy in the C3 position might be a key influence on the inhibitory activities of compounds. The molecular docking analyses were helpful in identifying the target steroidal[17,16-*d*]pyrimidines derived from epiandrosterone that could serve as potential lead compounds for the discovery of anticancer agents.

## 3. Conclusions

Twenty-four steroidal[17,16-d]pyrimidines derived from epiandrosterone(**3a**–**l**) and androsterone(**6a**–**l**) were designed and synthesized, and their in vitro inhibition activities on three cell lines were investigated. All the steroidal[17,16-d]pyrimidine derivatives **3a**–**l** exhibited higher inhibition activities against SGC-7901, A549, and HepG2 cell lines than that of compounds **6a**–**l** with the same substituents, respectively. Compound **3a**, containing a phenyl unit, exhibited the highest potential inhibitory activities against all tested cell lines with the IC_50_ values of 1.07 ± 0.22, 0.61 ± 0.19, and 0.51 ± 0.13 µg·mL^−1^. In addition, the detailed SARs analysis based on the inhibition activities, kinase assay, and molecular docking model demonstrated that the configuration of the hydroxyl group in the C_3_ position of A ring of steroidal scaffold might obviously affect the potential activities of these steroidal[17,16-d]pyrimidines. The β-configuration of the hydroxyl group of steroidal[17,16-d]pyrimidines performed better than an α-configuration of the hydroxyl group with the reason that it occupied more space in the binding pocket when the hydroxyl group was oriented towards Glu 8, and it also had stronger hydrophobic force due to the proximity of the skeleton of steroidal[17,16-d]pyrimidines to the hinge region of CDK1, and especially to the methyl of C_18_, which will provide key evidence for further structural optimization for the discovery of novel anticancer agents.

## 4. Experimental

### 4.1. Instrumentation and Chemicals

All starting materials and reagents are commercially available and were used without further purification unless otherwise specified. ^1^H NMR and ^13^C NMR spectra were recorded on a Bruker Avance III 600 MHz FT-NMR spectrometer using DMSO-*d*_6_ or CD_3_OD as the solvent and tetramethylsilane (TMS) as the internal standard. Chemical shifts were reported in *δ* (parts per million) values, and coupling constants ^n^*J* were reported in Hz. Mass spectra were performed on a WATERS ACQUITY UPLC^®^ H-CLASS PDA (Waters^®^) instrument. Analytical thin-layer chromatography was carried out on precoated silica gel plates GF254 (Qindao Haiyang Chemical, China), and spots were visualized with ultraviolet light. The calculated log*P* values (log*P*), which are the logarithms of the partition coefficients for octan-1-ol/water, were determined using the CS ChemOffice Ultra program (version 12.0, Cambridge-Soft, Cambridge, MA, USA).

### 4.2. Chemical Synthesis

#### 4.2.1. General Synthetic Procedure for Intermediates **2a**–**l** and **5a**–**l**

The intermediates **2a**–**l** and **5a**–**l** were synthesized via the classical aldol reaction. Generally, a solution of epiandrosterone or androsterone (1 mmol) in methanol (15 mL) was added to appropriate aldehyde (1.1 mmol) and sodium hydroxide (0.4 g, 10 mmol), respectively. The mixture was stirred at room temperature and detected by thin-layer chromatography. After the completion of this reaction, the mixture was poured into 40 mL of ice water with stirring. Then the precipitate was filtered and dried to obtain the corresponding powder **2a**–**l** and **5a**–**l**. Their basic physico-chemical properties and spectra data are as follows:

##### 16-Benzylidene-3-hydroxy-10,13-dimethyltetradecahydro-1H-cyclopenta[a]phenanthren-17(2H)-one **2a**

This compound was obtained following the above method as a white powder, yield 93%. MS (ESI) *m*/*z* 379.5 (M + H)^+^, calcd. for C_26_H_34_O_2_ *m*/*z* = 378.2.

##### 16-(2-Chlorobenzylidene)-3-hydroxy-10,13-dimethyltetradecahydro-1H-cyclopenta[a]phenanthren-17(2H)-one **2b**

This compound was obtained following the above method as a white powder, yield 92%. MS (ESI) *m*/*z* 413.4 (M + H)^+^, calcd. for C_26_H_33_ClO_2_ *m*/*z* = 412.2.

##### 16-(4-Chlorobenzylidene)-3-hydroxy-10,13-dimethyltetradecahydro-1H-cyclopenta[a]phenanthren-17(2H)-one **2c**

This compound was obtained following the above method as a white powder, yield 89%. MS (ESI) *m*/*z* 413.4 (M + H)^+^, calcd. for C_26_H_33_ClO_2_ *m*/*z* = 412.2.

##### 16-(2-Fluorobenzylidene)-3-hydroxy-10,13-dimethyltetradecahydro-1H-cyclopenta[a]phenanthren-17(2H)-one **2d**

This compound was obtained following the above method as a white powder, yield 94%. MS (ESI) *m*/*z* 397.4 (M + H)^+^, calcd. for C_26_H_33_FO_2_ *m*/*z* = 396.2.

##### 16-(4-Fluorobenzylidene)-3-hydroxy-10,13-dimethyltetradecahydro-1H-cyclopenta[a]phenanthren-17(2H)-one **2e**

This compound was obtained following the above method as a white powder, yield 92%. MS (ESI) *m*/*z* 397.4 (M + H)^+^, calcd. for C_26_H_33_FO_2_ *m*/*z* = 396.2.

##### 16-(2-Trifluorobenzylidene)-3-hydroxy-10,13-dimethyltetradecahydro-1H-cyclopenta[a]phenanthren-17(2H)-one **2f**

This compound was obtained following the above method as a white powder, yield 86%. MS (ESI) *m*/*z* 447.4 (M + H)^+^, calcd. for C_27_H_33_F_3_O_2_ *m*/*z* = 446.2.

##### 16-(4-Trifluorobenzylidene)-3-hydroxy-10,13-dimethyltetradecahydro-1H-cyclopenta[a]phenanthren-17(2H)-one **2g**

This compound was obtained following the above method as a white powder, yield 83%. MS (ESI) *m*/*z* 447.5 (M + H)^+^, calcd. for C_27_H_33_F_3_O_2_ *m*/*z* = 446.2.

##### 16-(2,4-Dichlorobenzylidene)-3-hydroxy-10,13-dimethyltetradecahydro-1H-cyclopenta[a]phenanthren-17(2H)-one **2h**

This compound was obtained following the above method as a white powder, yield 83%. MS (ESI) *m*/*z* 447.4 (M + H)^+^, calcd. for C_26_H_32_Cl_2_O_2_ *m*/*z* = 446.2.

##### 16-(2-Fluoro-4-bromobenzylidene)-3-hydroxy-10,13-dimethyltetradecahydro-1H-cyclopenta[a]phenanthren-17(2H)-one **2i**

This compound was obtained following the above method as a white powder, yield 90%. MS (ESI) *m*/*z* 475.4 (M + H)^+^, calcd. for C_26_H_32_BrFO_2_ *m*/*z* = 474.2.

##### 16-(4-Methoxybenzylidene)-3-hydroxy-10,13-dimethyltetradecahydro-1H-cyclopenta[a]phenanthren-17(2H)-one **2j**

This compound was obtained following the above method as a white powder, yield 93%. MS (ESI) *m*/*z* 409.5 (M + H)^+^, calcd. for C_27_H_36_O_3_ *m*/*z* = 408.3.

##### 16-(3,4,5-Trimethoxybenzylidene)-3-hydroxy-10,13-dimethyltetradecahydro-1H-cyclopenta[a]phenanthren-17(2H)-one **2k**

This compound was obtained following the above method as a white powder, yield 87%. MS (ESI) *m*/*z* 469.5 (M + H)^+^, calcd. for C_29_H_40_O_5_ *m*/*z* = 468.3.

##### 16-(3-Pyridinmethylene)-3-hydroxy-10,13-dimethyltetradecahydro-1H-cyclopenta[a]phenanthren-17(2H)-one **2l**

This compound was obtained following the above method as a white powder, yield 85%. MS (ESI) *m*/*z* 380.3 (M + H)^+^, calcd. for C_25_H_33_NO_2_ *m*/*z* = 379.3.

##### 16-Benzylidene-3-hydroxy-10,13-dimethyltetradecahydro-1H-cyclopenta[a]phenanthren-17(2H)-one **5a**

This compound was obtained following the above method as a white powder, yield 89%. MS (ESI) *m*/*z* 379.5 (M + H)^+^, calcd. for C_26_H_34_O_2_ *m*/*z* = 378.2.

##### 16-(2-Chlorobenzylidene)-3-hydroxy-10,13-dimethyltetradecahydro-1H-cyclopenta[a]phenanthren-17(2H)-one **5b**

This compound was obtained following the above method as a white powder, yield 91%. MS (ESI) *m*/*z* 413.4 (M + H)^+^, calcd. for C_26_H_33_ClO_2_ *m*/*z* = 412.2.

##### 16-(4-Chlorobenzylidene)-3-hydroxy-10,13-dimethyltetradecahydro-1H-cyclopenta[a]phenanthren-17(2H)-one **5c**

This compound was obtained following the above method as a white powder, yield 86%. MS (ESI) *m*/*z* 413.4 (M + H)^+^, calcd. for C_26_H_33_ClO_2_ *m*/*z* = 412.2.

##### 16-(2-Fluorobenzylidene)-3-hydroxy-10,13-dimethyltetradecahydro-1H-cyclopenta[a]phenanthren-17(2H)-one **5d**

This compound was obtained following the above method as a white powder, yield 90%. MS (ESI) *m*/*z* 397.4 (M + H)^+^, calcd. for C_26_H_33_FO_2_ *m*/*z* = 396.2.

##### 16-(4-Fluorobenzylidene)-3-hydroxy-10,13-dimethyltetradecahydro-1H-cyclopenta[a]phenanthren-17(2H)-one **5e**

This compound was obtained following the above method as a white powder, yield 93%. MS (ESI) *m*/*z* 397.4 (M + H)^+^, calcd. for C_26_H_33_FO_2_ *m*/*z* = 396.2.

##### 16-(2-Trifluorobenzylidene)-3-hydroxy-10,13-dimethyltetradecahydro-1H-cyclopenta[a]phenanthren-17(2H)-one **5f**

This compound was obtained following the above method as a white powder, yield 92%. MS (ESI) *m*/*z* 447.4 (M + H)^+^, calcd. for C_27_H_33_F_3_O_2_ *m*/*z* = 446.2.

##### 16-(4-Trifluorobenzylidene)-3-hydroxy-10,13-dimethyltetradecahydro-1H-cyclopenta[a]phenanthren-17(2H)-one **5g**

This compound was obtained following the above method as a white powder, yield 88%. MS (ESI) *m*/*z* 447.5 (M + H)^+^, calcd. for C_27_H_33_F_3_O_2_ *m*/*z* = 446.2.

##### 16-(2,4-Dichlorobenzylidene)-3-hydroxy-10,13-dimethyltetradecahydro-1H-cyclopenta[a]phenanthren-17(2H)-one **5h**

This compound was obtained following the above method as a white powder, yield 91%. MS (ESI) *m*/*z* 447.4 (M + H)^+^, calcd. for C_26_H_32_Cl_2_O_2_ *m*/*z* = 446.2.

##### 16-(2-Fluoro-4-bromobenzylidene)-3-hydroxy-10,13-dimethyltetradecahydro-1H-cyclopenta[a]phenanthren-17(2H)-one **5i**

This compound was obtained following the above method as a white powder, yield 94%. MS (ESI) *m*/*z* 475.4 (M + H)^+^, calcd. for C_26_H_32_BrFO_2_ *m*/*z* = 474.2.

##### 16-(4-Methoxybenzylidene)-3-hydroxy-10,13-dimethyltetradecahydro-1H-cyclopenta[a]phenanthren-17(2H)-one **5j**

This compound was obtained following the above method as a white powder, yield 84%. MS (ESI) *m*/*z* 409.5 (M + H)^+^, calcd. for C_27_H_36_O_3_ *m*/*z* = 408.3.

##### 16-(3,4,5-Trimethoxybenzylidene)-3-hydroxy-10,13-dimethyltetradecahydro-1H-cyclopenta[a]phenanthren-17(2H)-one **5k**

This compound was obtained following the above method as a white powder, yield 86%. MS (ESI) *m*/*z* 469.5 (M + H)^+^, calcd. for C_29_H_40_O_5_ *m*/*z* = 468.3.

##### 16-(3-Pyridinmethylene)-3-hydroxy-10,13-dimethyltetradecahydro-1H-cyclopenta[a]phenanthren-17(2H)-one **5l**

This compound was obtained following the above method as a white powder, yield 83%. MS (ESI) *m*/*z* 380.3 (M + H)^+^, calcd. for C_25_H_33_NO_2_ *m*/*z* = 379.3.

#### 4.2.2. General Synthetic Procedure for Target Compounds **3a**–**l** and **6a**–**l**

Guanidine nitrate (0.488 g, 4 mmol) and potassium *t*-butoxide (0.336 g, 3 mmol) were added to a solution of different intermediates **2a**–**l** or **5a**–**l** (1 mmol) in t-butanol (15 mL), which were heated under reflux overnight. After the completion of the reaction, the mixtures were poured into 50 mL of ice water with stirring, the pH value was tuned to 7–8, and then the precipitate was filtered and washed with water and dried under reduced pressure to obtain the crude compounds **3a**–**l** and **5a**–**l**, which were further purified by recrystallization or silica column chromatography. All compounds were fully characterized by the ESI-MS, ^1^H NMR, and ^13^C NMR spectrums, and their physico-chemical properties and spectra data are as follows:

##### 10-Amino-6a,8a-dimethyl-12-phenyl-tetradecahydro-1H-naphtho[2′,1′:4,5]indeno[1,2-d]pyrimidin-4-ol **3a**

This compound was obtained following the above method as a white powder, yield 63%. ^1^H NMR (600 MHz, DMSO-*d*_6_): *δ* 7.80 (dd, *J* = 8.1, 1.4 Hz, 2H), 7.45–7.41 (m, 3H), 6.46 (s, 2H, NH_2_), 4.46 (d, *J* = 4.7 Hz, 1H, C_3_-βOH), 3.34–3.30 (m, 1H, C_3_-αH), 2.66–2.62 (m, 1H), 2.58–2.53 (m, 1H), 1.98 (dd, *J* = 6.8, 4.1 Hz, 1H), 1.73–1.68 (m, 1H), 1.65–1.57 (m, 4H), 1.44–1.36 (m, 4H), 1.28–1.21 (m, 5H), 1.18–1.12 (m, 1H), 1.05–1.01 (m, 1H), 0.94 (s, 3H, CH_3_), 0.81 (s, 3H, CH_3_), 0.74–0.70 (m, 1H); ^13^C NMR (150 MHz, DMSO-*d*_6_): *δ* 183.45, 163.00, 158.98, 138.04, 129.34, 129.34, 128.25, 128.18, 117.92, 69.30, 54.95, 54.25, 45.45, 44.48, 38.18, 36.47, 35.41, 33.86, 33.06, 31.42, 31.14, 29.45, 28.29, 20.49, 17.11, 12.14; MS (ESI) *m*/*z* 418.5 (M + H)^+^, calcd. for C_27_H_35_N_3_O *m*/*z* = 417.3.

##### 10-Amino-12-(2-chlorophenyl)-6a,8a-dimethyl-tetradecahydro-1H-naphtho[2′,1′:4,5]indeno[1,2-d]pyrimidin-4-ol **3b**

This compound was obtained following the above method as yellowish powder, yield 71%. ^1^H NMR (600 MHz, CD_3_OD): *δ* 7.52 (dd, *J* = 7.7, 1.4 Hz, 1H), 7.44 (m, 1H), 7.38 (dd, *J* = 7.4, 1.9 Hz, 1H), 3.55–3.48 (m, 1H, C_3_-αH), 2.47–2.29 (m, 1H), 2.22–2.01 (m, 1H), 1.83–1.66 (m, 5H), 1.64–1.49 (m, 4H), 1.46–1.25 (m, 8H), 1.19–1.14 (m, 1H), 1.03 (s, 3H, CH_3_), 0.91 (s, 3H, CH_3_); ^13^C NMR (150 MHz, DMSO-*d*_6_): *δ* 182.46, 162.86, 159.54, 157.96, 137.38, 130.97, 130.56, 129.42, 127.14, 119.87, 69.28, 54.58, 54.22, 50.66, 45.74, 44.46, 38.16, 36.46, 35.40, 33.80, 32.90, 31.33, 28.24, 20.46, 17.10, 13.46, 12.12; MS (ESI) *m*/*z* 452.4 (M + H)^+^, calcd. for C_27_H_34_ClN_3_O *m*/*z* = 451.2.

##### 10-Amino-12-(4-chlorophenyl)-6a,8a-dimethyl-tetradecahydro-1H-naphtho[2′,1′:4,5]indeno[1,2-d]pyrimidin-4-ol **3c**

This compound was obtained following the above method as a pale white powder, yield 81%. ^1^H NMR (600 MHz, DMSO-*d*_6_): *δ* 7.83 (d, *J* = 8.6 Hz, 2H), 7.54 (d, *J* = 8.6 Hz, 2H), 6.54 (s, 1H, NH_2_), 4.48 (d, *J* = 4.1 Hz, 1H, C_3_-βOH), 3.34–3.30 (m, 1H, C_3_-αH), 2.79–2.53 (m, 2H), 2.00–1.97 (m, 1H), 1.74–1.71 (m, 1H), 1.69–1.57 (m, 5H), 1.47–1.38 (m, 4H), 1.27–1.24 (m, 4H), 1.19–1.13 (m, 1H), 1.08–1.04 (m, 1H), 0.94 (s, 3H, CH_3_), 0.81 (s, 3H, CH_3_); ^13^C NMR (150 MHz, DMSO-*d*_6_): δ 183.73, 162.93, 157.60, 136.82, 134.10, 129.94, 128.34, 117.95, 69.27, 54.86, 54.22, 45.42, 44.47, 38.14, 36.44, 35.39, 33.86, 33.00, 31.34, 29.31, 28.24, 20.44, 17.07, 14.14, 12.12; MS (ESI) *m*/*z* 452.4 (M + H)^+^, calcd. for C_27_H_34_ClN_3_O *m*/*z* = 451.2.

##### 10-Amino-12-(2-fluorophenyl)-6a,8a-dimethyl-tetradecahydro-1H-naphtho[2′,1′:4,5]indeno[1,2-d]pyrimidin-4-ol **3d**

This compound was obtained following the above method as a white powder, yield 83%. ^1^H NMR (600 MHz, DMSO-*d*_6_): *δ* 7.56–7.47 (m, 2H), 7.33–7.28 (m, 2H), 6.58 (s, 2H, NH_2_), 4.47 (d, *J* = 4.6 Hz, 1H, C_3_-βOH), 3.34–3.30 (m, 1H, C_3_-αH), 2.38–2.26 (m, 2H), 2.00–1.95 (m, 1H), 1.67–1.57 (m, 6H), 1.45–1.38 (m, 4H), 1.27–1.20 (m, 4H), 1.17–1.11 (m, 2H), 1.06–1.03 (m, 1H), 0.94 (s, 3H, CH_3_), 0.80 (s, 3H, CH_3_); ^13^C NMR (150 MHz, DMSO-*d*_6_): *δ* 182.73, 163.01, 156.21, 131.07 (dd, *J* = 12.9, 6.9 Hz), 126.04, 124.42, 120.12, 115.92, 115.78, 99.52, 69.26, 54.54, 54.20, 50.65, 45.71, 44.46, 38.14, 36.44, 35.39, 33.80, 32.94, 31.34, 31.07, 28.23, 20.43, 17.07, 12.10; MS (ESI) *m*/*z* 436.5 (M + H)^+^, calcd. for C_27_H_34_FN_3_O *m*/*z* = 435.3.

##### 10-Amino-12-(4-fluorophenyl)-6a,8a-dimethyl-tetradecahydro-1H-naphtho[2′,1′:4,5]indeno[1,2-d]pyrimidin-4-ol **3e**

This compound was obtained following the above method as a white powder, yield 75%. ^1^H NMR (600 MHz, DMSO-*d*_6_): *δ* 7.90–7.84 (m, 2H), 7.31 (t, *J* = 8.9 Hz, 2H), 6.51 (s, 2H, NH_2_), 4.48 (d, *J* = 4.4 Hz, 1H, C_3_-βOH), 3.34–3.30 (m, 1H, C_3_-αH), 2.69–2.55 (m, 2H), 2.01–1.94 (m, 1H), 1.75–1.70 (m, 1H), 1.69–1.57 (m, 5H), 1.44–1.37 (m, 4H), 1.29–1.24 (m, 4H), 1.19–1.13 (m, 2H), 1.08–1.02 (m, 1H), 0.95 (s, 3H, CH_3_), 0.82 (s, 3H, CH_3_); ^13^C NMR (150 MHz, DMSO-*d*_6_): *δ* 184.06, 163.38, 158.29, 130.88(d, *J* = 8.6 Hz), 118.20, 115.72, 115.58, 69.75, 55.37, 54.71, 45.89, 44.95, 38.62, 36.92, 35.87, 34.33, 33.49, 31.86, 31.59, 29.83, 28.72, 20.93, 17.55, 12.60; MS (ESI) *m*/*z* 436.5 (M + H)^+^, calcd. for C_27_H_34_FN_3_O *m*/*z* = 435.3.

##### 10-Amino-12-(2-trifluoromethylphenyl)-6a,8a-dimethyl-tetradecahydro-1H-naphtho[2′,1′:4,5]indeno[1,2-d]pyrimidin-4-ol **3f**

This compound was obtained following the above method as a white powder, yield 82%. ^1^H NMR (600 MHz, DMSO-*d*_6_): *δ* 7.87–7.36 (m, 4H), 6.50 (s, 2H, NH_2_), 4.43 (s, 1H, C_3_-βOH), 3.36–3.34 (m, 1H, C_3_-αH), 2.39–2.35 (m, 1H), 2.13 (d, *J* = 9.2 Hz, 1H), 2.00–1.96 (m, 1H), 1.86–1.79 (m, 1H), 1.75–1.71 (m, 1H), 1.60–1.56 (m, 6H), 1.48–1.38 (m, 5H), 1.26–1.21 (m, 4H), 0.90 (s, 3H, CH_3_), 0.79 (s, 3H, CH_3_); ^13^C NMR (150 MHz, DMSO-*d*_6_): *δ* 182.42, 161.70, 160.03, 132.83 (d, *J* = 2.2 Hz), 130.70, 129.27, 126.83, 126.75, 119.63, 69.73, 54.35, 51.13, 47.56, 44.86, 38.60, 37.07, 35.77, 35.74, 35.00, 31.82, 30.98, 28.63, 21.84, 20.56, 17.49, 13.91, 12.56; MS (ESI) *m*/*z* 486.4 (M + H)^+^, calcd. for C_28_H_34_F_3_N_3_O *m*/*z* = 485.3.

##### 10-Amino-12-(4-trifluoromethylphenyl)-6a,8a-dimethyl-tetradecahydro-1H-naphtho[2′,1′:4,5]indeno[1,2-d]pyrimidin-4-ol **3g**

This compound was obtained following the above method as a pale white powder, yield 82%. ^1^H NMR (600 MHz, DMSO-*d*_6_): *δ* 8.01 (d, *J* = 8.1 Hz, 2H), 7.84 (d, *J* = 8.3 Hz, 2H), 6.57 (s, 2H, NH_2_), 4.43 (d, *J* = 4.5 Hz, 1H, C_3_-βOH), 3.37–3.34 (m, 1H, C_3_-αH), 2.71–2.67 (m, 1H), 2.62–2.58 (m, 1H), 1.99 (d, *J* = 8.3 Hz, 1H), 1.74–1.72 (m, 1H), 1.68–1.60 (m, 5H), 1.45–1.41 (m, 4H), 1.30–1.24 (m 4H), 1.17–1.04 (m, 3H), 0.96 (s, 3H, CH_3_), 0.82 (s, 3H, CH_3_); ^13^C NMR (150 MHz, DMSO-*d*_6_): *δ* 183.96, 163.02, 157.36, 141.94, 130.74, 128.90, 125.19 (d, *J* = 3.6 Hz), 118.53, 69.27, 54.84, 54.21, 45.49, 44.47, 38.14, 36.44, 35.39, 33.86, 32.97, 31.38, 31.11, 29.19, 28.23, 20.44, 17.08, 12.11; MS (ESI) *m*/*z* 486.3 (M + H)^+^, calcd. for C_28_H_34_F_3_N_3_O *m*/*z* = 485.3.

##### 10-Amino-12-(2,4-dichlorophenyl)-6a,8a-dimethyl-tetradecahydro-1H-naphtho[2′,1′:4,5]indeno[1,2-d]pyrimidin-4-ol **3h**

This compound was obtained following the above method as a pale white powder, yield 74%. ^1^H NMR (600 MHz, DMSO-*d*_6_): *δ* 7.72 (d, *J* = 2.0 Hz, 1H), 7.50 (dd, *J* = 8.3, 2.1 Hz, 1H), 7.43 (d, *J* = 8.3 Hz, 1H), 6.58 (s, 2H, NH_2_), 4.43 (d, *J* = 3.7 Hz, 1H, C_3_-βOH), 3.37–3.34 (m, 1H, C_3_-αH), 2.31–2.26 (m, 1H), 2.20–2.17 (m, 1H), 2.00–1.96 (m, 1H), 1.67–1.58 (m, 6H), 1.46–1.40 (m, 4H), 1.27–1.20 (m, 4H), 1.15–1.03 (m, 3H), 0.92 (s, 3H, CH_3_), 0.80 (s, 3H, CH_3_); ^13^C NMR (150 MHz, DMSO-*d*_6_): *δ* 182.70, 162.85, 158.43, 136.30, 133.90, 132.10, 131.92, 128.96, 127.41, 119.89, 69.26, 54.54, 54.20, 45.74, 44.45, 38.14, 36.44, 35.38, 33.78, 32.85, 31.37, 31.06, 28.22, 27.59, 20.43, 17.07, 12.09; MS (ESI) *m*/*z* 486.3 (M + H)^+^, calcd. for C_27_H_33_Cl_2_N_3_O *m*/*z* = 485.2.

##### 10-Amino-12-(2-fluoro-4-bromophenyl)-6a,8a-dimethyl-tetradecahydro-1H-naphtho[2′,1′:4,5]indeno[1,2-d]pyrimidin-4-ol **3i**

This compound was obtained following the above method as a pale white powder, yield 82%. ^1^H NMR (600 MHz, DMSO-*d*_6_): *δ* 7.66 (dd, *J* = 9.9, 1.6 Hz, 1H), 7.56–7.46 (m, 2H), 6.58 (s, 2H, NH_2_), 4.43 (d, *J* = 4.4 Hz, 1H, C_3_-βOH), 3.36–3.33 (m, 1H, C_3_-αH), 2.37–2.27 (m, 2H), 2.00–1.97 (m 1H), 1.64–1.60(m, 6H), 1.44–1.40 (m, 4H), 1.27–1.21 (m, 4H), 1.15–1.03 (m, 3H), 0.93 (s, 3H, CH_3_), 0.80 (s, 3H, CH_3_); ^13^C NMR (150 MHz, DMSO-*d*_6_): *δ* 183.49, 163.49, 160.33, 158.59, 155.62, 133.01, 128.22, 123.14,128.22, 123.14, 120.58, 119.88, 69.74, 54.98, 54.66, 46.20, 44.93, 38.62, 36.91, 35.86, 34.27, 33.39, 31.85, 31.54, 28.70, 28.35, 20.90, 17.52, 12.58; MS (ESI) *m*/*z* 514.3 (M + H)^+^, calcd. for C_27_H_33_BrFN_3_O *m*/*z* = 513.2.

##### 10-Amino-12-(4-methoxyphenyl)-6a,8a-dimethyl-tetradecahydro-1H-naphtho[2′,1′:4,5]indeno[1,2-d]pyrimidin-4-ol **3j**

This compound was obtained following the above method as a white powder, yield 89%. ^1^H NMR (600 MHz, DMSO-*d*_6_): *δ* 7.78 (d, *J* = 8.7 Hz, 2H), 7.00 (d, *J* = 8.7 Hz, 2H), 4.45 (d, *J* = 4.5 Hz, 1H, C_3_-βOH), 3.79 (s, 3H, OCH_3_), 3.37–3.31 (m, 1H, C_3_-αH), 2.66–2.54 (m, 2H), 1.97–1.90 (m, 1H), 1.70 (dd, *J* = 8.2, 4.0 Hz, 1H), 1.66–1.57 (m, 4H), 1.43–1.34 (m, 4H), 1.27–1.20 (m, 4H), 1.17–0.99 (m, 3H), 0.91 (s, 3H, CH_3_), 0.79 (s, 3H, CH_3_), 0.73–0.67 (m, 1H); ^13^C NMR (150 MHz, DMSO-*d*_6_): *δ* 183.19, 162.87, 160.22, 158.56, 130.41, 129.71, 117.16, 113.60, 69.29, 55.22, 54.94, 54.26, 45.34, 44.46, 38.19, 36.46, 35.40, 33.85, 33.07, 31.42, 31.13, 29.67, 28.30, 20.80, 17.10, 12.12; MS (ESI) *m*/*z* 448.4 (M + H)^+^, calcd. for C_28_H_37_N_3_O_2_ *m*/*z* = 447.3.

##### 10-Amino-12-(3,4,5-trimethoxyphenyl)-6a,8a-dimethyl-tetradecahydro-1H-naphtho[2′,1′:4,5]indeno[1,2-d]pyrimidin-4-ol **3k**

This compound was obtained following the above method as a white powder, yield 91%. ^1^H NMR (600 MHz, DMSO-*d*_6_): *δ* 7.08 (s, 2H), 6.41 (s, 2H, NH_2_), 4.44 (s, 1H, C_3_-βOH), 3.81 (s, 6H, OCH_3_), 3.70 (s, 3H, OCH_3_), 3.34–3.29 (m, 1H, C_3_-αH), 2.71 (t, *J* = 13.0 Hz, 1H), 2.60 (m, 1H), 1.97 (d, *J* = 9.9 Hz, 1H), 1.74 (dd, *J* = 8.2, 4.0 Hz, 1H), 1.67–1.56 (m, 4H), 1.45–1.35 (m, 4H), 1.32–1.19 (m, 4H), 1.18–0.99 (m, 3H), 0.94 (s, 3H, CH_3_), 0.79 (s, 3H, CH_3_), 0.75–0.69 (m, 1H); ^13^C NMR (150 MHz, DMSO-*d*_6_): *δ* 183.44, 162.84, 158.80, 152.62, 138.56, 133.50, 117.83, 105.65, 69.31, 60.09, 55.91, 55.03, 54.25, 45.47, 44.50, 38.18, 36.46, 35.39, 33.84, 33.07, 31.39, 31.07, 29.59, 28.28, 20.48, 17.06, 12.09; MS (ESI) *m*/*z* 508.5 (M + H)^+^, calcd. for C_30_H_41_N_3_O_4_ *m*/*z* = 507.3.

##### 10-Amino-12-(3-pyridinyl)-6a,8a-dimethyl-tetradecahydro-1H-naphtho[2′,1′:4,5]indeno[1,2-d]pyrimidin-4-ol **3l**

This compound was obtained following the above method as a white powder, yield 81%. ^1^H NMR (600 MHz, DMSO-*d*_6_): *δ* 8.99 (d, *J* = 1.6 Hz, 1H), 8.64 (dd, *J* = 4.8, 1.6 Hz, 1H), 8.17 (dt, *J* = 7.9, 1.9 Hz, 1H), 7.53–7.49 (m, 1H), 6.56 (s, 2H, NH_2_), 4.44 (d, *J* = 4.6 Hz, 1H, C_3_-βOH), 3.38–3.34 (m, 1H, C_3_-αH), 2.73–2.69 (m, 1H), 2.61–2.58 (m, 1H), 1.99 (d, *J* = 8.5 Hz, 1H), 1.77–1.73 (m, 1H), 1.68–1.61 (m, 5H), 1.47–1.41 (m, 4H), 1.28–1.25 (m, 4H), 1.18–1.04 (m, 3H), 0.97 (s, 3H, CH_3_), 0.83 (s, 3H, CH_3_); ^13^C NMR (150 MHz, DMSO-*d*_6_): *δ* 184.23, 163.49, 157.02, 150.54, 149.46, 135.92, 134.02, 123.93, 118.92, 69.75, 55.37, 54.70, 45.96, 44.95, 38.62, 36.92, 35.87, 34.33, 33.44, 31.86, 31.55, 29.47, 28.72, 20.92, 17.56, 12.59; MS (ESI) *m*/*z* 419.4 (M + H)^+^, calcd. for C_26_H_34_N_4_O *m*/*z* = 418.3.

##### 10-Amino-6a,8a-dimethyl-12-phenyl-tetradecahydro-1H-naphtho[2′,1′:4,5]indeno[1,2-d]pyrimidin-4-ol **6a**

This compound was obtained following the above method as a yellowish powder, yield 82%. ^1^H NMR (600 MHz, DMSO-*d*_6_): *δ* 7.81 (dd, *J* = 8.0, 1.4 Hz, 2H), 7.46 (m, 3H), 6.44 (s, 2H, NH_2_), 4.16 (d, *J* = 2.4 Hz, 1H, C_3_-αOH), 3.82–3.79 (m, 1H, C_3_-βH), 2.70–2.56 (m, 2H), 2.03–1.96 (m, 1H), 1.77–1.72 (m, 1H), 1.70–1.63 (m, 2H), 1.56–1.49 (m, 3H), 1.48–1.37 (m, 4H), 1.35–1.28 (m, 3H), 1.25–1.14 (m, 4H), 0.96 (s, 3H, CH_3_), 0.81 (s, 3H, CH_3_); ^13^C NMR (150 MHz, DMSO-*d*_6_): *δ* 183.98, 163.43, 159.42, 138.52, 129.76, 129.76, 128.69, 128.62, 118.46, 64.52, 55.50, 54.88, 45.90, 39.10, 36.39, 36.22, 34.34, 33.53, 32.26, 31.68, 29.83, 29.13, 28.61, 20.51, 17.56, 11.63; MS (ESI) *m*/*z* 418.4 (M + H)^+^, calcd. for C_27_H_35_N_3_O *m*/*z* = 417.3.

##### 10-Amino-12-(2-chlorophenyl)-6a,8a-dimethyl-tetradecahydro-1H-naphtho[2′,1′:4,5]indeno[1,2-d]pyrimidin-4-ol **6b**

This compound was obtained following the above method as a white powder, yield 81%. ^1^H NMR (600 MHz, DMSO-*d*_6_): *δ* 7.60–7.14 (m, 4H), 6.53 (s, 2H, NH_2_), 4.16 (d, *J* = 2.4 Hz, 1H, C_3_-αOH), 2–3.76 (m, 1H, C_3_-βH), 2.71–2.51 (m, 1H), 2.40–2.16 (m, 1H), 2.02–1.86 (m, 1H), 1.84–1.72 (m, 1H), 1.67–1.59 (m, 2H), 1.58–1.43 (m, 5H), 1.38–1.22 (m, 6H), 1.19–1.06 (m, 3H), 0.93 (s, 3H, CH_3_), 0.78 (s, 3H, CH_3_); ^13^C NMR (150 MHz, DMSO-*d*_6_): *δ* 182.51, 159.51, 137.37, 130.97, 130.15, 129.40, 128.24, 119.89, 99.53, 64.03, 54.37, 48.65, 45.73, 44.40, 35.91, 33.79, 32.84, 31.17, 28.62, 28.04, 20.01, 17.09, 15.58, 14.08, 13.45, 11.13; MS (ESI) *m*/*z* 452.6 (M + H)^+^, calcd. for C_27_H_34_ClN_3_O *m*/*z* = 451.2.

##### 10-Amino-12-(4-chlorophenyl)-6a,8a-dimethyl-tetradecahydro-1H-naphtho[2′,1′:4,5]indeno1,2-d]pyrimidin-4-ol **6c**

This compound was obtained following the above method as a white powder, yield 88%. ^1^H NMR (600 MHz, DMSO-*d*_6_): *δ* 7.84 (d, *J* = 8.6 Hz, 1H), 7.65 (d, *J* = 8.6 Hz, 1H), 7.52 (dd, *J* = 19.8, 8.6 Hz, 2H), 6.49 (s, 2H, NH_2_), 4.17 (d, *J* = 6.6, 3.7 Hz, 1H, C_3_-αOH), 3.82–3.79 (m, 1H, C_3_-βH), 2.79–2.59 (m, 1H), 2.02–1.81 (m, 1H), 1.78–1.71 (m, 1H), 1.67–1.58 (m, 2H), 1.56–1.45 (m, 4H), 1.42–1.23 (m, 8H), 1.20–1.15 (m, 2H), 1.04–0.97 (m, 1H), 0.86 (s, 3H, CH_3_), 0.79 (s, 3H, CH_3_); ^13^C NMR (150 MHz, DMSO-*d*_6_): *δ* 208.47, 137.22, 134.06, 131.93, 130.36, 129.94, 128.82, 128.34, 64.03, 54.00, 48.71, 47.01, 45.42, 38.55, 35.87, 35.70, 34.18, 31.85, 31.31, 30.68, 28.62, 28.02, 19.70, 14.15, 11.14; MS (ESI) *m*/*z* 452.6 (M + H)^+^, calcd. for C_27_H_34_ClN_3_O *m*/*z* = 451.2.

##### 10-Amino-12-(2-fluorophenyl)-6a,8a-dimethyl-tetradecahydro-1H-naphtho[2′,1′:4,5]indeno[1,2-d]pyrimidin-4-ol **6d**

This compound was obtained following the above method as a white powder, yield 79%. ^1^H NMR (600 MHz, DMSO-*d*_6_): *δ* 7.74–7.08 (m, 4H), 6.52 (s, 2H, NH_2_), 4.19 (s, 1H, C_3_-αOH), 3.82–3.79 (m, 1H, C_3_-βH), 2.45–2.25 (m, 2H), 2.02–1.70 (m, 2H), 1.68–1.56 (m, 3H), 1.55–1.37 (m, 6H), 1.35–1.21 (m, 5H), 1.18–1.12 (m, 2H), 0.94 (s, 3H, CH_3_), 0.79 (s, 3H, CH_3_); ^13^C NMR (150 MHz, DMSO-*d*_6_): *δ* 182.79, 163.03, 159.97, 158.33, 156.21, 131.08(dd, *J* = 16.4, 5.9Hz), 126.06, 124.44, 120.16, 115.94, 64.05, 54.37, 54.06, 50.73, 47.12, 45.74, 35.93, 34.54, 33.82, 31.38, 28.64, 28.06, 21.36, 19.66, 17.09, 13.47, 11.16; MS (ESI) *m*/*z* 436.6 (M + H)^+^, calcd. for C_27_H_34_FN_3_O *m*/*z* = 435.3.

##### 10-Amino-12-(4-fluorophenyl)-6a,8a-dimethyl-tetradecahydro-1H-naphtho[2′,1′:4,5]indeno[1,2-d]pyrimidin-4-ol **6e**

This compound was obtained following the above method as a white powder, yield 83%. ^1^H NMR (600 MHz, DMSO-*d*_6_): *δ* 7.88 (dd, *J* = 8.8, 5.6 Hz, 1H), 7.69 (dd, *J* = 8.6, 5.6 Hz, 1H), 7.32–7.27 (m, 2H), 6.46 (s, 2H, NH_2_), 4.17 (d, *J* = 3.0 Hz, 1H, C_3_-αOH), 3.82–3.79 (m, 1H, C_3_-βH), 2.77–2.58 (m, 1H), 2.01–1.80 (m, 1H), 1.78–1.72 (m, 1H), 1.69–1.61 (m, 2H), 1.60–1.43 (m, 4H), 1.41–1.22 (m, 7H), 1.20–1.14 (m, 2H), 1.03–0.94 (m, 2H), 0.86 (s, 3H, CH_3_), 0.79 (s, 3H, CH_3_); ^13^C NMR (150 MHz, DMSO-*d*_6_): *δ* 208.51, 162.90, 136.16, 132.55 (d, *J* = 8.5 Hz), 130.56, 115.88, 115.73, 64.03, 54.02, 48.80, 46.97, 38.54, 35.87, 35.70, 31.85, 31.85, 31.35, 31.30, 30.67, 28.65, 28.02, 19.71, 17.08, 14.16, 11.14; MS (ESI) *m*/*z* 436.6 (M + H)^+^, calcd. for C_27_H_34_FN_3_O *m*/*z* = 435.3.

##### 10-Amino-12-(2-trifluoromethylphenyl)-6a,8a-dimethyl-tetradecahydro-1H-naphtho[2′,1′:4,5]indeno[1,2-d]pyrimidin-4-ol **6f**

This compound was obtained following the above method as a white powder, yield 81%. ^1^H NMR (600 MHz, DMSO-*d*_6_): *δ* 7.81 (d, *J* = 7.8 Hz, 1H), 7.72 (t, *J* = 7.5 Hz, 1H), 7.63 (t, *J* = 7.7 Hz, 1H), 7.41 (d, *J* = 7.6 Hz, 1H), 6.48 (s, 2H, NH_2_), 4.18 (d, *J* = 3.0 Hz, 1H, C_3_-αOH), 3.82–3.77 (d, *J* = 2.6 Hz, 1H, C_3_-βH), 2.17–2.08 (m, 2H), 2.01–1.97 (m, 1H), 1.69–1.63 (m, 1H), 1.60–1.44 (m, 7H), 1.39–1.21 (m, 6H), 1.14–1.06 (m, 2H), 0.90 (s, 3H, CH_3_), 0.85–0.80 (m, 1H), 0.77 (s, 3H, CH_3_); ^13^C NMR (150 MHz, DMSO-*d*_6_): *δ* 182.73, 162.77, 160.90, 137.59, 132.81, 130.50, 129.32, 126.72 (dd, *J* = 8.8, 4.7 Hz), 123.51, 119.80, 64.52, 55.16, 54.85, 46.27, 39.07, 36.37, 36.17, 34.24, 33.33, 32.25, 31.64, 29.10, 28.55, 27.68, 20.47, 17.52, 11.58; MS (ESI) *m*/*z* 486.6 (M + H)^+^, calcd. for C_28_H_34_F_3_N_3_O *m*/*z* = 485.3.

##### 10-Amino-12-(4-trifluoromethylphenyl)-6a,8a-dimethyl-tetradecahydro-1H-naphtho[2′,1′:4,5]indeno[1,2-d]pyrimidin-4-ol **6g**

This compound was obtained following the above method as a white powder, yield 75%. ^1^H NMR (600 MHz, DMSO-*d*_6_): *δ* 8.02 (d, *J* = 8.1 Hz, 2H), 7.84 (d, *J* = 8.3 Hz, 2H), 6.57 (s, 2H, NH_2_), 4.17 (d, *J* = 2.9 Hz, 1H, C_3_-αOH), 3.82–3.80 (m, C_3_-βH), 2.71–2.66 (m, 1H), 2.63–2.59 (m, 1H), 2.00 (d, *J* = 9.6 Hz, 1H), 1.76–1.72 (m, 1H), 1.68–1.63 (m, 2H), 1.59–1.53 (m, 2H), 1.50–1.42 (m, 4H), 1.37–1.28 (m, 4H), 1.26–1.22 (m, 2H), 1.19–1.14 (m, 2H), 0.97 (s, 3H, CH_3_), 0.80 (s, 3H, CH_3_); ^13^C NMR (150 MHz, DMSO-*d*_6_): *δ* 184.49, 163.50, 157.83, 142.43, 129.41, 125.59 (d, *J* = 5.3 Hz), 123.77, 119.04, 64.72, 55.29, 54.83, 45.97, 36.39, 36.18, 34.62, 32.26, 31.94, 29.59, 29.38, 29.09, 28.59, 17.60, 17.52, 11.62, 11.58; MS (ESI) *m*/*z* 486.4 (M + H)^+^, calcd. for C_28_H_34_F_3_N_3_O *m*/*z* = 485.3.

##### 10-Amino-12-(2,4-dichlorophenyl)-6a,8a-dimethyl-tetradecahydro-1H-naphtho[2′,1′:4,5]indeno[1,2-d]pyrimidin-4-ol **6h**

This compound was obtained following the above method as a white powder, yield 81%. ^1^H NMR (600 MHz, DMSO-*d*_6_): *δ* 7.72 (d, *J* = 2.1 Hz, 1H), 7.54–7.49 (m, 1H), 7.45–7.39 (m, 1H), 6.58 (s, 2H, NH_2_), 4.17 (d, *J* = 7.9 Hz, 1H, C_3_-αOH), 3.81–3.78 (m, 1H, C_3_-βH), 2.31–2.17 (m, 1H), 2.03–1.70 (m, 2H), 1.68–1.61 (m, 2H), 1.55–1.43 (m, 6H), 1.37–1.20 (m, 7H), 1.18–1.12 (m, 2H), 0.92 (s, 3H, CH_3_), 0.78 (s, 3H, CH_3_); ^13^C NMR (150 MHz, DMSO-*d*_6_): *δ* 182.77, 162.86, 158.43, 152.40, 136.38, 136.31, 133.93, 133.90, 132.12, 119.93, 75.80, 64.26, 54.39, 45.75, 35.72 (dd, *J* = 37.8, 26.5 Hz), 31.76, 31.53, 28.25, 28.12, 17.11, 17.05, 11.18, 11.09, 10.99; MS (ESI) *m*/*z* 486.3 (M + H)^+^, calcd. for C_27_H_33_Cl_2_N_3_O *m*/*z* = 485.2.

##### 10-Amino-12-(2-fluoro-4-bromophenyl)-6a,8a-dimethyl-tetradecahydro-1H-naphtho[2′,1′:4,5]indeno[1,2-d]pyrimidin-4-ol **6i**

This compound was obtained following the above method as a yellowish powder, yield 71%. ^1^H NMR (600 MHz, DMSO-*d*_6_): *δ* 7.69–7.64 (m, 1H), 7.55–7.47 (m, 2H), 6.58 (s, 2H, NH_2_), 4.17 (d, *J* = 1.8 Hz, 1H, C_3_-αOH), 3.81–3.79 (m, 1H, C_3_-βH), 2.41–2.24 (m, 2H), 2.02–1.96 (m, 1H), 1.66–1.63 (m, 2H), 1.55–1.41 (m, 7H), 1.35–1.22 (m, 6H), 1.18–1.13 (m, 2H), 0.94 (s, 3H, CH_3_), 0.79 (s, 3H, CH_3_); ^13^C NMR (150 MHz, DMSO-*d*_6_): δ 183.09, 163.03, 155.15, 132.57, 127.77, 125.46, 122.78, 120.15, 119.42, 119.25, 64.05, 54.36, 50.73, 45.75, 35.93, 35.32, 34.54, 33.82, 32.94, 31.79, 31.20, 28.63, 28.13, 19.66, 17.07, 13.47, 11.16; MS (ESI) *m*/*z* 514.3 (M + H)^+^, calcd. for C_27_H_33_BrFN_3_O *m*/*z* = 513.2.

##### 10-Amino-12-(4-methoxyphenyl)-6a,8a-dimethyl-tetradecahydro-1H-naphtho[2′,1′:4,5]indeno[1,2-d]pyrimidin-4-ol **6j**

This compound was obtained following the above method as a yellowish powder, yield 70%. ^1^H NMR (600 MHz, DMSO-*d*_6_): *δ* 7.69 (dd, *J* = 8.8 Hz, 2H), 7.01 (dd, *J* = 8.7, 6.0 Hz, 2H), 6.36 (s, 2H, NH_2_), 4.17 (d, *J* = 4.7 Hz, 1H, C_3_-αOH), 3.81 (s, 3H, OCH_3_), 3.80–3.79 (m, 1H, C_3_-βH), 2.62 (dd, *J* = 14.6, 9.2 Hz, 1H), 1.99–1.81 (m, 1H), 1.75 (d, *J* = 11.3 Hz, 1H), 1.64 (dd, *J* = 20.8, 9.5 Hz, 2H), 1.59–1.45 (m, 4H), 1.44–1.37 (m, 3H), 1.35–1.23 (m, 5H), 1.22–1.16 (m, 2H), 1.03–0.98 (m, 1H), 0.94 (s, 3H, CH_3_), 0.80 (s, 3H, CH_3_); ^13^C NMR (150 MHz, DMSO-*d*_6_): *δ* 183.73, 162.89, 160.61, 159.06, 130.56, 130.10, 117.92, 114.01, 64.48, 55.57, 55.45, 54.73, 45.69, 36.21, 35.90, 34.14, 33.33, 32.11, 31.52, 29.88, 28.78, 28.43, 20.34, 17.34, 11.46; MS (ESI) *m*/*z* 448.4 (M + H)^+^, calcd. for C_28_H_37_N_3_O_2_ *m*/*z* = 447.3.

##### 10-Amino-12-(3,4,5-trimethoxyphenyl)-6a,8a-dimethyl-tetradecahydro-1H-naphtho[2′,1′:4,5]indeno[1,2-d]pyrimidin-4-ol **6k**

This compound was obtained following the above method as a white powder, yield 72%. ^1^H NMR (600 MHz, DMSO-*d*_6_): *δ* 7.08 (s, 2H), 6.42 (s, 2H, NH_2_), 4.17 (d, *J* = 2.4 Hz, 1H, C_3_-αOH), 3.82 (s, 6H, OCH_3_), 3.81–3.79 (s, 1H, C_3_-βH), 3.71 (s, 3H, OCH_3_), 2.75–2.70 (m, 1H), 2.64–2.58 (m, 1H), 2.02–1.94 (m, 1H), 1.80–1.75 (m, 1H), 1.72–1.63 (m, 2H), 1.62–1.45 (m, 4H), 1.44–1.38 (m, 3H), 1.37–1.27 (m, 3H), 1.26–1.20 (m, 2H), 1.19–1.10 (m, 2H), 0.97 (s, 3H, CH_3_), 0.80 (s, 3H, CH_3_); ^13^C NMR (150 MHz, DMSO-*d*_6_): *δ* 183.50, 162.84, 158.80, 152.62, 138.49, 133.52, 117.91, 105.67, 64.30, 60.16, 59.61, 57.01, 56.52, 56.17, 55.68, 55.32, 54.40, 45.48, 35.93, 31.82, 31.41, 28.15, 17.10, 17.04, 11.18, 11.10; MS (ESI) *m*/*z* 508.4 (M + H)^+^, calcd. for C_30_H_41_N_3_O_4_ *m*/*z* = 507.3.

##### 10-Amino-12-(3-pyridinyl)-6a,8a-dimethyl-tetradecahydro-1H-naphtho[2′,1′:4,5]indeno[1,2-d]pyrimidin-4-ol **6l**

This compound was obtained following the above method as a white powder, yield 61%. ^1^H NMR (600 MHz, DMSO-*d*_6_): *δ* 8.98 (d, *J* = 1.6 Hz, 1H), 8.64 (dd, *J* = 4.8, 1.6 Hz, 1H), 8.16 (dt, *J* = 7.9, 1.9 Hz, 1H), 7.51 (dd, *J* = 7.7, 5.1 Hz, 1H), 6.55 (s, 2H, NH_2_), 4.17 (d, *J* = 2.2 Hz, 1H, C_3_-αOH), 3.82–3.79 (m, 1H, C_3_-βH), 2.73–2.68 (m, 1H), 2.63–2.58 (m, 1H), 2.03–1.96 (m, 1H), 1.77–1.73 (m, 1H), 1.70–1.64 (m, 2H), 1.59–1.52 (m, 2H), 1.50–1.42 (m, 4H), 1.40–1.28 (m, 4H), 1.26–1.20 (m, 2H), 1.19–1.12 (m, 2H), 0.96 (s, 3H, CH_3_), 0.80 (s, 3H, CH_3_); ^13^C NMR (150 MHz, DMSO-*d*_6_): *δ* 183.82, 163.03, 156.55, 150.09, 149.10, 135.46, 133.57, 123.53, 118.49, 64.27, 54.43, 54.40, 45.49, 35.92, 35.80, 35.78, 31.80, 31.43, 30.94, 30.94, 28.91, 28.70, 28.13, 17.11, 17.06, 11.20, 11.12; MS (ESI) *m*/*z* 419.5 (M + H)^+^, calcd. for C_26_H_34_N_4_O *m*/*z* = 418.3.

### 4.3. In Vitro Cytotoxicity Assay

The in vitro cytotoxicity of the synthesized compounds against various human cancer cell lines was measured by the MTT [3-(4,5-dimethylthiazol-2-yl)-2,5-diphenyltetrazoliumbromide] colorimetric method [76], and the general procedures were reported in previous publications [63,64,67,78]. All the data of the experiment were analyzed according to SPSS software, and the 50% inhibitory concentrations (IC_50_) of each compound for the different cell lines were determined. All assays were performed in triplicate on three independent experiments, and measurement data were expressed as the mean ± S.D.

### 4.4. Kinase Activity Assay

The kinase inhibitory activities of compounds were determined by ADP-Glo Protocol or LANCE Protocol. Enzymes, substrate, ATP, and inhibitors were diluted in the kinase buffer, which contained 40 mM Tris, pH 7.5; 20 mM MgCl_2_; 0.1 mg/mL BSA; and 50 μM DTT.

ADP-Glo protocol: (1) Add to the wells of 384 low volume plate: 1 μL of inhibitor or (5% DMSO), 2 μL of enzymes, and 2 μL of substrate/ATP mix; (2) Incubate at 25 °C; (3) Add 5 μL of ADP-Glo™ Reagent; (4) Incubate at 25 °C for 40 min; (5) Add 10 μL of Kinase Detection Reagent; (6) Incubate at 25 °C for 30 min; (7) Record luminescence (Integration time 0.5 s, RLU); (8) Calculate the enzyme Inhibition rate: Inhibition% = (RLU_(Sample)_ ™ RLU_(1%DMSO)_)/(RLU_(Blank)_ ™ RLU_(1%DMSO)_) × 100%.

LANCE protocol: (1) Add to the wells of a white Optiplate-384: 5 µL of enzyme, 2.5 µL of inhibitor or kinase buffer, 2.5 µL of Ulight-4E-BP1 or Ulight- CREB/ATP mix; (2) Incubate at 25 °C for 2 h; (3) Stop kinase reactions by adding 5 µL of 40 mM EDTA prepared in 1X detection buffer (stop solution); (4) Add 5 µL of 4X detection mix (Eu-anti-phospho-tyrosine antibody at a final concentration of 2 nM); (5) Cover with TopSeal-A and incubate for 1 h at 25 °C; (6) Remove TopSeal-A and read signal with the Nivo Reader in TR-FRET mode (excitation at 320 nm and emission at 665 and 615 nm); (7) Calculate enzyme inhibition rate: Ratio = 665/615, Inhibition% = (Ratio_Sample_ ™ Ratio_DMSO_)/(Ratio_Blank_ ™ Ratio_DMSO_) × 100%. Dilute the kinase, ATP, inhibitors, and Ulight-4E-BP1 or Ulight-CREB peptide in kinase buffer. Prepare a 4X detection mix by diluting the Eu-anti-phospho-tyrosine antibody to 8 nM in 1X LANCE detection buffer.

### 4.5. Molecular Docking Study

All the molecular docking simulations were carried out by the AutoDock 4.2 software [79]. The docking tutorial we used and the detailed AutoDock basic operation methods can be found at: https://autodock.scripps.edu/ (accessed on 6 January 2023). The protein preparation process of flexible docking mainly includes fixing the exact residues, removing irrelevant water molecules, adding hydrogen atoms and adding charges, etc. The crystal structure (PDB: 6GU6, https://www.rcsb.org/structure/6GU6, accessed on 6 January 2023) of the CDK1 bond to **3a**, **3b**, **3l**, **6a**, **6b**, and **6l** were used in the docking studies. We first removed inhibitor dinaciclib from the crystal structure, then put the target molecules **3a**, **3b**, **3l**, **6a**, **6b**, and **6l** in the binding site, and the energy was optimized using a genetic algorithm. Only the best-scoring ligand-protein complex was used for the binding site analysis. All the docking results were processed and modified in PyMOL 1.7.4.5 software (https://pymol.org, accessed on 6 January 2023) and LigPlot^+^ v.2.2.7 software (https://www.ebi.ac.uk/thornton-srv/software/LigPlus/, accessed on 6 January 2023).

## Figures and Tables

**Table 1:** Cytotoxic activity of the steroidal derivatives against human tumor cells.

Entry	Compd. No.	Substrates (R)	In Vitro Cytotoxicity IC_50_ ^a^ (μg/mL)		
			SGC-7901 ^b^	A549 ^b^	HepG2 ^b^
1	**2a**	Ph	5.13 ± 1.59	11.74 ± 1.72	4.74 ± 1.46
2	**2b**	2-ClPh	2.19 ± 0.49	3.24 ± 0.42	1.80 ± 0.08
3	**2c**	4-ClPh	8.80 ± 1.46	12.52 ± 2.71	8.02 ± 1.38
4	**2d**	2-FPh	3.19 ± 0.38	6.81 ± 0.72	1.71 ± 0.39
5	**2e**	4-FPh	8.45 ± 1.20	8.94 ± 1.58	4.46 ± 0.66
6	**2f**	2-CF_3_Ph	22.03 ± 0.61	11.38 ± 2.14	8.13 ± 1.43
7	**2g**	4-CF_3_Ph	21.28 ± 3.07	24.93 ± 3.60	10.58 ± 0.75
8	**2h**	2,4-Cl_2_Ph	1.94 ± 0.22	2.79 ± 0.49	1.31 ± 0.38
9	**2i**	2-F-4-BrPh	2.04 ± 0.09	4.08 ± 0.42	0.80 ± 0.16
10	**2j**	4-CH_3_OPh	13.25 ± 0.97	10.70 ± 0.78	6.63 ± 1.10
11	**2k**	3,4,5-(CH_3_O)_3_Ph	3.73 ± 0.39	3.17 ± 0.74	3.17 ± 0.54
12	**2l**	3-Py	>40	>40	>40
13	**3a**	Ph	1.07 ± 0.22	0.61 ± 0.19	0.51 ± 0.13
14	**3b**	2-ClPh	1.39 ± 0.20	1.37 ± 0.17	1.31 ± 0.30
15	**3c**	4-ClPh	2.45 ± 0.49	6.22 ± 0.98	1.09 ± 0.36
16	**3d**	2-FPh	1.79 ± 0.42	0.53 ± 0.15	0.56 ± 0.10
17	**3e**	4-FPh	2.75 ± 0.51	2.91 ± 0.58	1.49 ± 0.38
18	**3f**	2-CF_3_Ph	4.11 ± 0.73	2.91 ± 0.54	2.46 ± 0.54
19	**3g**	4-CF_3_Ph	3.77 ± 0.86	2.90 ± 0.41	1.85 ± 0.12
20	**3h**	2,4-Cl_2_Ph	2.20 ± 0.24	2.57 ± 0.45	2.10 ± 0.44
21	**3i**	2-F-4-BrPh	3.05 ± 0.50	2.96 ± 0.70	2.16 ± 0.12
22	**3j**	4-CH_3_OPh	3.26 ± 0.21	4.00 ± 0.91	1.39 ± 0.09
23	**3k**	3,4,5-(CH_3_O)_3_Ph	2.86 ± 0.49	2.36 ± 0.45	0.43 ± 0.15
24	**3l**	3-Py	2.74 ± 0.67	2.83 ± 0.66	0.55 ± 0.15
25	**5a**	Ph	3.53 ± 0.60	2.28 ± 0.25	1.21 ± 0.37
26	**5b**	2-ClPh	19.16 ± 3.11	12.92 ± 0.90	7.59 ± 1.86
27	**5c**	4-ClPh	5.14 ± 1.03	5.06 ± 1.06	3.44 ± 0.89
28	**5d**	2-FPh	4.21 ± 0.68	5.17 ± 1.06	2.20 ± 0.53
29	**5e**	4-FPh	2.38 ± 0.57	3.72 ± 0.35	2.19 ± 0.27
30	**5f**	2-CF_3_Ph	13.95 ± 2.33	10.34 ± 1.80	5.50 ± 0.98
31	**5g**	4-CF_3_Ph	>40	>40	>40
32	**5h**	2,4-Cl_2_Ph	>40	>40	>40
33	**5i**	2-F-4-BrPh	>40	>40	>40
34	**5j**	4-CH_3_OPh	>40	>40	>40
35	**5k**	3,4,5-(CH_3_O)_3_Ph	4.26 ± 0.86	6.13 ± 0.89	6.67 ± 1.08
36	**5l**	3-Py	>40	>40	>40
37	**6a**	Ph	16.33 ± 2.06	19.93 ± 1.92	14.26 ± 1.82
38	**6b**	2-ClPh	4.01 ± 0.31	6.04 ± 0.71	3.91 ± 0.69
39	**6c**	4-ClPh	8.67 ± 1.38	11.43 ± 2.20	6.80 ± 1.06
40	**6d**	2-FPh	4.67 ± 1.09	7.85 ± 1.82	4.76 ± 1.12
41	**6e**	4-FPh	6.65 ± 1.60	13.30 ± 0.45	6.13 ± 0.92
42	**6f**	2-CF_3_Ph	4.94 ± 0.95	9.08 ± 1.22	6.14 ± 0.84
43	**6g**	4-CF_3_Ph	>40	>40	33.38 ± 2.08
44	**6h**	2,4-Cl_2_Ph	5.75 ± 0.95	8.36 ± 0.50	6.78 ± 1.48
45	**6i**	2-F-4-BrPh	7.69 ± 1.74	17.57 ± 1.51	7.79 ± 1.05
46	**6j**	4-CH_3_OPh	12.38 ± 1.43	>40	12.79 ± 2.40
47	**6k**	3,4,5-(CH_3_O)_3_Ph	5.23 ± 0.94	9.86 ± 1.15	9.52 ± 2.00
48	**6l**	3-Py	>40	>40	18.69 ± 2.43
49	**Epiandrosterone**	-	20.31 ± 2.59	11.53 ± 1.36	17.25 ± 2.65
50	**Androsterone**	-	>40	>40	>40
51	**5-FU ^c^**	-	8.81 ± 1.91	7.58 ± 0.67	7.79 ± 0.65

^a^ IC_50_—Compound concentration required to inhibit tumor cell proliferation by 50%. ^b^ Abbreviations: SGC-7901—human gastric cancer cell line, A549—human lung cancer cell line, HepG2—human hepatocellular liver carcinoma cell line. ^c^ 5-Fluorouracil, used as a positive control.

**Table 2:** Selectivity profiling of compound **3a** against a panel of tyrosine kinases.

Kinase	Inhibition Rate at 10 μM (%)	Kinase	Inhibition Rate at 10 μM (%)	Kinase	Inhibition Rate at 10 μM (%)
ALK	17.36 ^a^	EGFR(T790M)	−2.43 ^a^	PDGFRα(D842V)	−33.57 ^a^
CDK1/CyclinA2	22.51 ^a^	FAK	10.52 ^a^	PIM1	−14.26 ^a^
CDK2/CyclinE1	−26.89 ^a^	FGFR1	11.82 ^a^	PIM2	3.72 ^b^
CDK6/CyclinD1	−4.98 ^b^	MET	−4.06 ^a^	ROS	−12.98 ^a^
EGFR	−2.96 ^a^	PDGFRα	−11.73 ^a^	ROS(G2032)	−17.44 ^a^

^a^—Data was determined by ADP-Glo Protocol; ^b^—Data was determined by LANCE Protocol.

## Data Availability

Not applicable.

## Supplementary Materials

The following are available online at https://www.mdpi.com/article/10.3390/molecules28062691/s1, Figures S1–S48. all the ^1^H NMR and ^13^C NMR spectroscopy for target compounds **3a**–**l** and **6a**–**l**.

## Sample Availability

Samples of the compounds are available from the authors.
