# Supplementary material for: Novel Steroidal[17,16-d]pyrimidines Derived from Epiandrosterone and Androsterone: Synthesis, Characterization and Configuration-Activity Relationships
Source: Molecules. 2023 Mar 16;28(6):2691. doi: 10.3390/molecules28062691 (PMC10054084; doi:10.3390/molecules28062691)
Supplement: Supplementary file 1 [file molecules-28-02691-s001.zip › molecules-2273661-supplementary.pdf]

Supplementary Materials

Novel Steroidal[17,16-*d*]pyrimidines Derived from Epiandrosterone and Androsterone: Synthesis, Characterization and Configuration-Activity Relationships

Fei Yang [1,2], Fang Liu [2], Yong Min [2], Liqiao Shi [2], Manli Liu [2], Kaimei Wang [2], Shaoyong Ke [2,*], Yan Gong [2,*] and Ziwen Yang [1,2,*]

[1] College of Life Sciences, Wuhan University, Wuhan 430072, China; feiy_2017@whu.edu.cn
[2] Key Laboratory of Microbial Pesticides, Ministry of Agriculture and Rural Affairs, National Biopesticide Engineering Research Centre, Hubei Biopesticide Engineering Research Centre, Hubei Academy of
Agricultural Sciences, Wuhan 430064, China; fang.liu@nberc.com (F.L.); yong.min@nberc.com (Y.M.);
liqiao.shi@nberc.com (L.S.); manli.liu@nberc.com (M.L.); kaimei.wang@nberc.com (K.W.)
* Correspondence: shaoyong.ke@nberc.com (S.K.); gongyan@nberc.com (Y.G.); ziwen.yang@nberc.com (Z.Y.)

Supporting Information

Experimental section

Instrumentation and chemicals

All starting materials and reagents commercially available were used without further purification, unless otherwise specified. ^{1}H NMR and ^{13}C NMR spectra were recorded on a Bruker Avance III 600 MHz FT-NMR spectrometer using DMSO-d_6 or CD_3OD as the solvent and tetramethylsilane (TMS) as the internal standard. Chemical shifts are reported in δ (parts per million) values, and coupling constants ^nJ are reported in Hz. Mass spectra were performed on a WATERS ACQUITY UPLC® H-CLASS PDA (Waters®) instrument. Analytical thin-layer chromatography was carried out on precoated silica gel plates GF254 (Qindao Haiyang Chemical, China), and spots were visualized with ultraviolet light. The calculated log*P* values (log*P*), which are the logarithms of the partition coefficients for octan-1-ol/water, were determined using the CS ChemOffice Ultra program (version 12.0, Cambridge-Soft, Cambridge, MA, USA).

Spectroscopy for target compounds

Structures of target compounds **3a-l** and **6a-l** were confirmed by their ^{1}H NMR, ^{13}C NMR, ESI-MS, and their ^{1}H NMR, ^{13}C NMR and ESI-MS were consistent with the assigned structures. The typical ^{1}H NMR and ^{13}C NMR data for synthesized compounds have been presented in the following, which can confirm the result.

Compd 3a

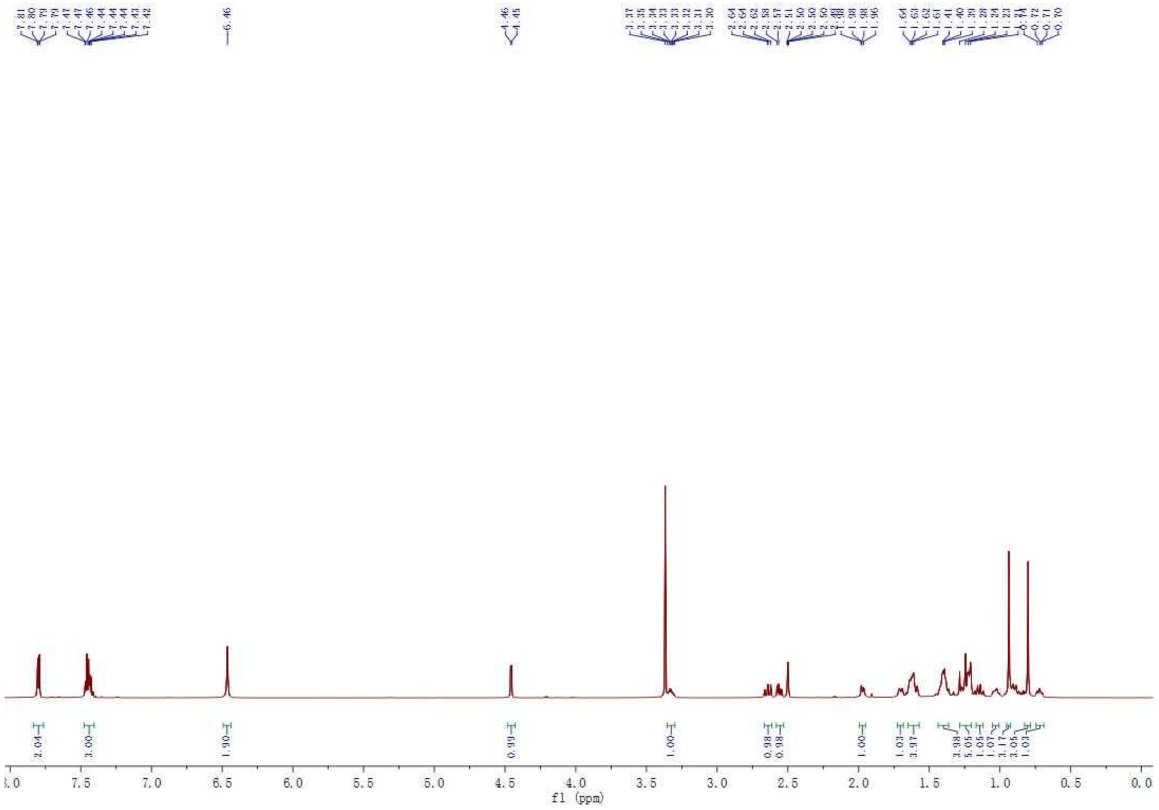

Figure S1: ^{1}H NMR spectrum for compound 3a.

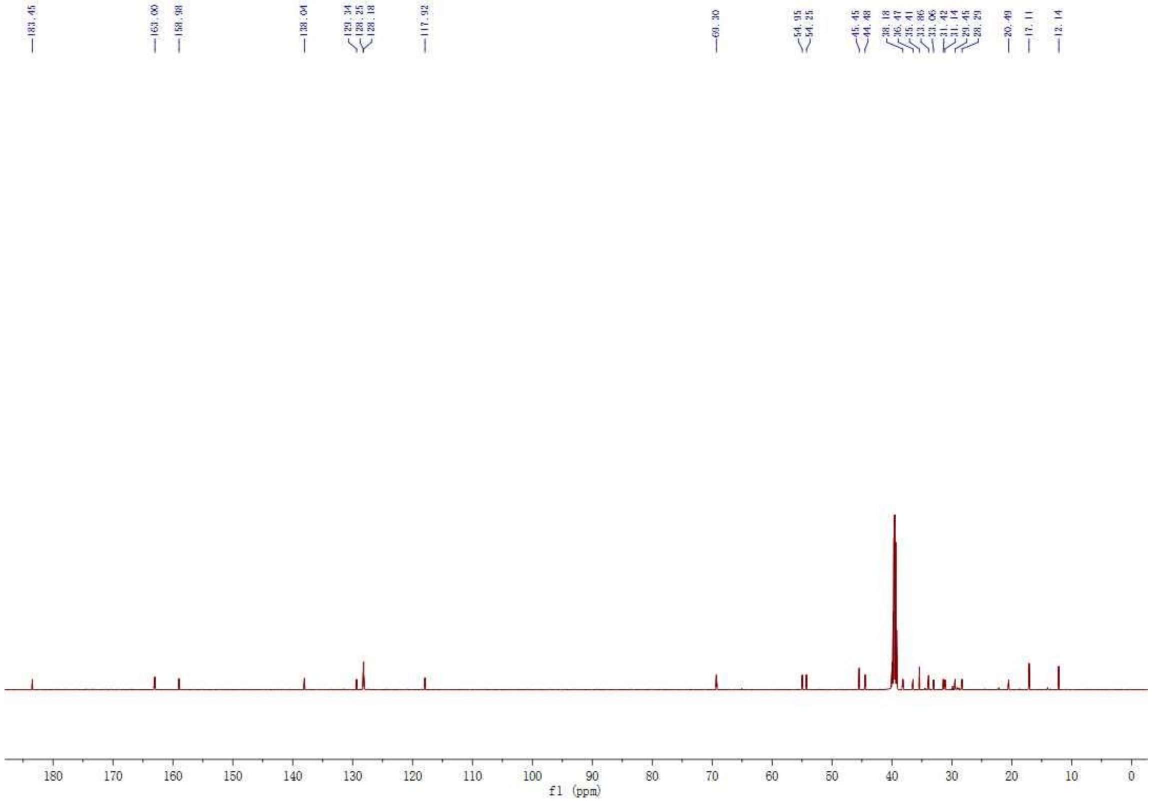

Figure S2: ^{13}C NMR spectrum for compound 3a.

Compd 3b

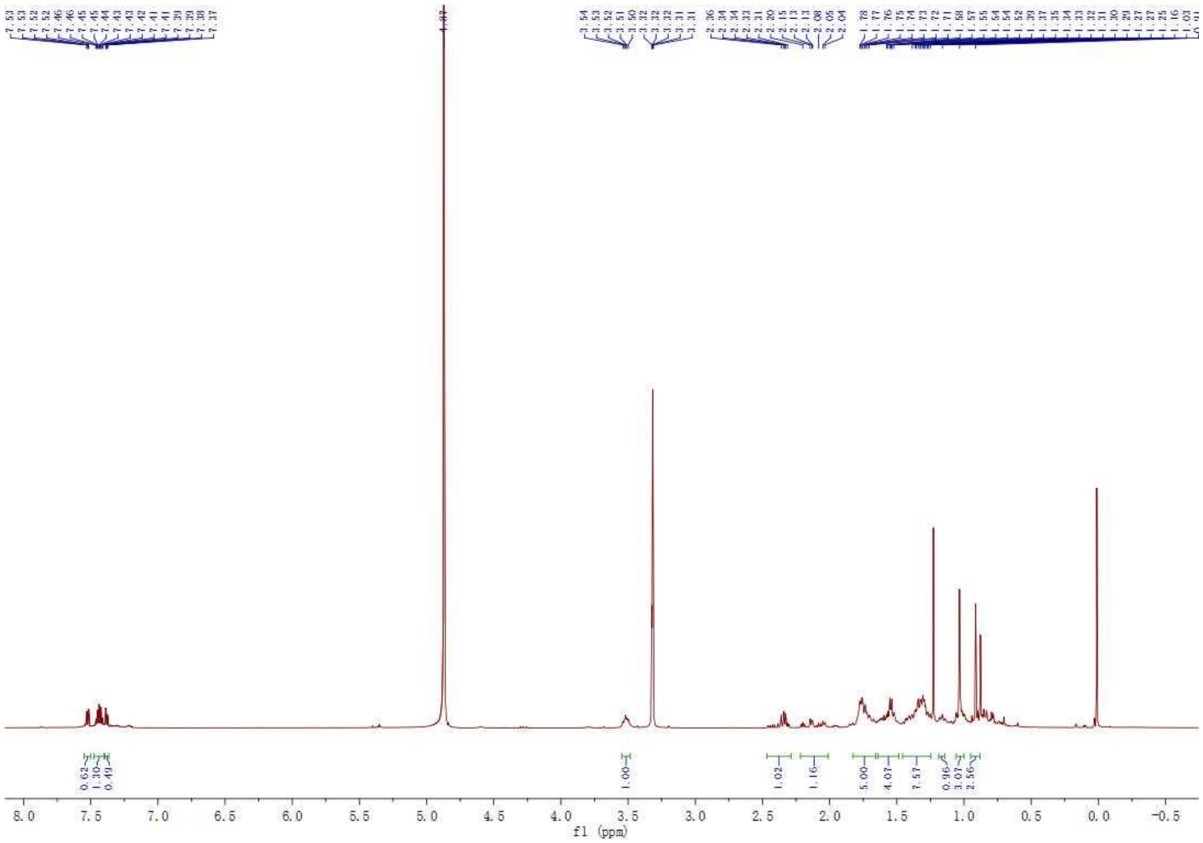

Figure S3: ^{1}H NMR spectrum for compound 3b.

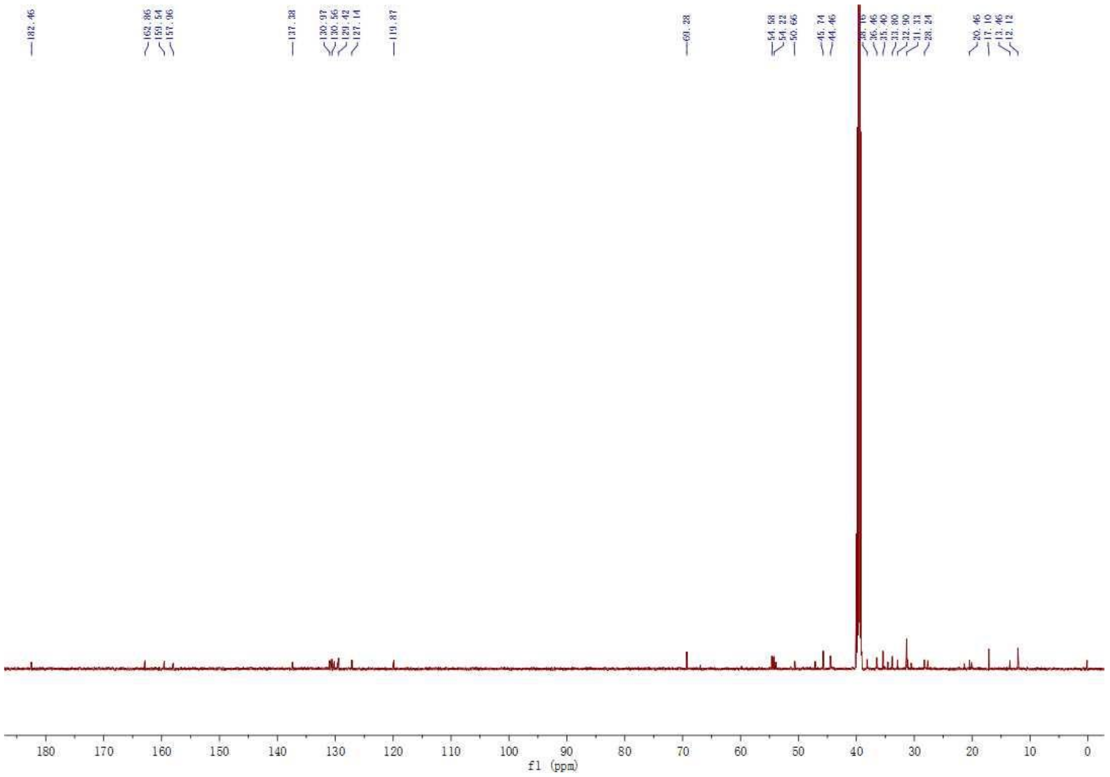

Figure S4: ^{13}C NMR spectrum for compound 3b.

Compd 3c

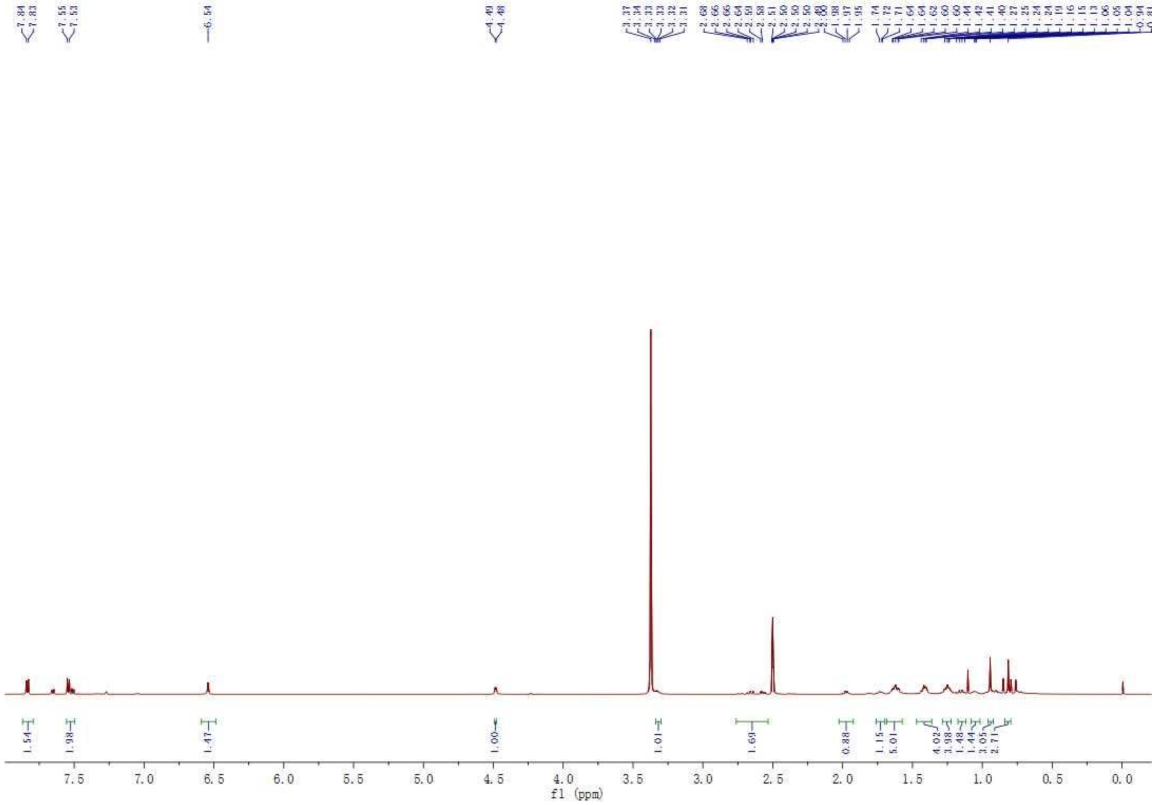

Figure S5: ^{1}H NMR spectrum for compound 3c.

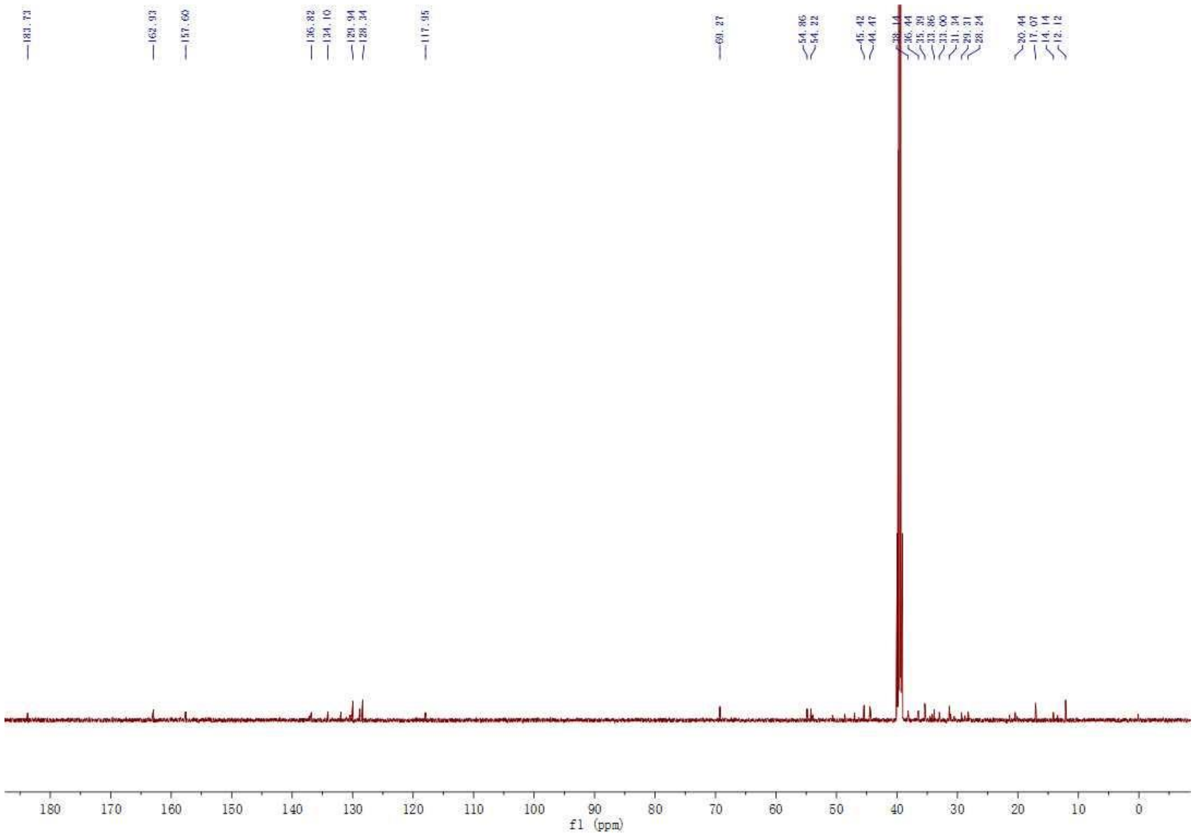

Figure S6: ^{13}C NMR spectrum for compound 3c.

Compd 3d

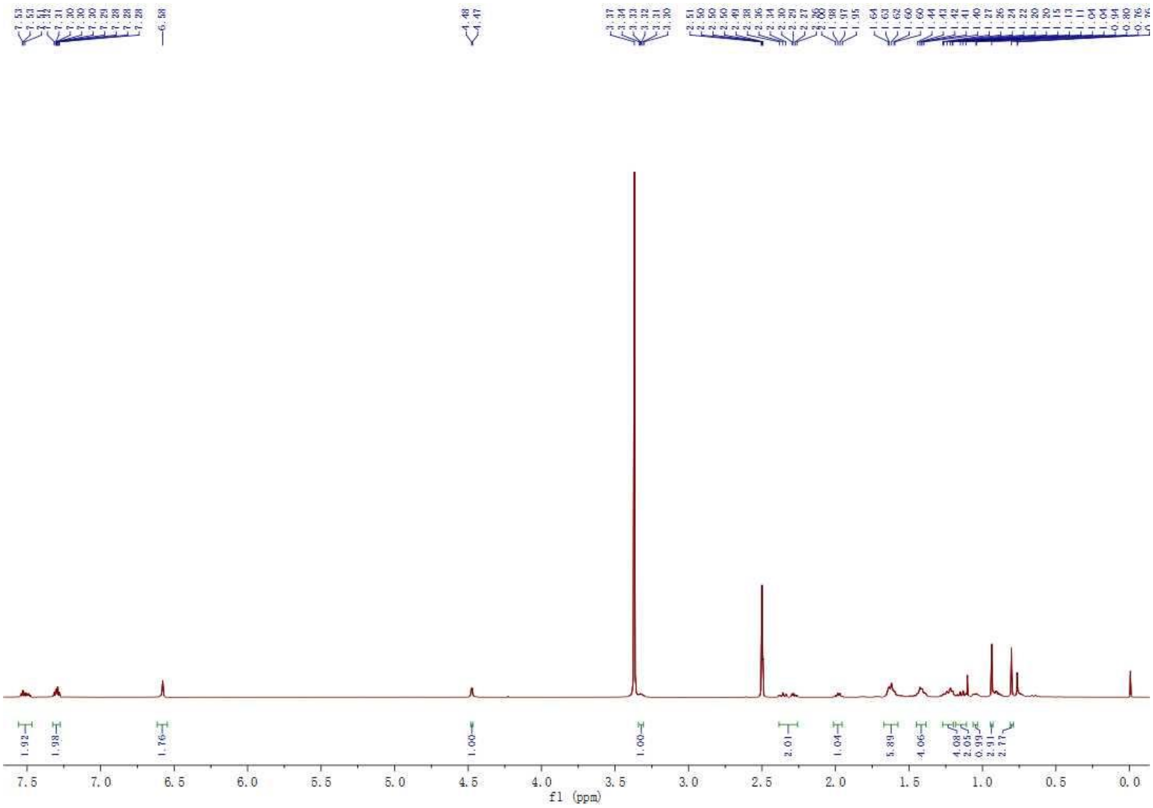

Figure S7: ^1H NMR spectrum for compound 3d.

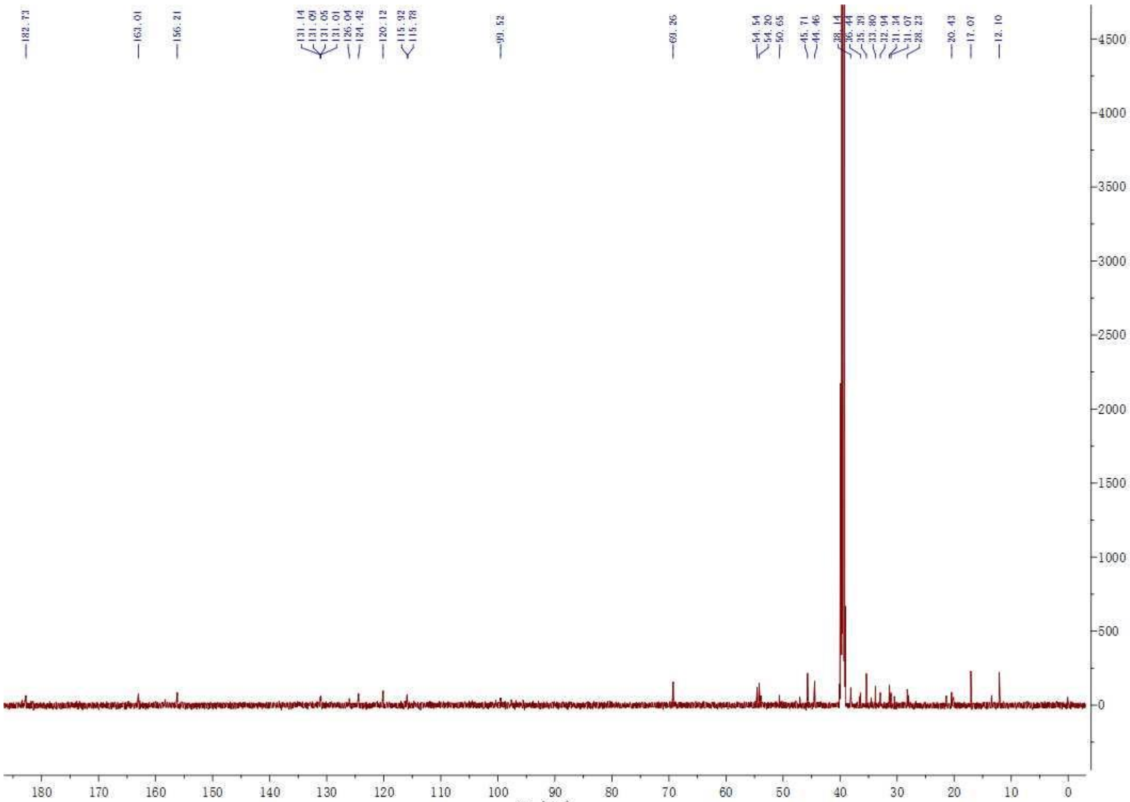

Figure S8: ^{13}C NMR spectrum for compound 3d.

Compd 3e

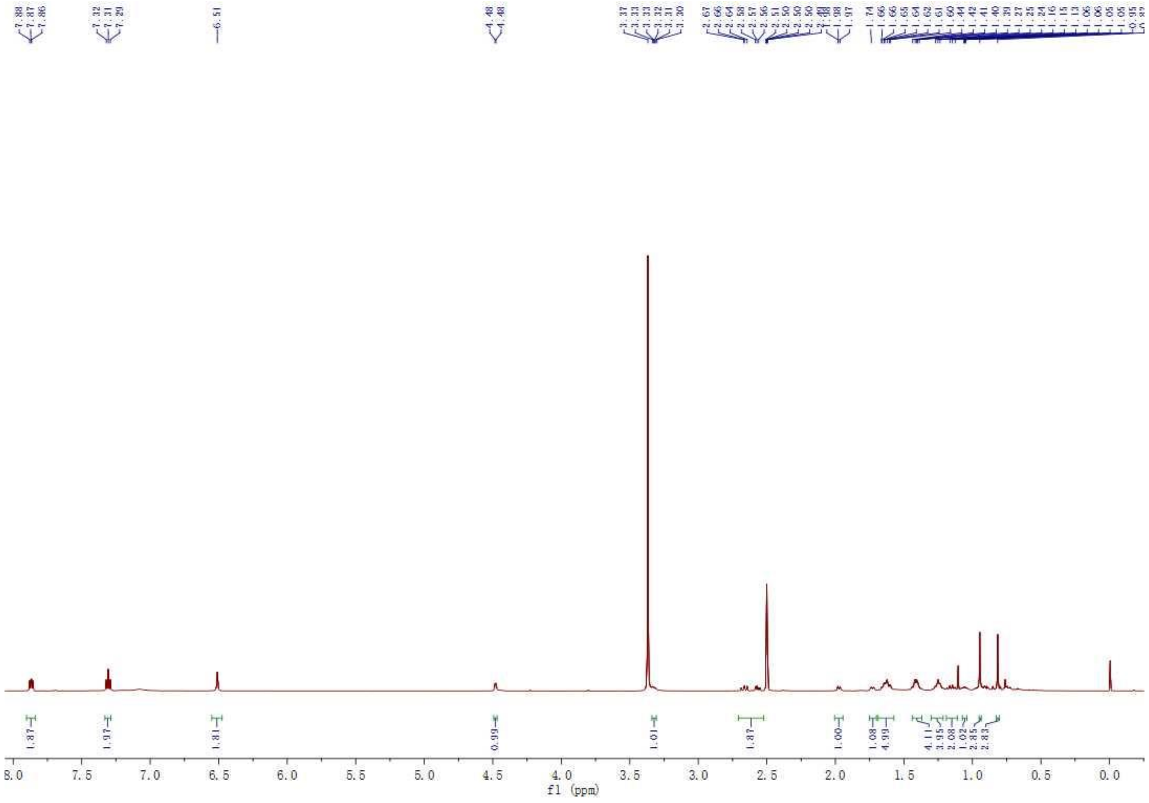

Figure S9: ^1H NMR spectrum for compound 3e.

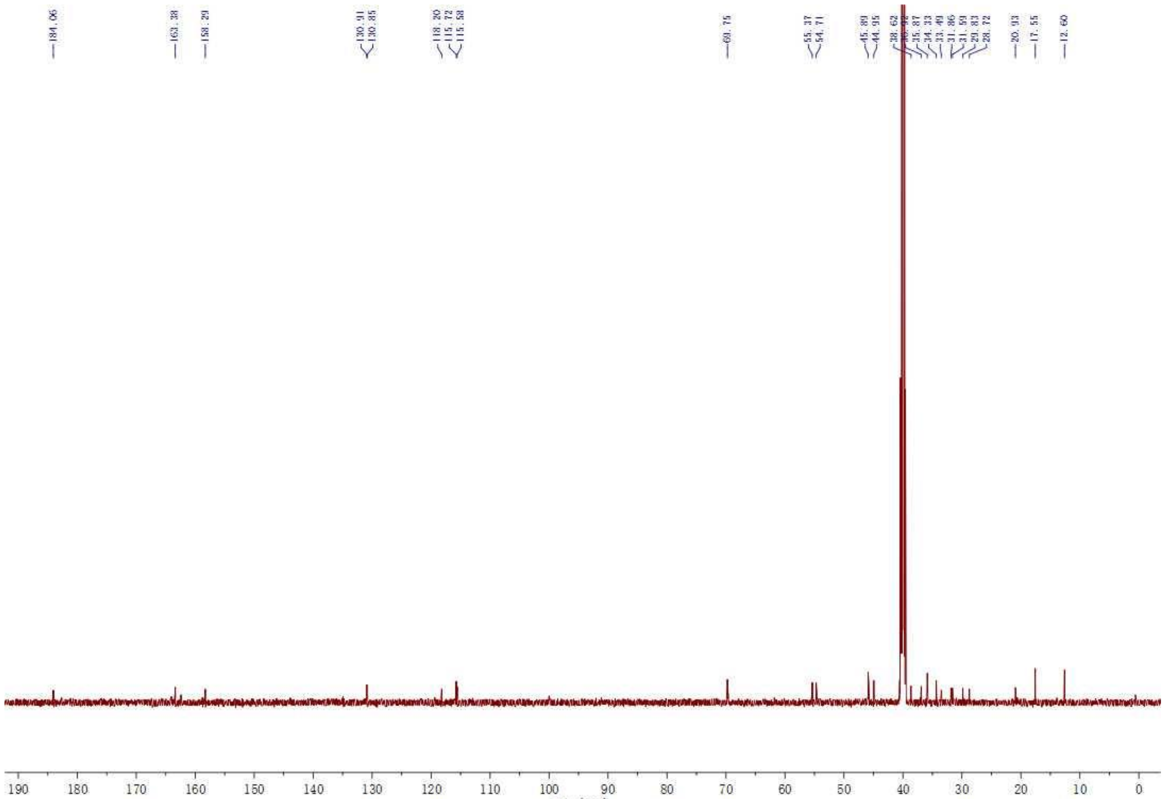

Figure S10: ^{13}C NMR spectrum for compound 3e.

Compd 3f

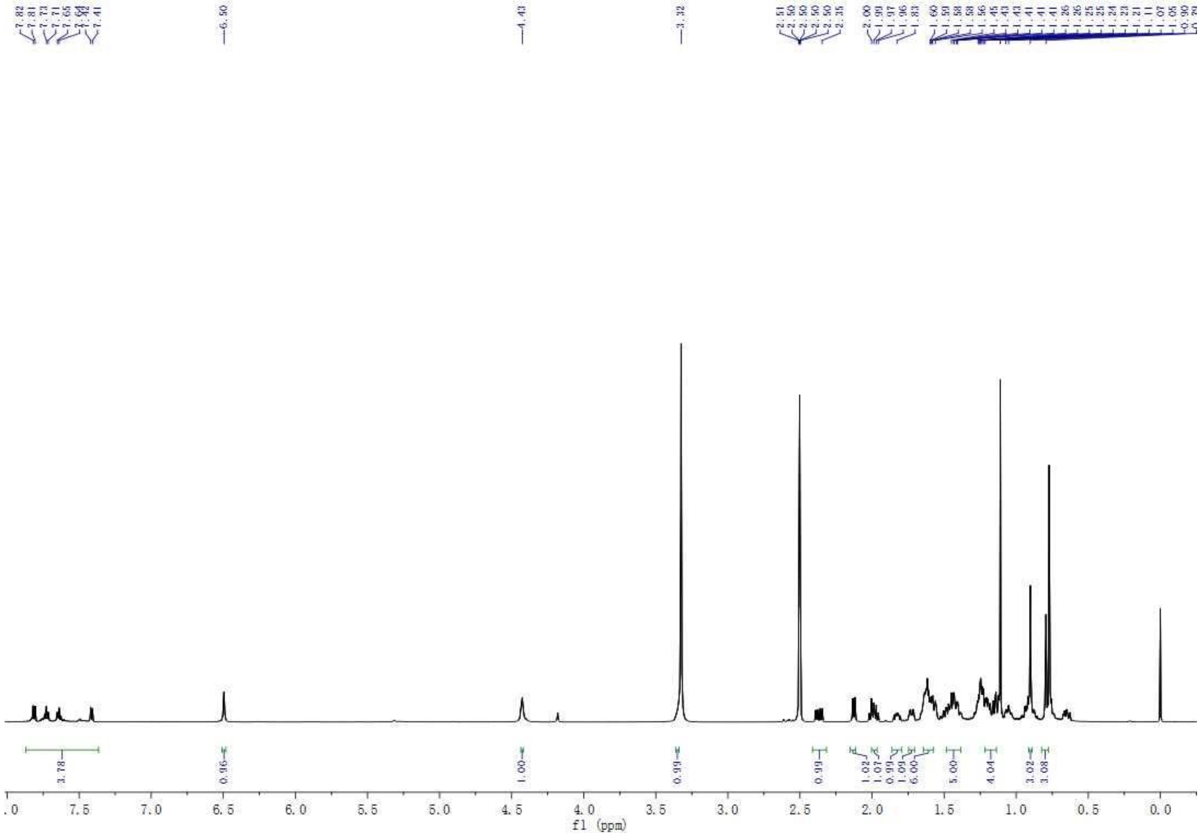

Figure S11: ^{1}H NMR spectrum for compound 3f.

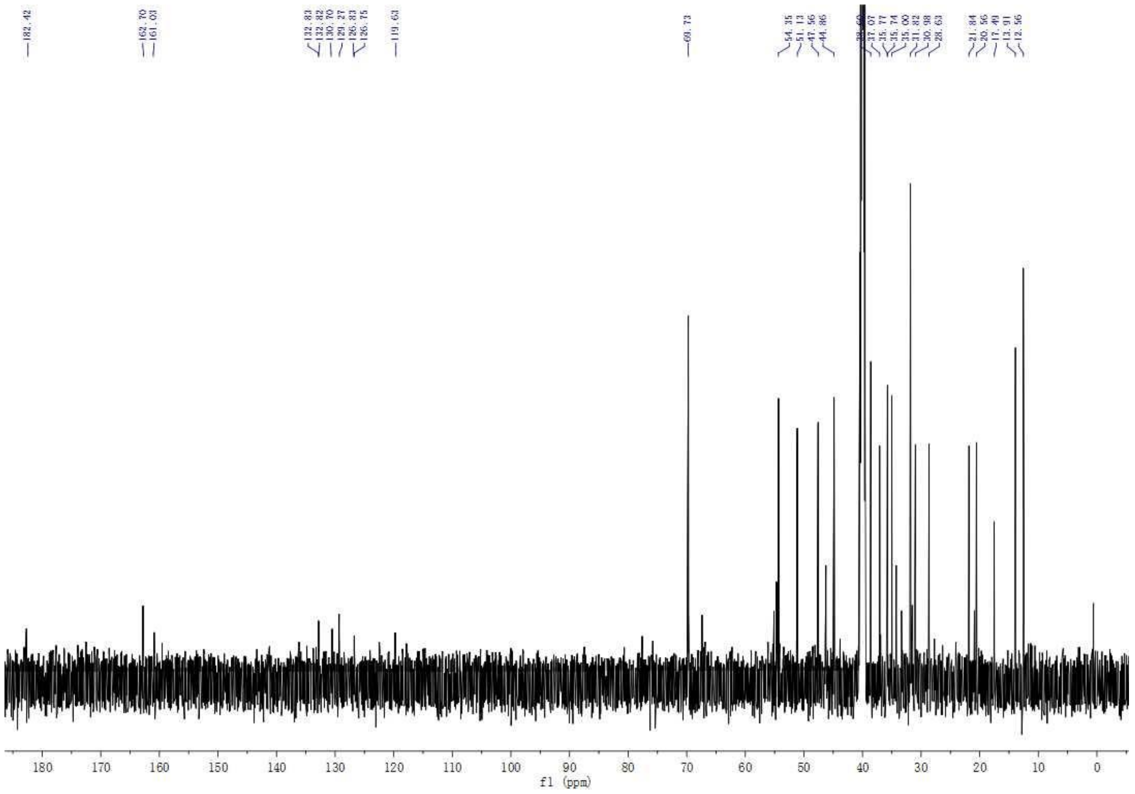

Figure S12: ^{13}C NMR spectrum for compound 3f.

Compd 3g

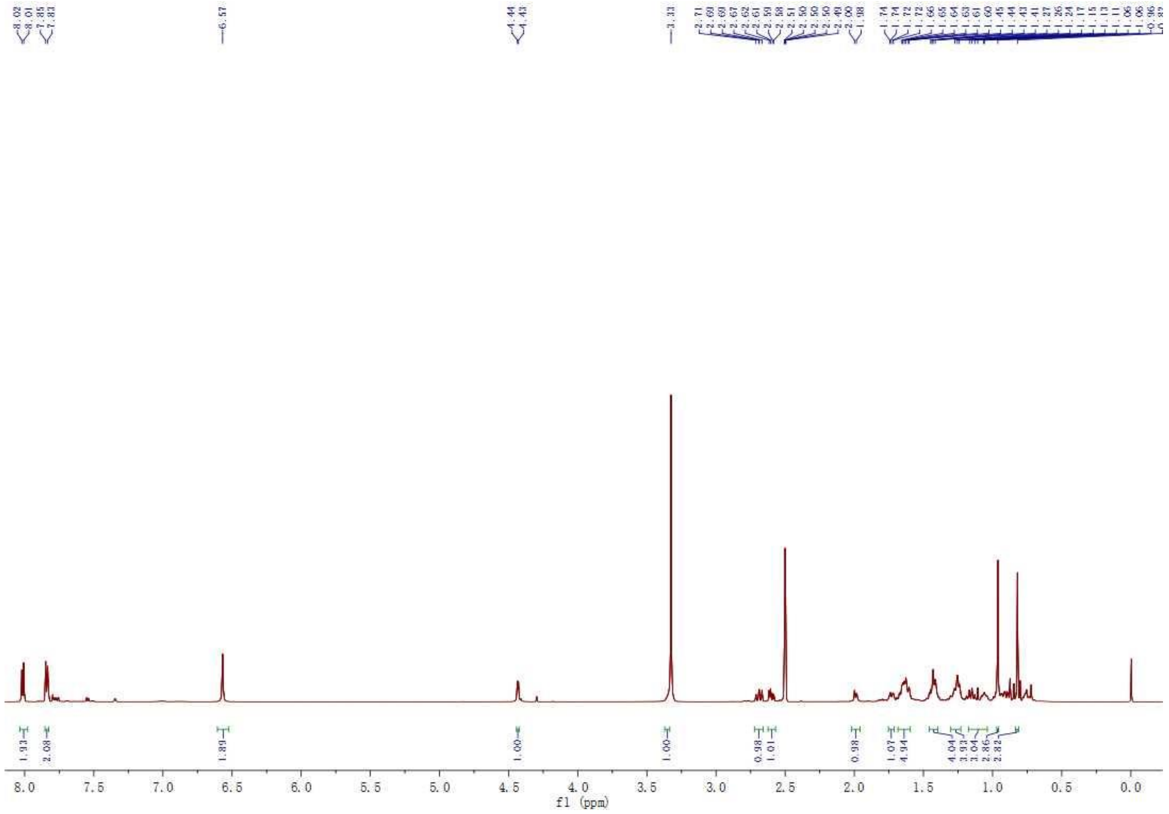

Figure S13: ^{1}H NMR spectrum for compound 3g.

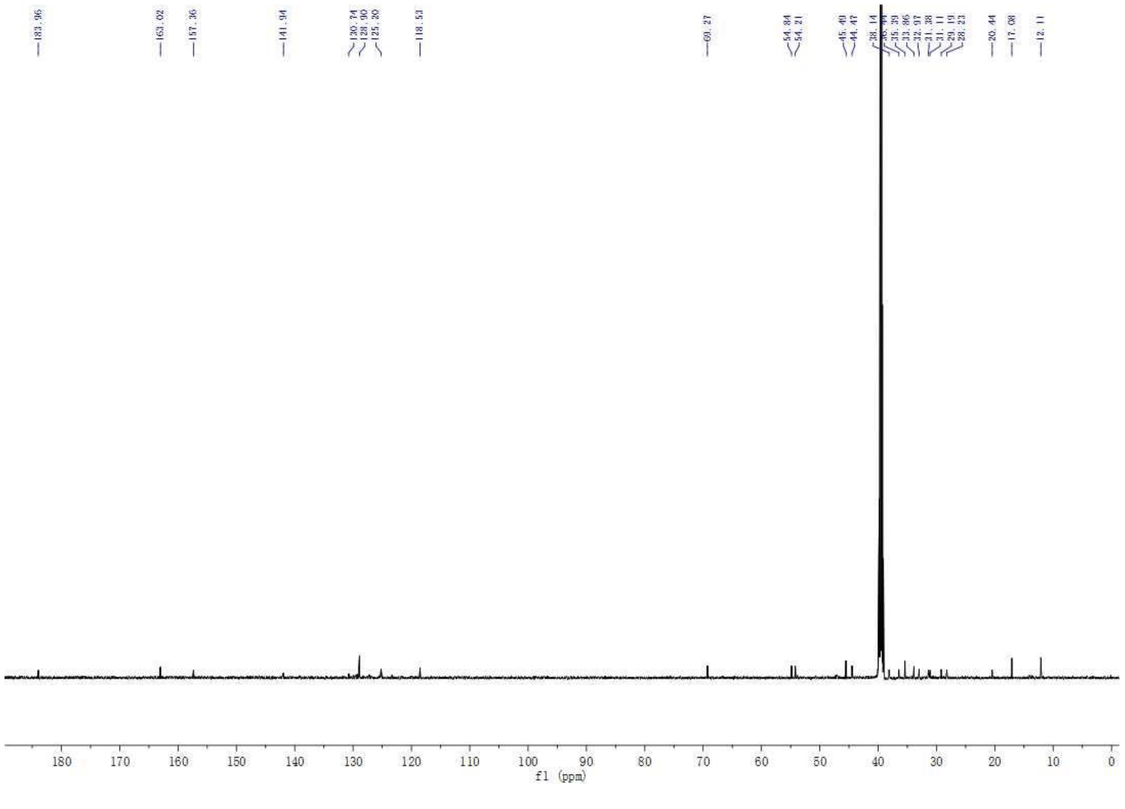

Figure S14: ^{13}C NMR spectrum for compound 3g.

Compd 3h

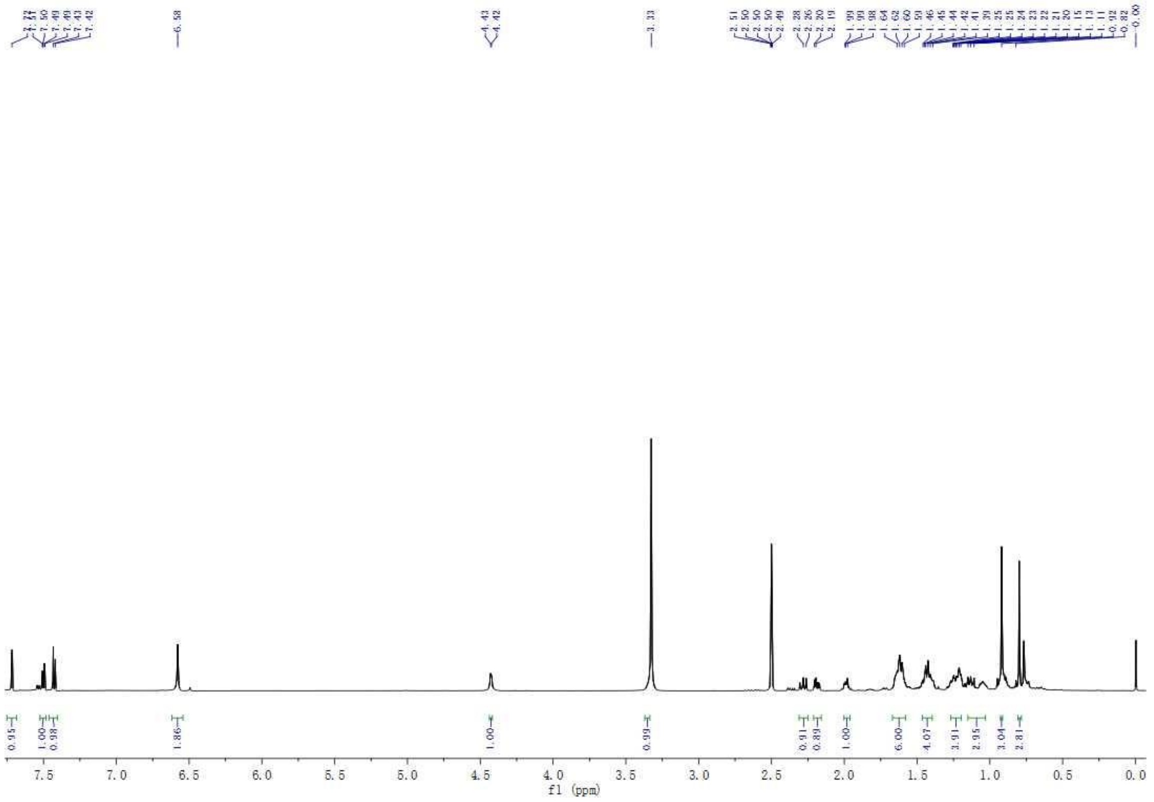

Figure S15: ^{1}H NMR spectrum for compound 3h.

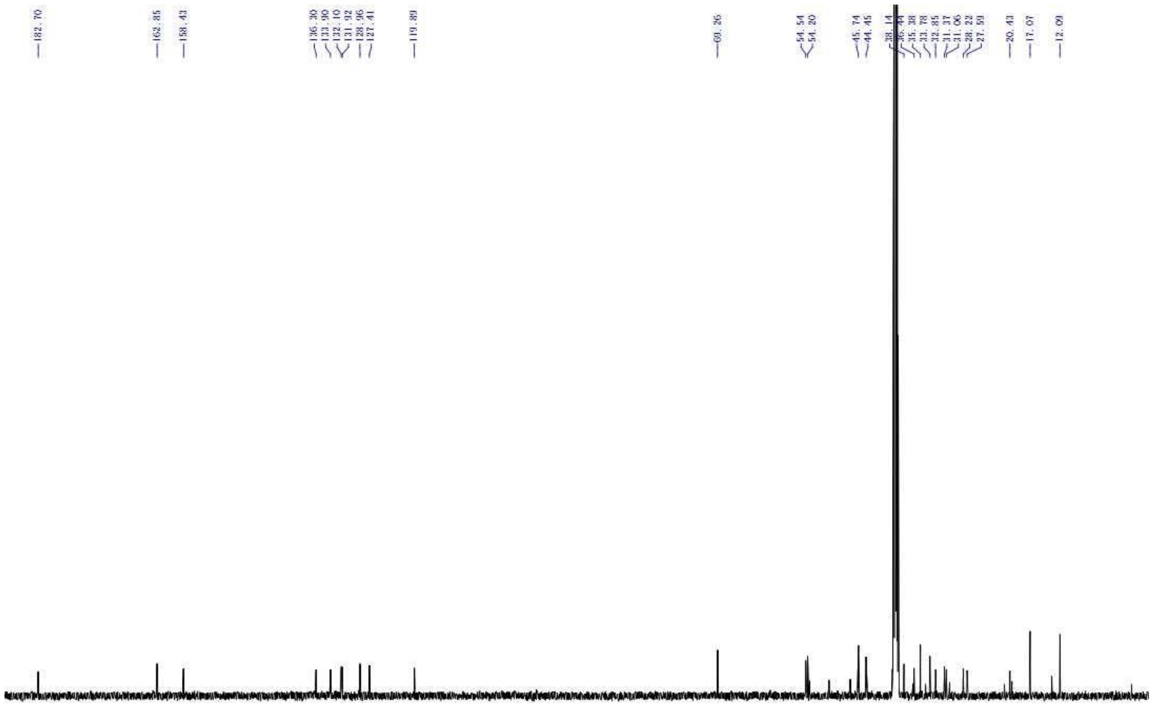

Figure S16: ^{13}C NMR spectrum for compound 3h.

Compd 3i

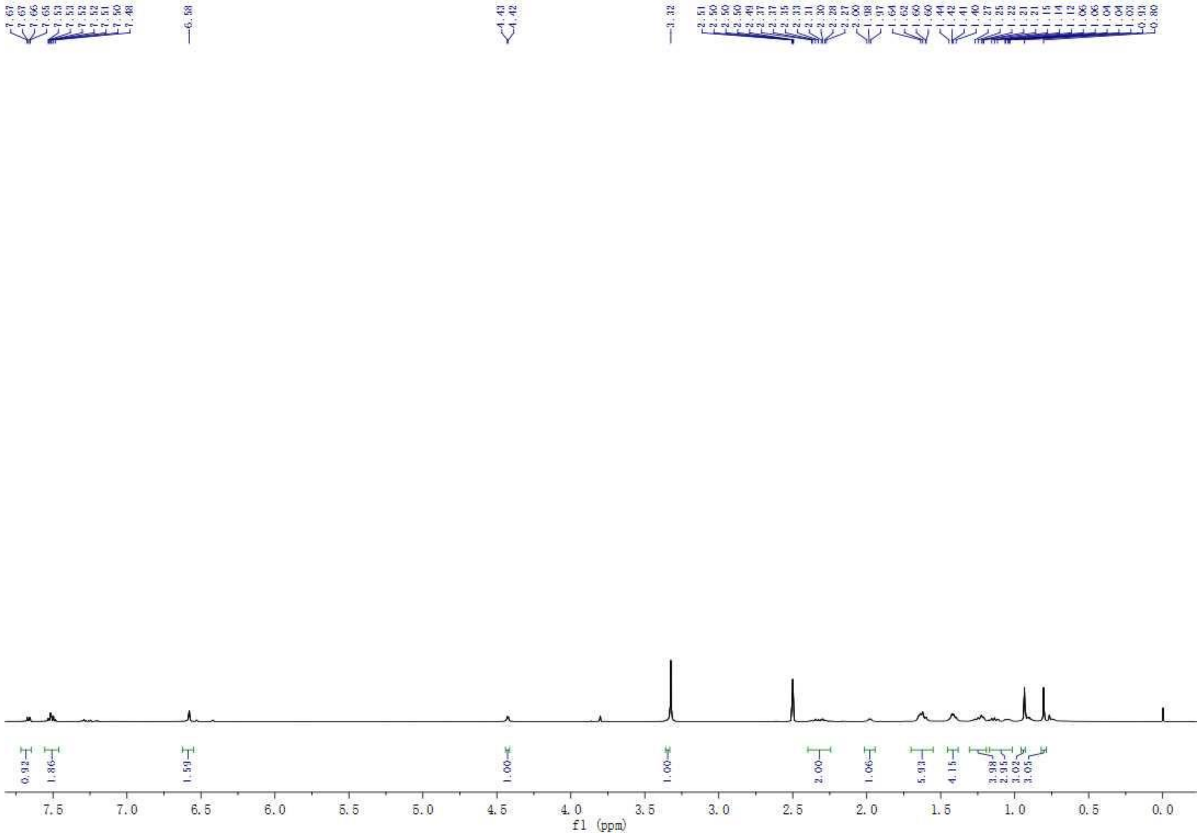

Figure S17: ^{1}H NMR spectrum for compound 3i.

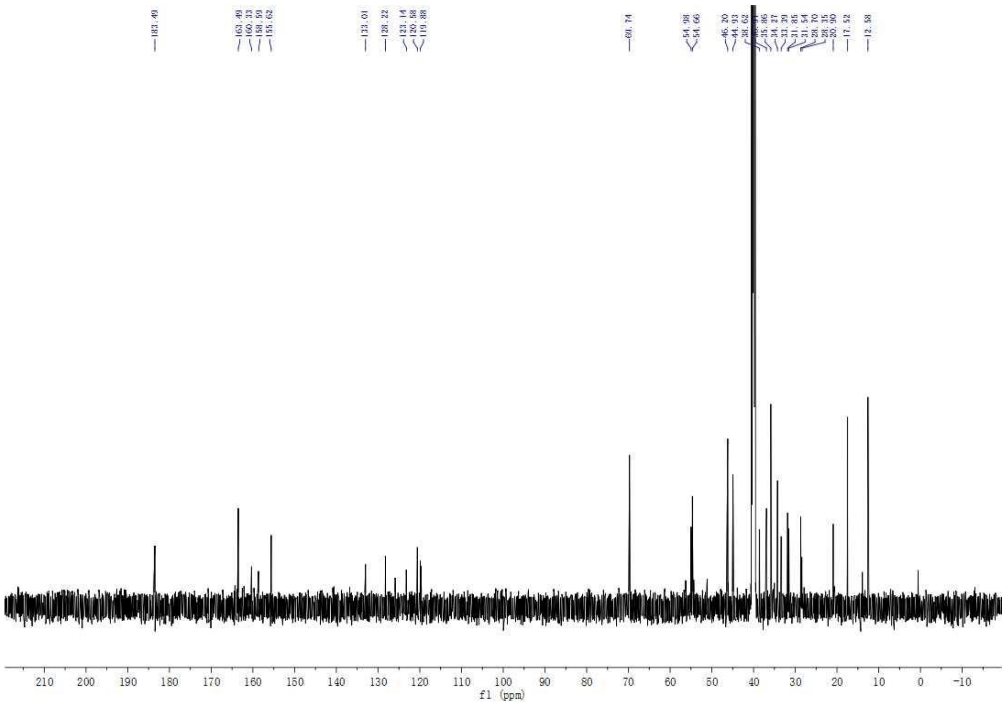

Figure S18: ^{13}C NMR spectrum for compound 3i.

Compd 3j

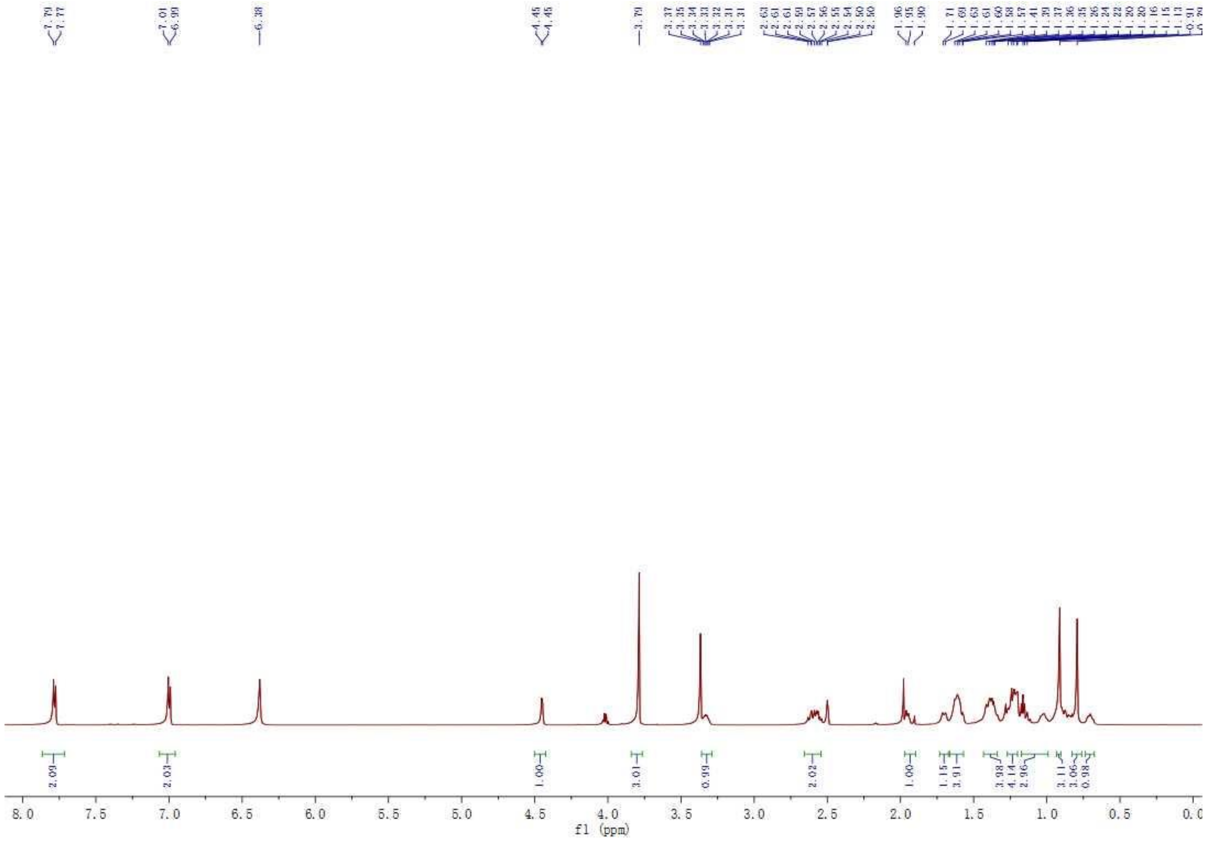

Figure S19: ^{1}H NMR spectrum for compound 3j.

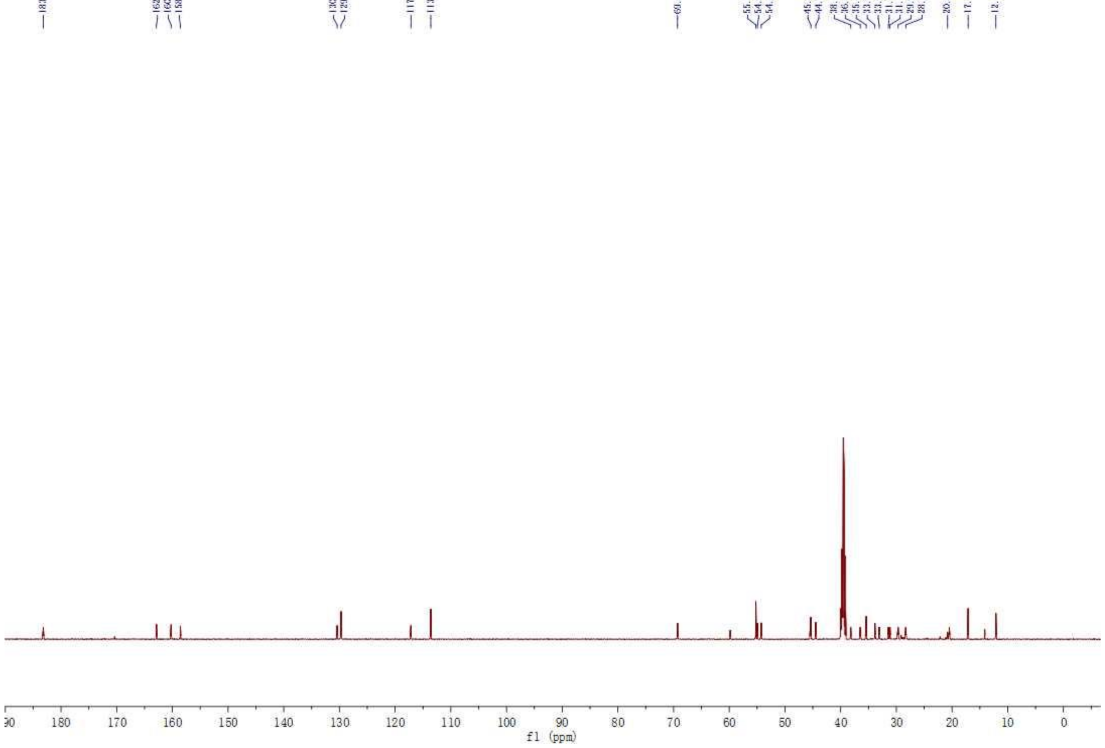

Figure S20: ^{13}C NMR spectrum for compound 3j.

Compd 3k

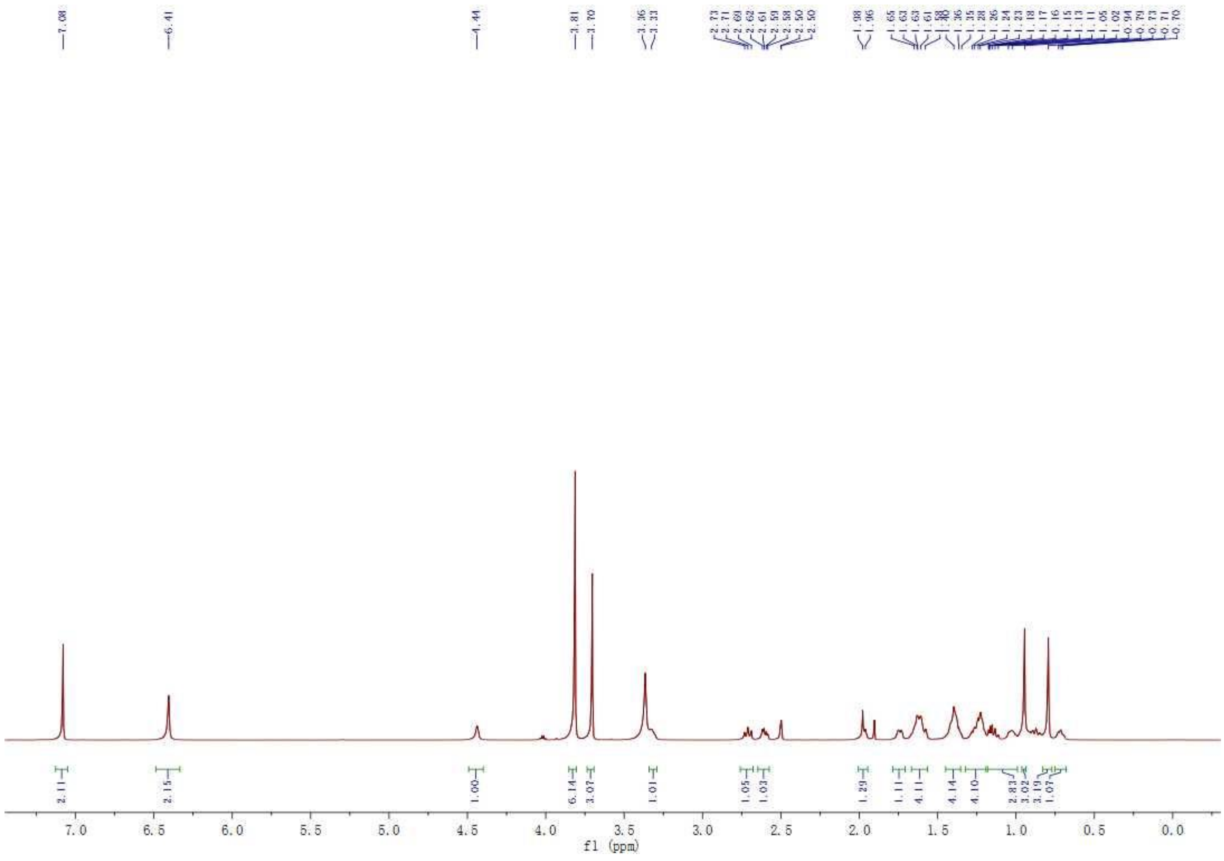

Figure S21: ^{1}H NMR spectrum for compound 3k.

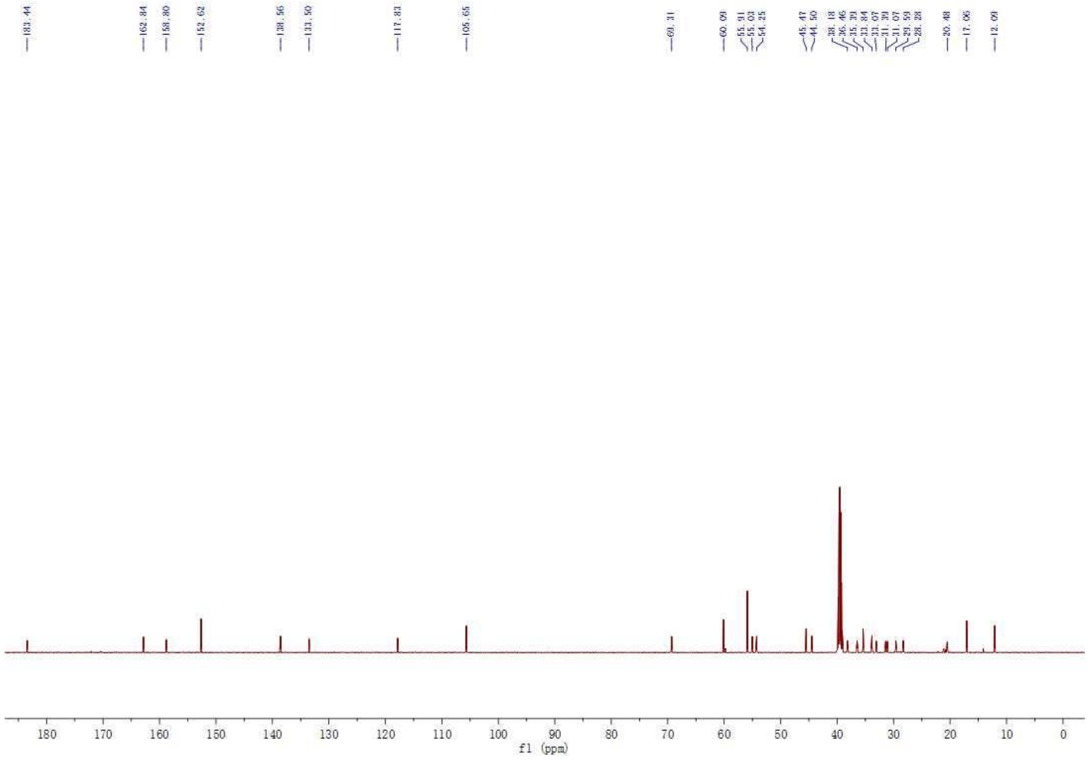

Figure S22: ^{13}C NMR spectrum for compound 3k.

Compd 3l

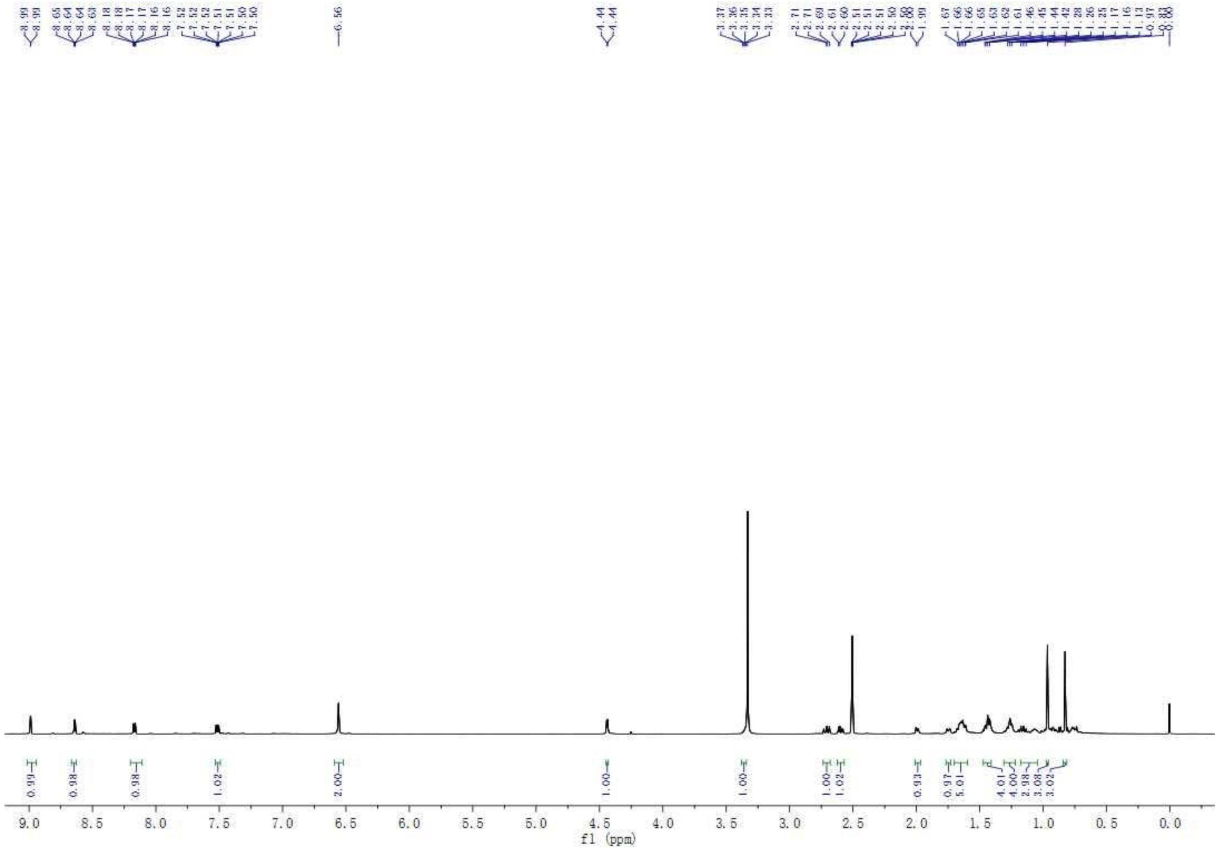

Figure S23: ^{1}H NMR spectrum for compound 3l.

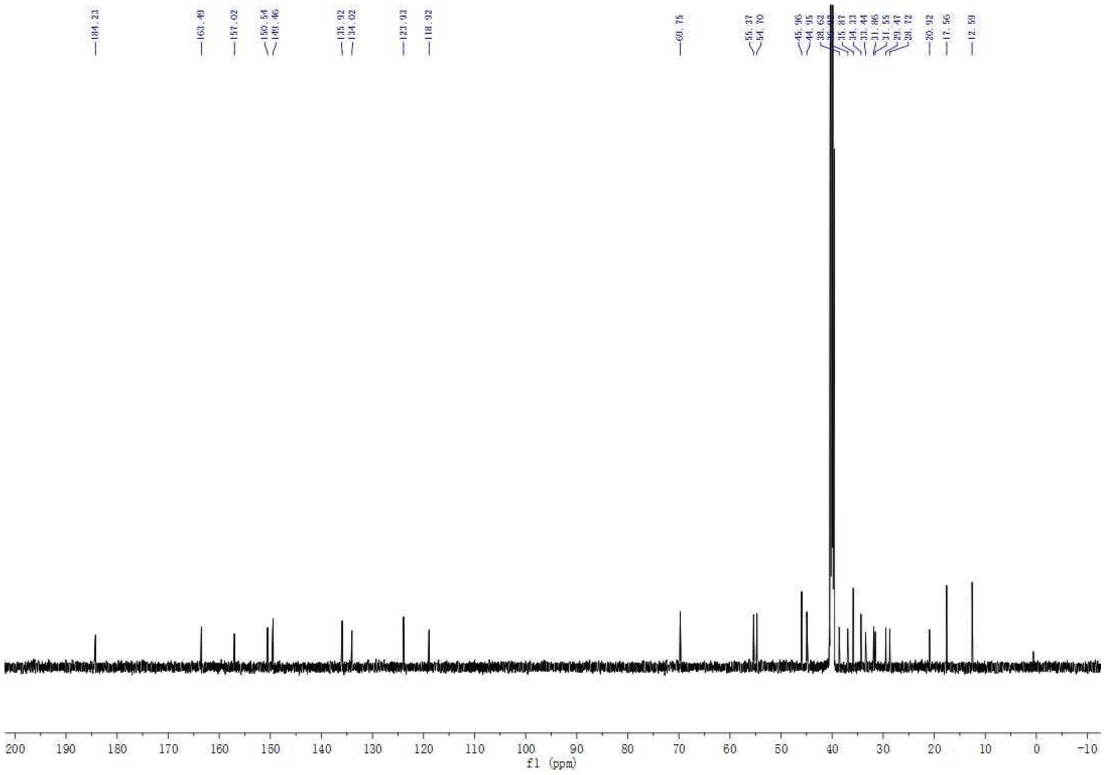

Figure S24: ^{13}C NMR spectrum for compound 3l.

Compd 6a

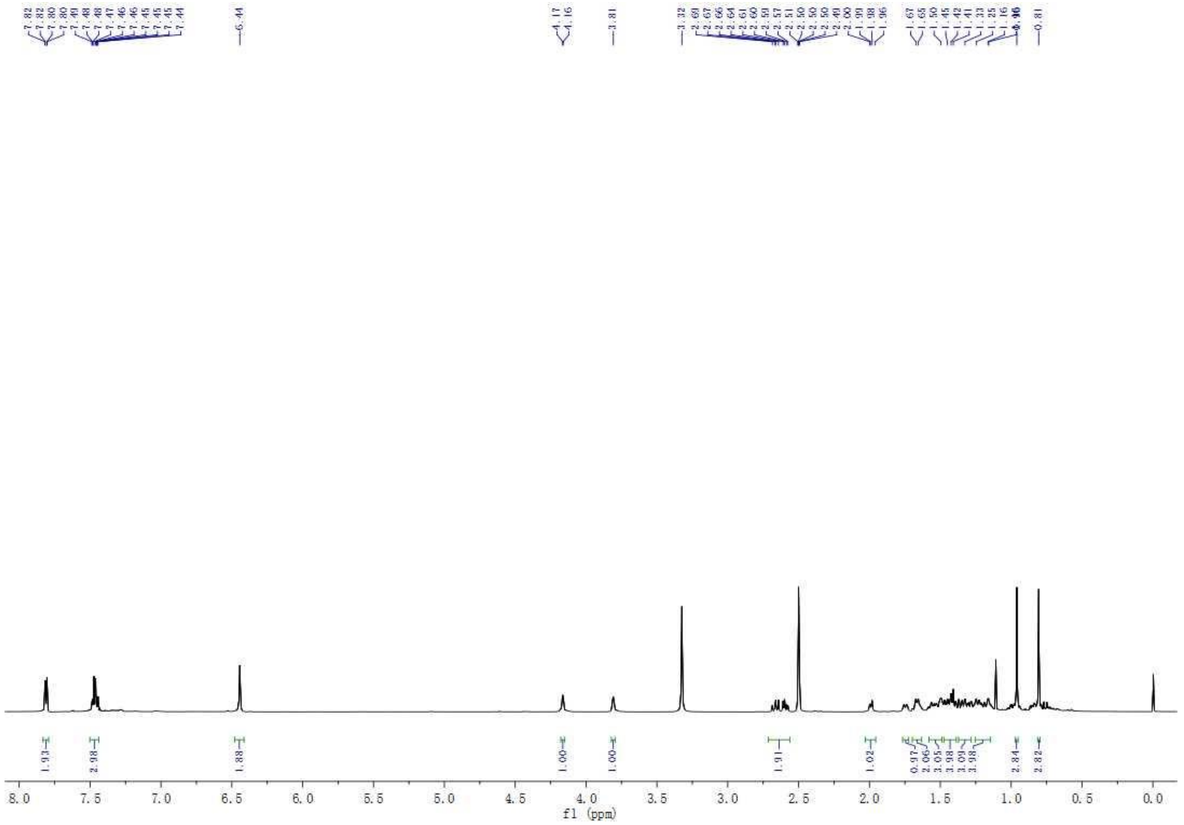

Figure S25: ^1H NMR spectrum for compound 6a.

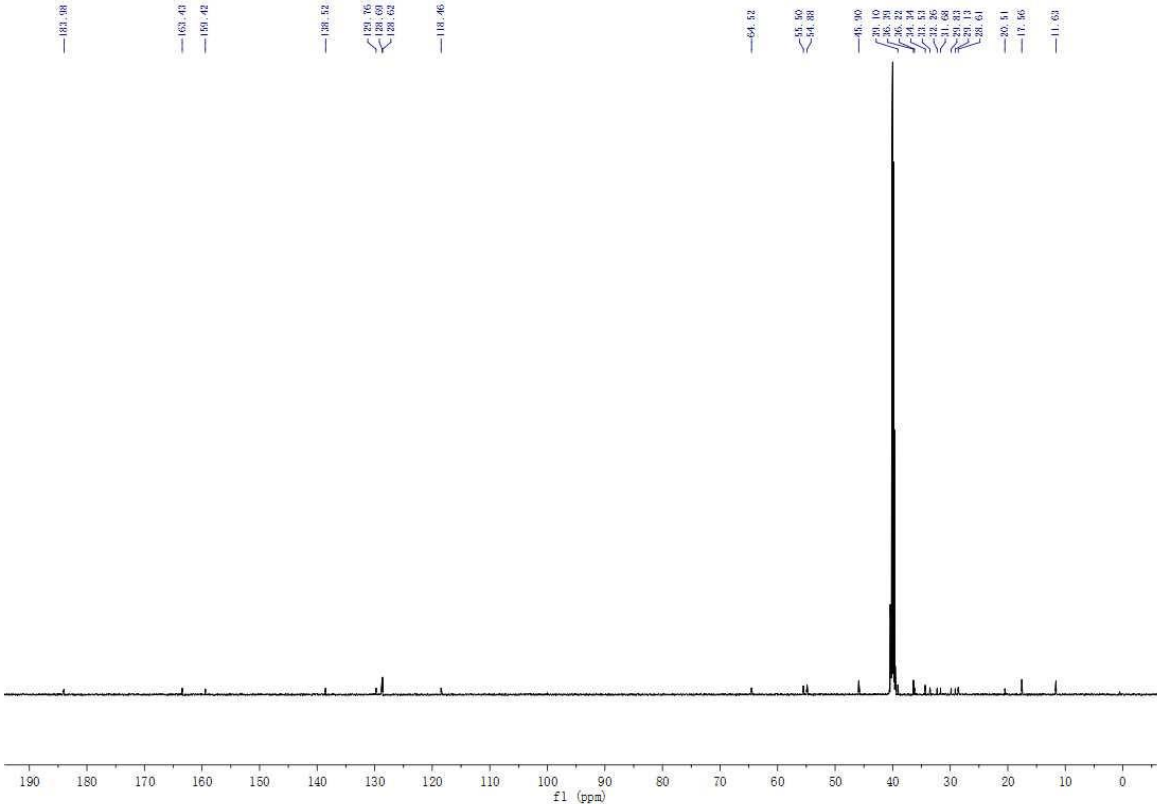

Figure S26: ^{13}C NMR spectrum for compound 6a.

Compd 6b

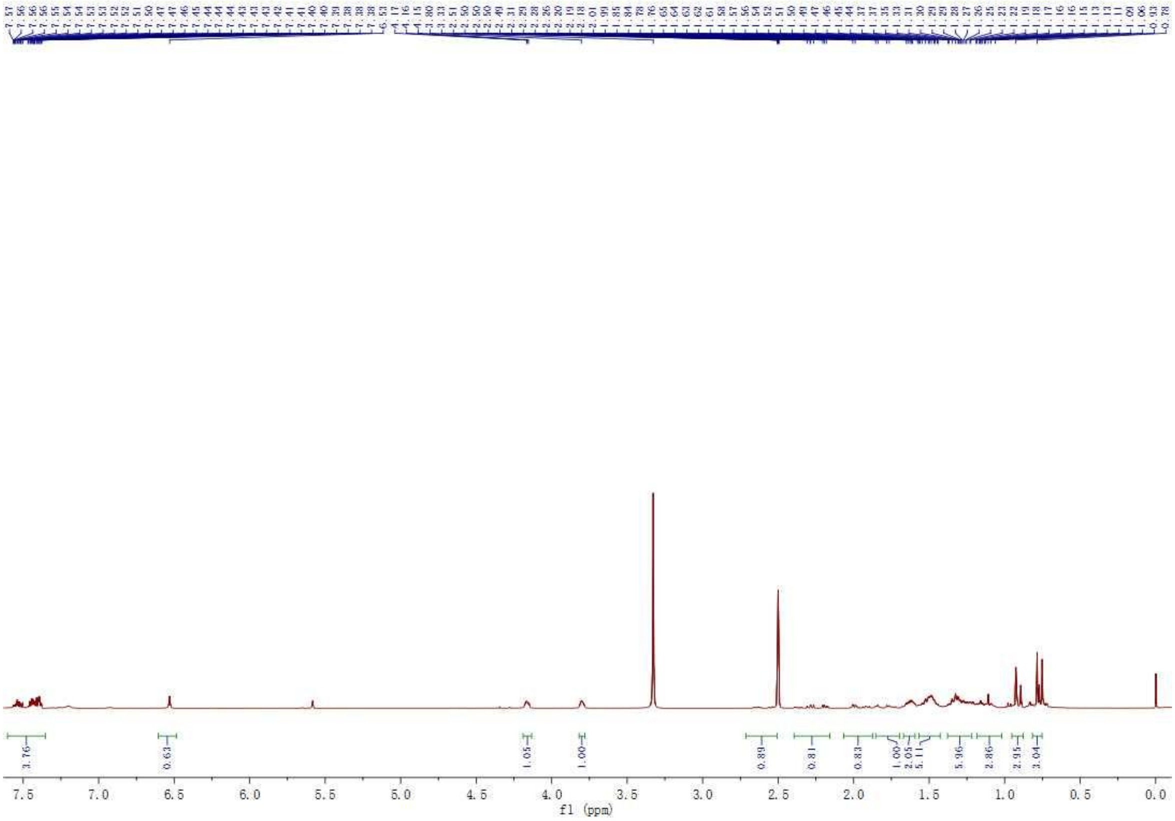

Figure S27: ^1H NMR spectrum for compound 6b.

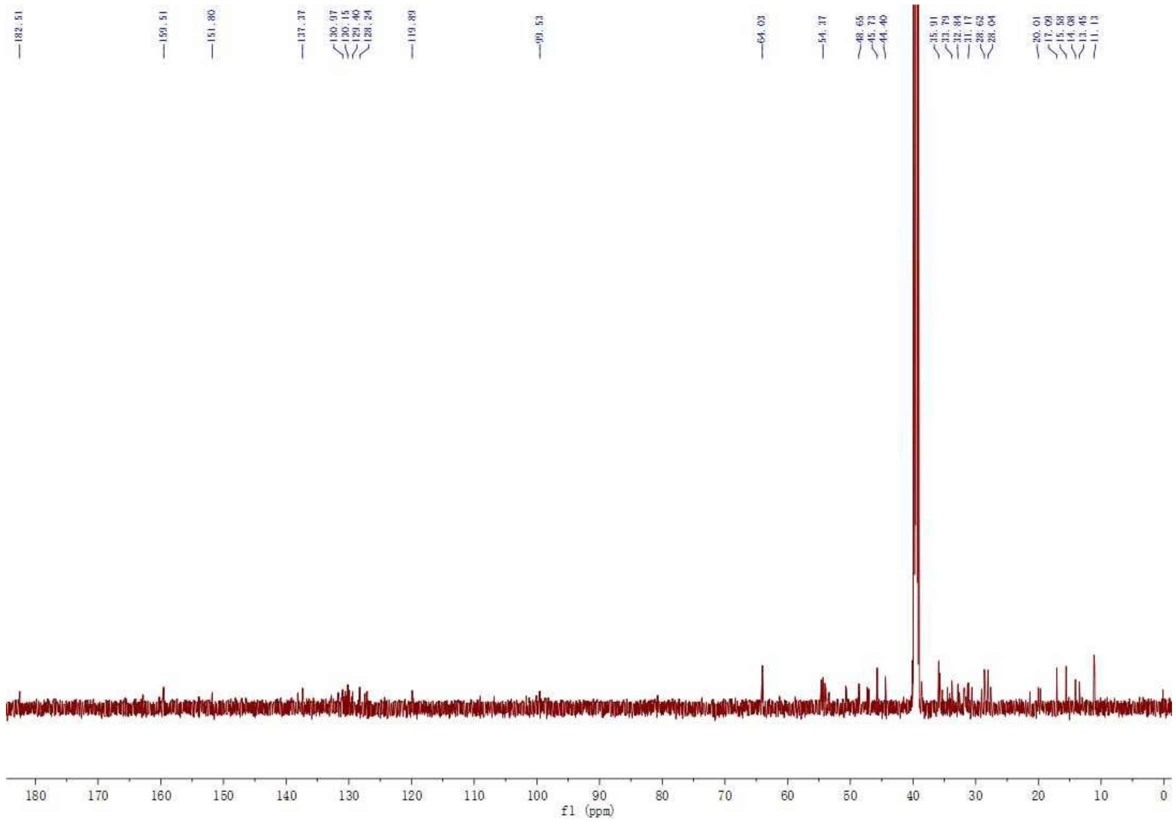

Figure S28: ^{13}C NMR spectrum for compound 6b.

Compd 6c

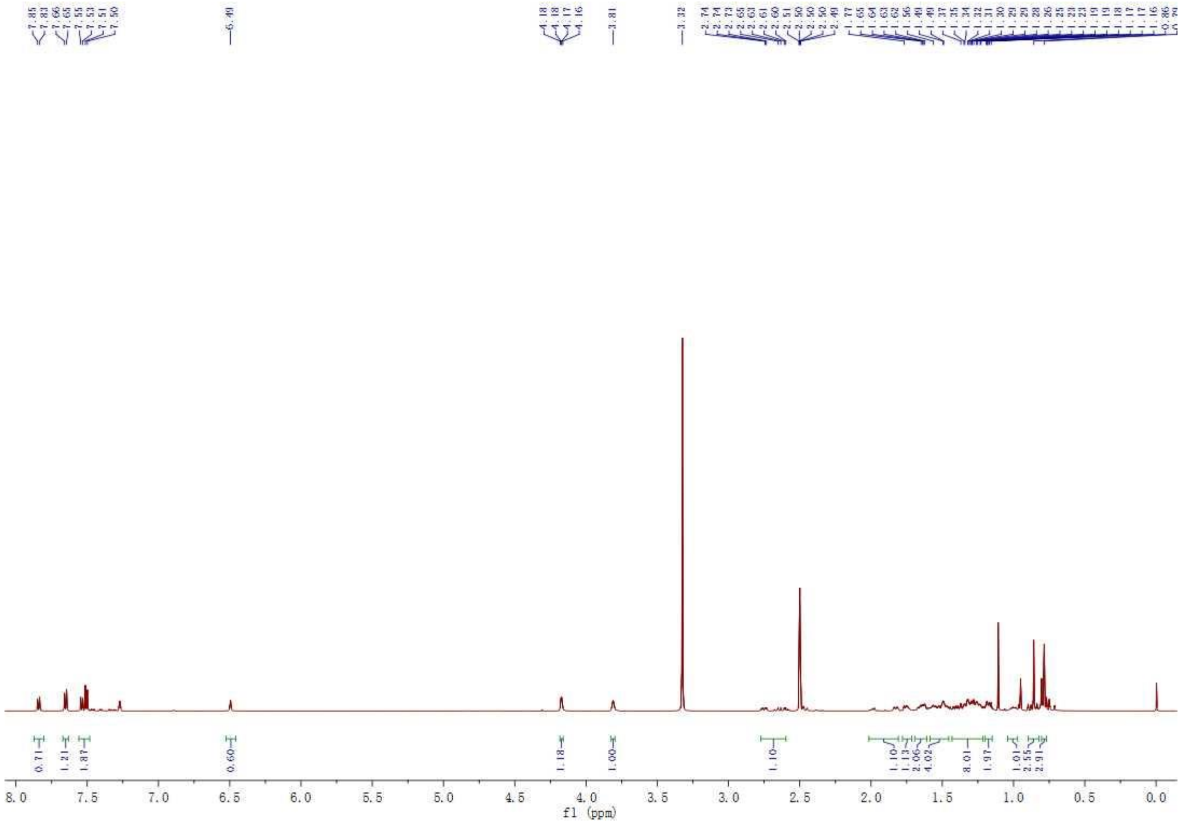

Figure S29: ^{1}H NMR spectrum for compound 6c.

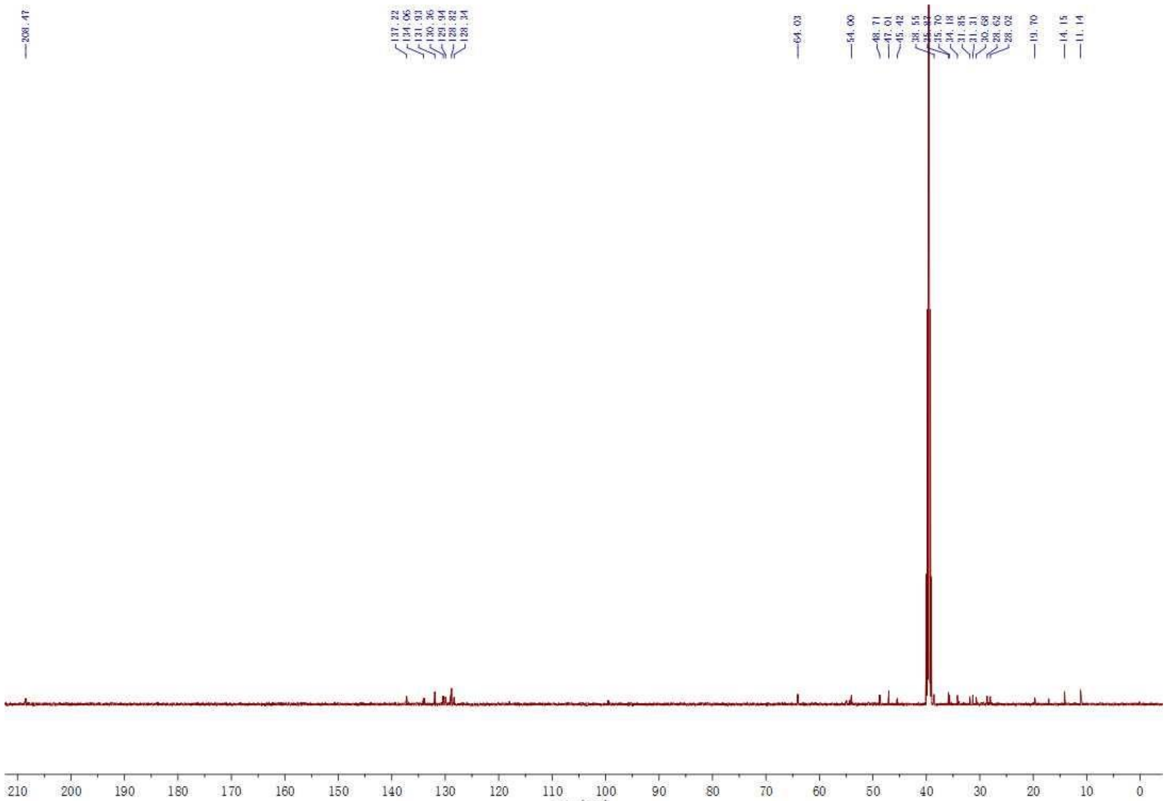

Figure S30: ^{13}C NMR spectrum for compound 6c.

Compd 6d

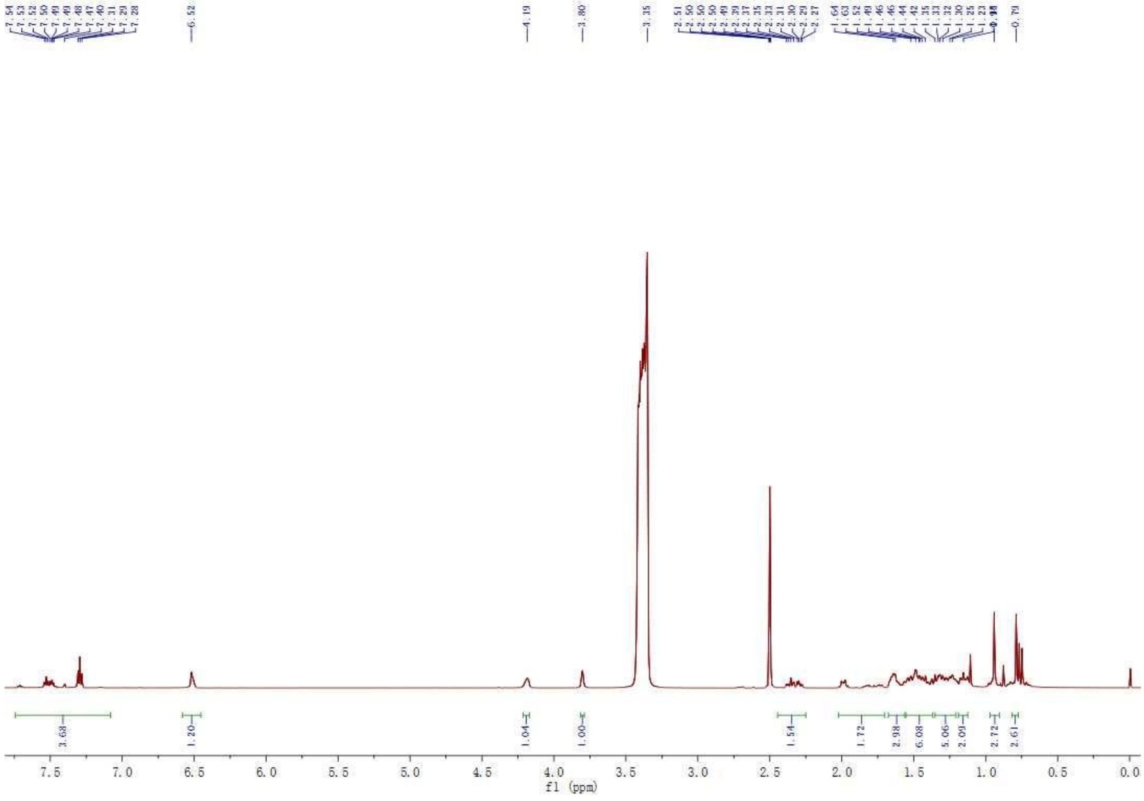

Figure S31: ^{1}H NMR spectrum for compound 6d.

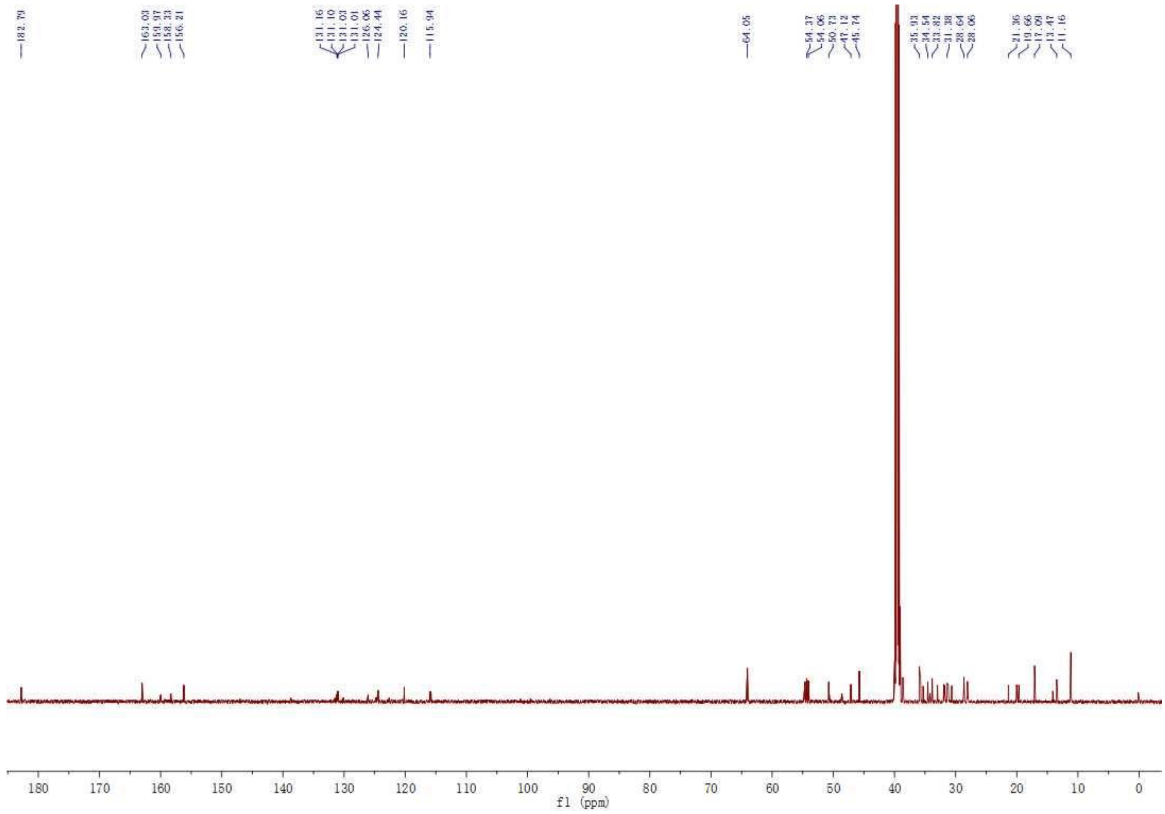

Figure S32: ^{13}C NMR spectrum for compound 6d.

Compd 6e

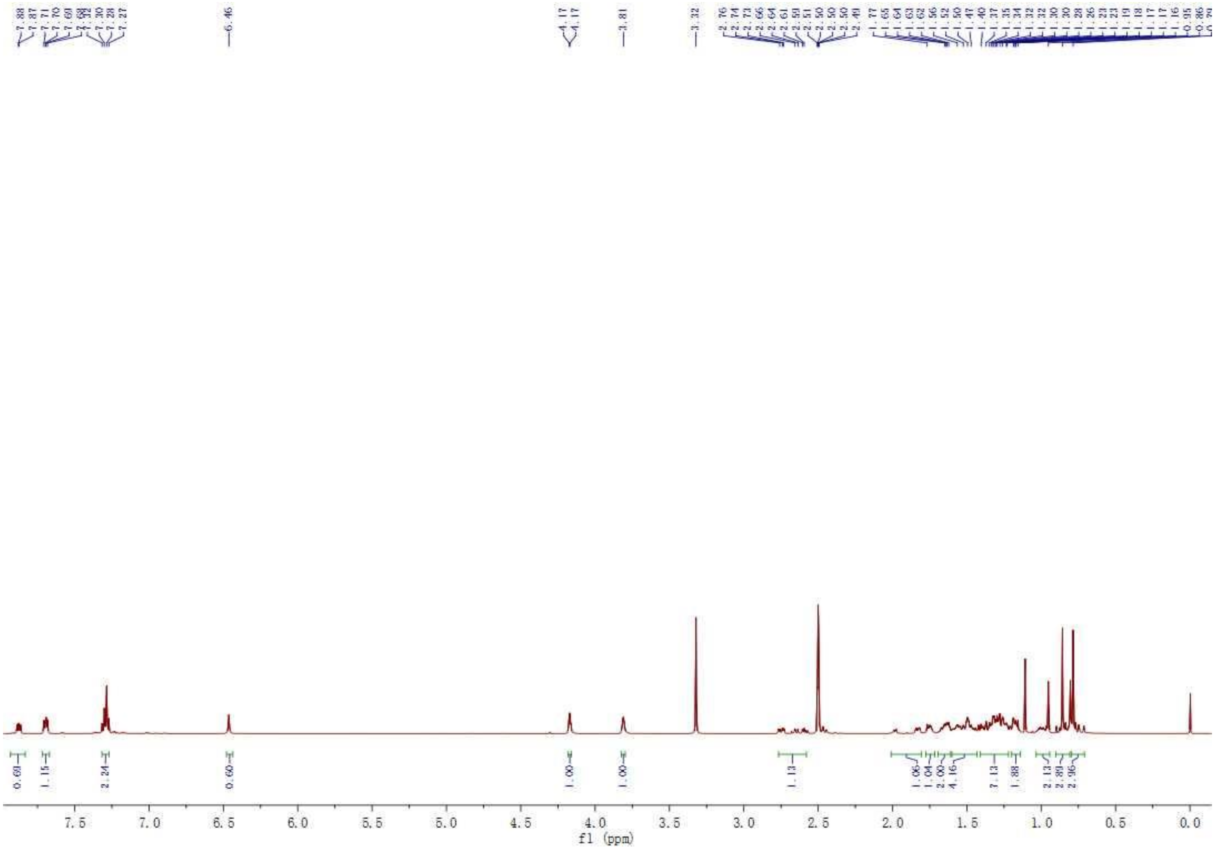

Figure S33: ^{1}H NMR spectrum for compound 6e.

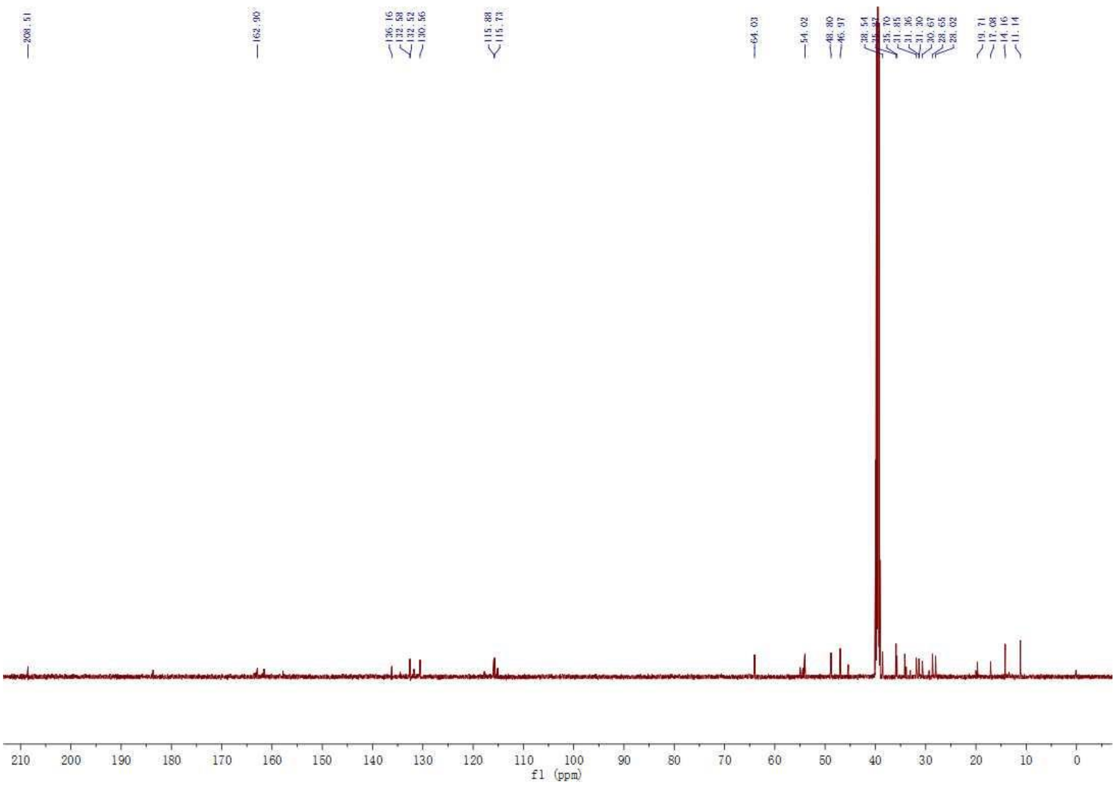

Figure S34: ^{13}C NMR spectrum for compound 6e.

Compd 6f

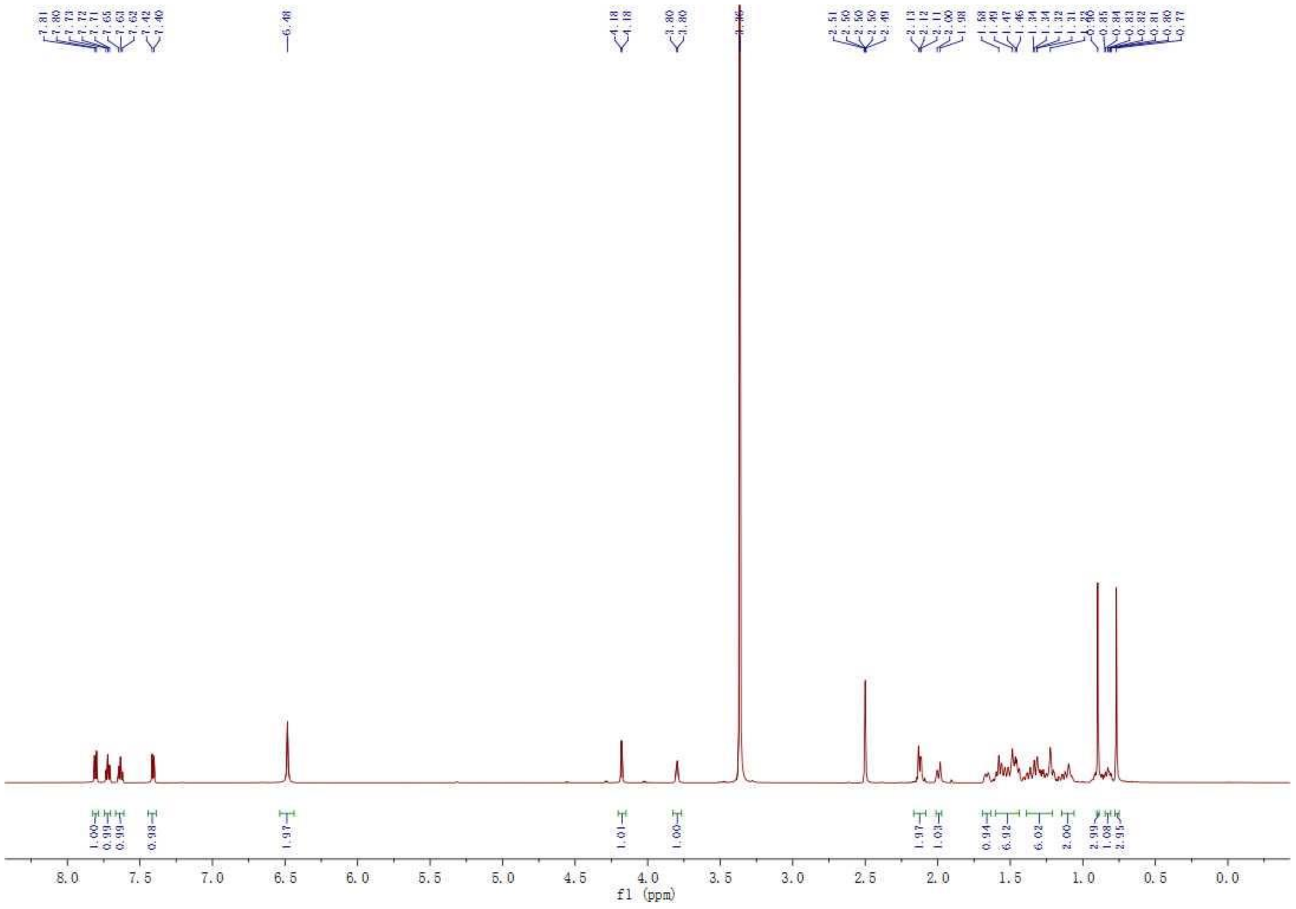

Figure S35: ^{1}H NMR spectrum for compound 6f.

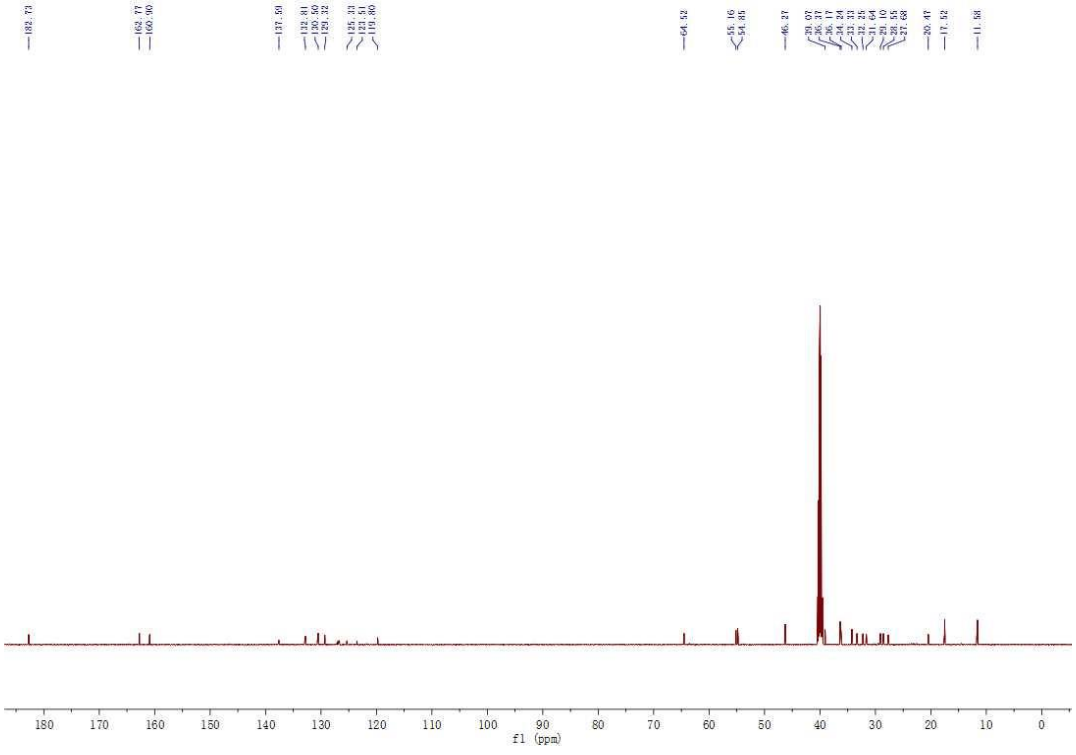

Figure S36: ^{13}C NMR spectrum for compound 6f.

Compd 6g

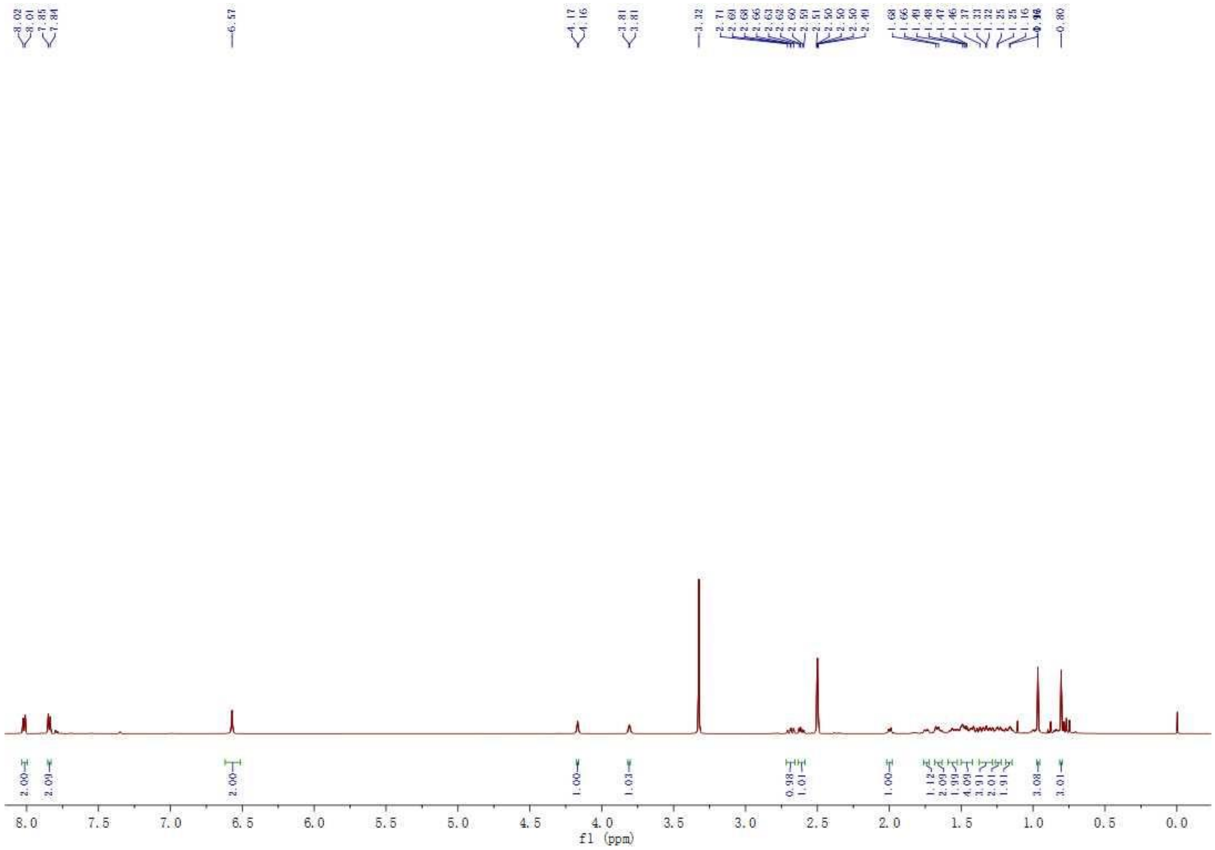

Figure S37: ^{1}H NMR spectrum for compound 6g.

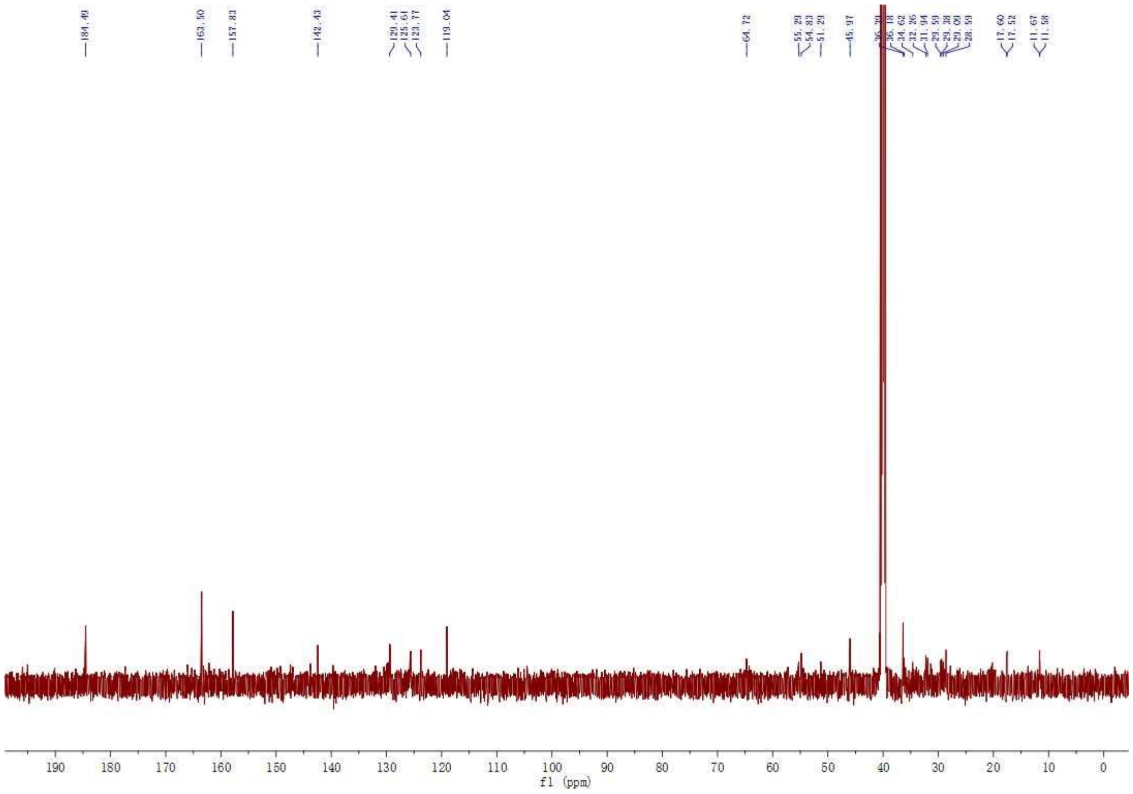

Figure S38: ^{13}C NMR spectrum for compound 6g.

Compd 6h

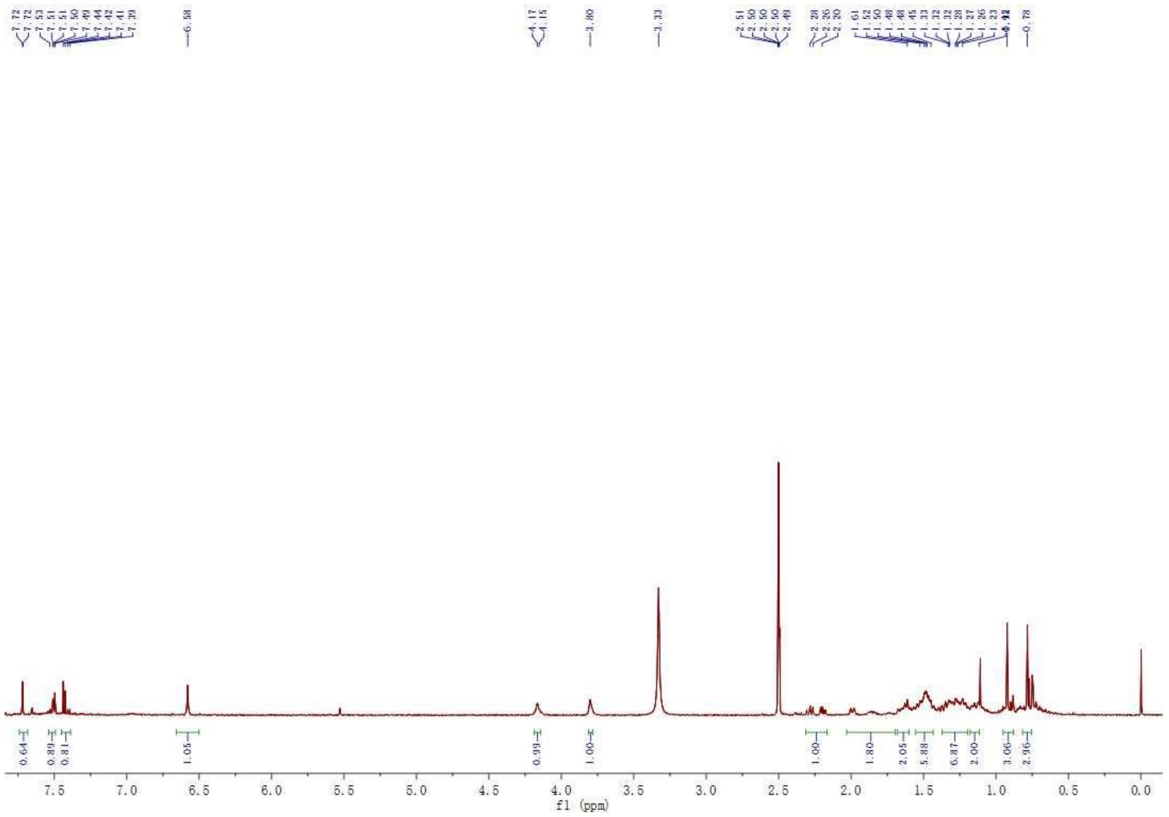

Figure S39: ^{1}H NMR spectrum for compound 6h.

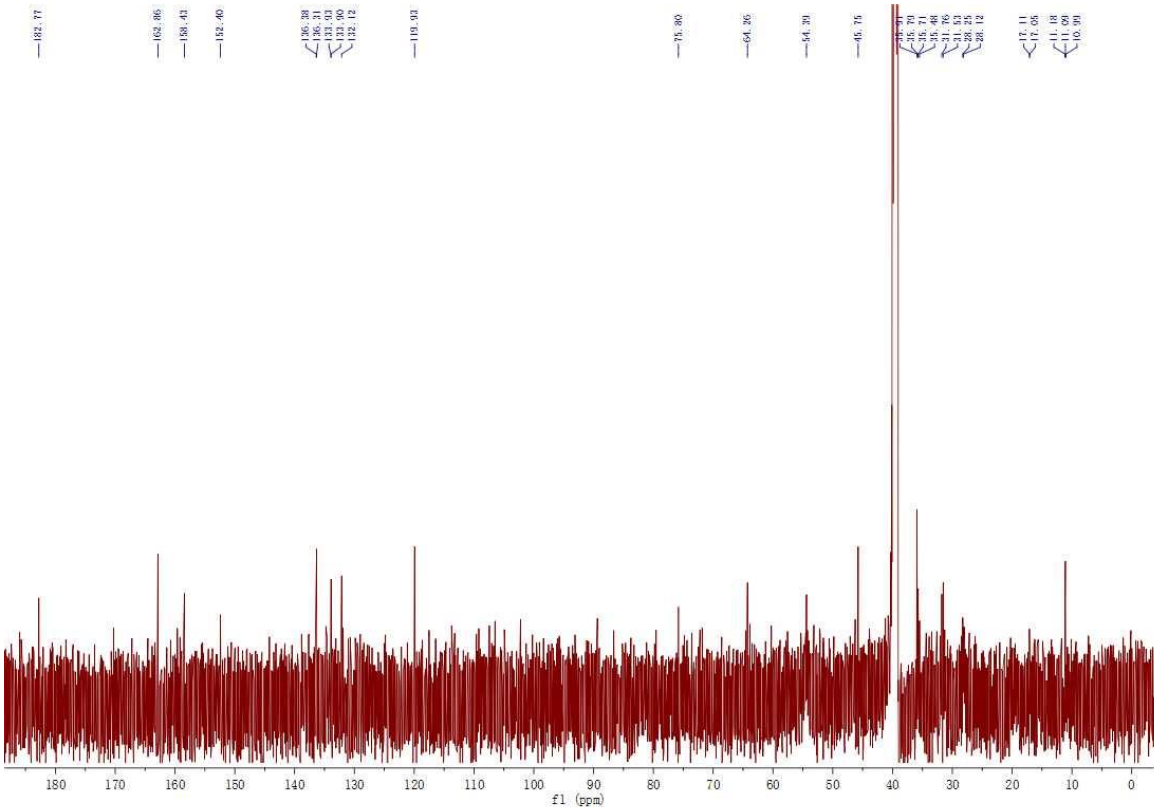

Figure S40: ^{13}C NMR spectrum for compound 6h.

Compd 6i

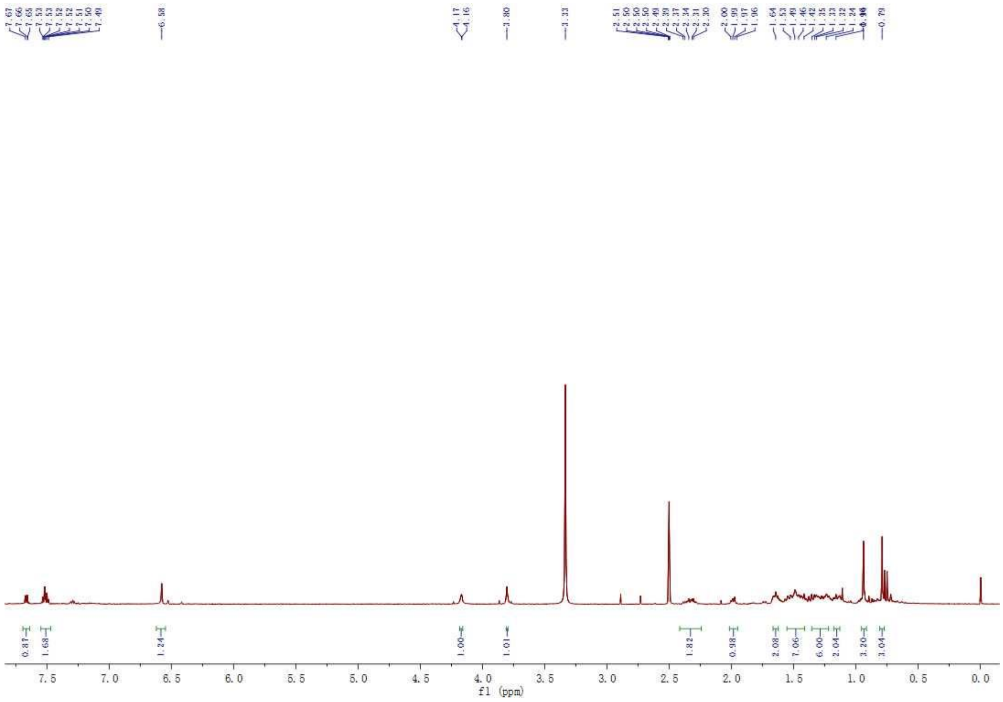

Figure S41: ^{1}H NMR spectrum for compound 6i.

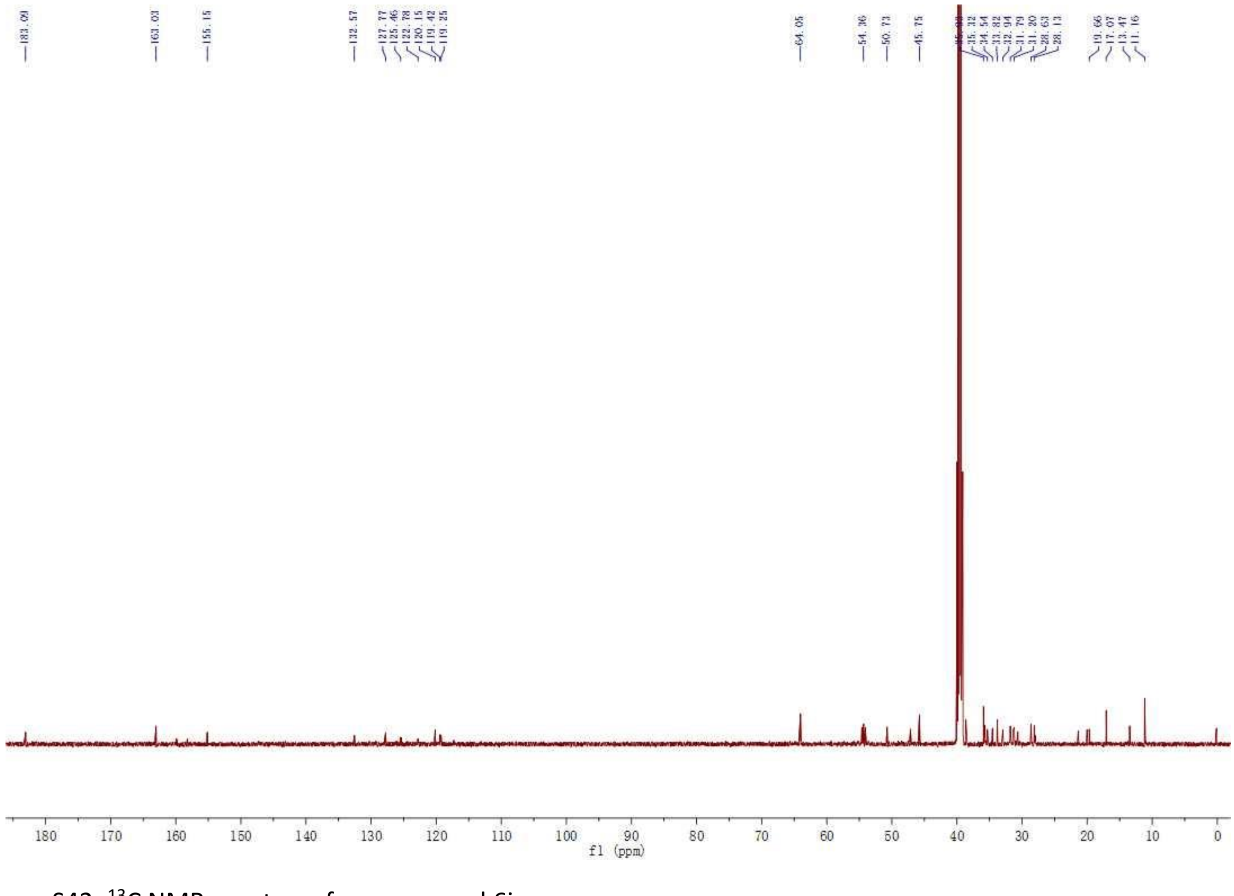

Figure S42: ^{13}C NMR spectrum for compound 6i.

Compd 6j

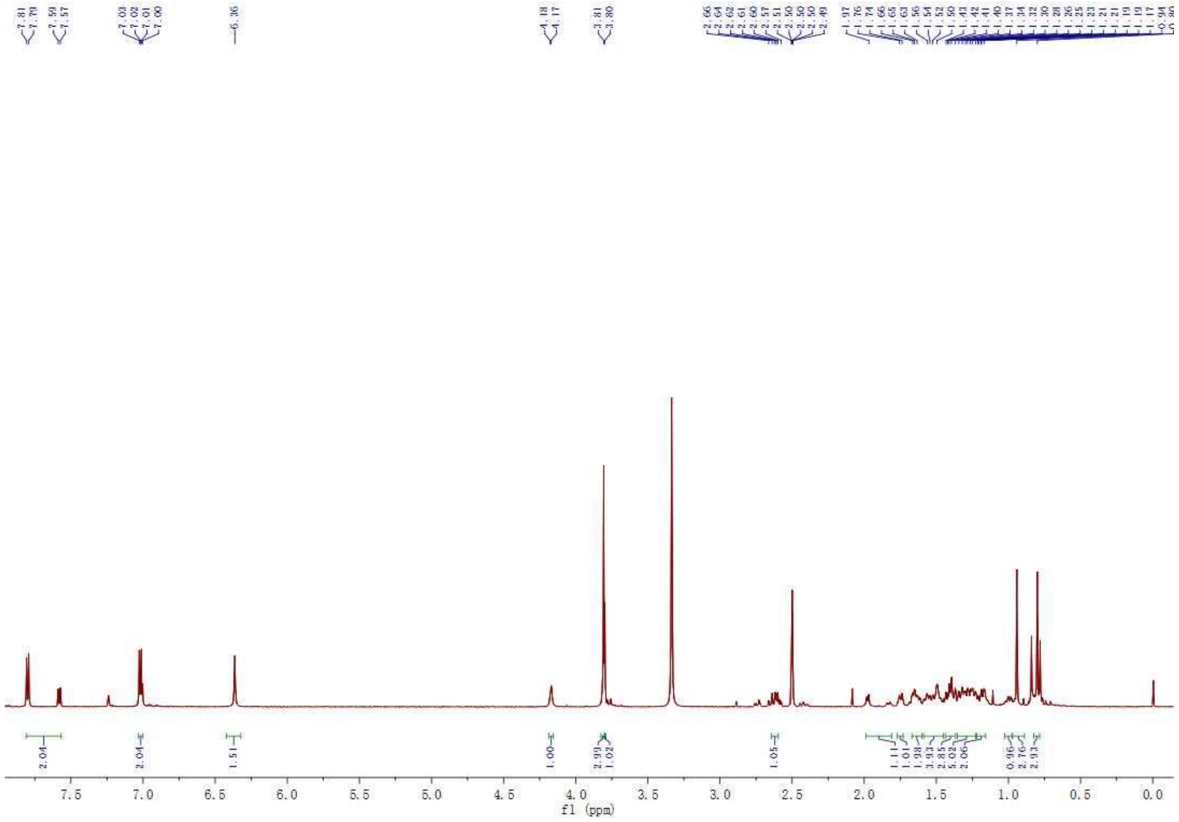

Figure S43: ^{1}H NMR spectrum for compound 6j.

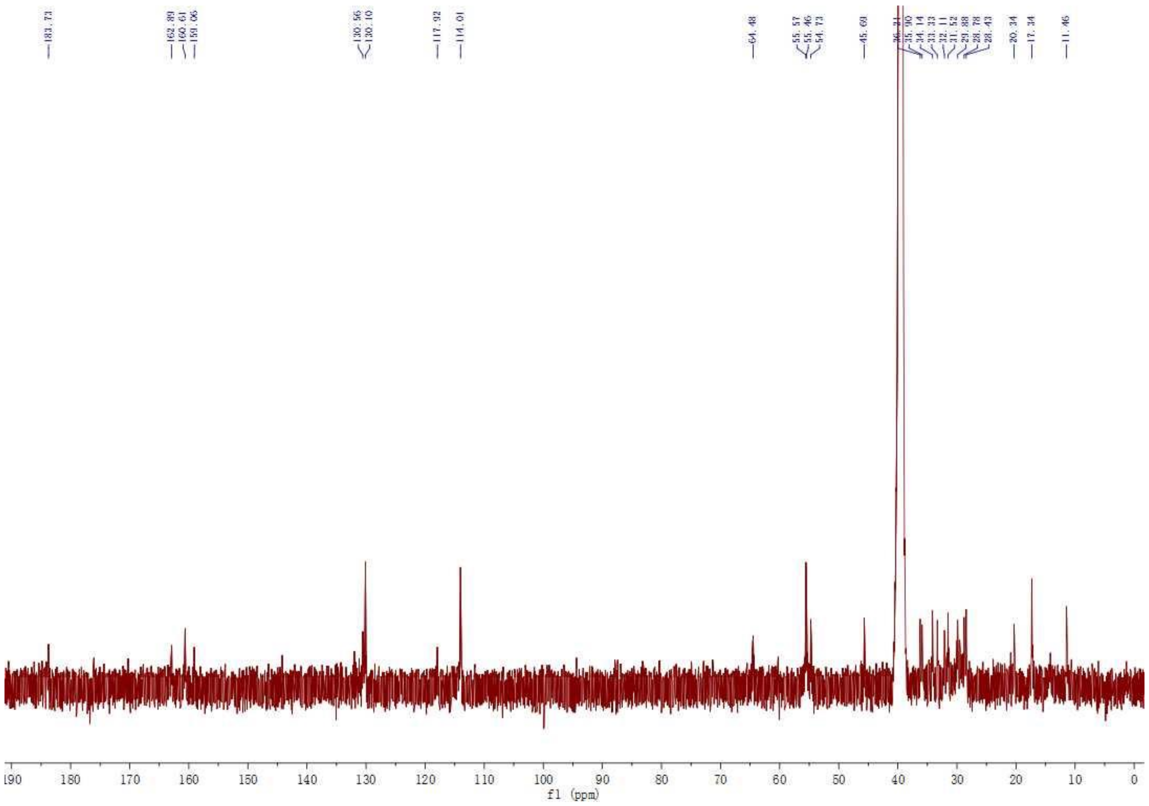

Figure S44: ^{13}C NMR spectrum for compound 6j.

Compd 6k

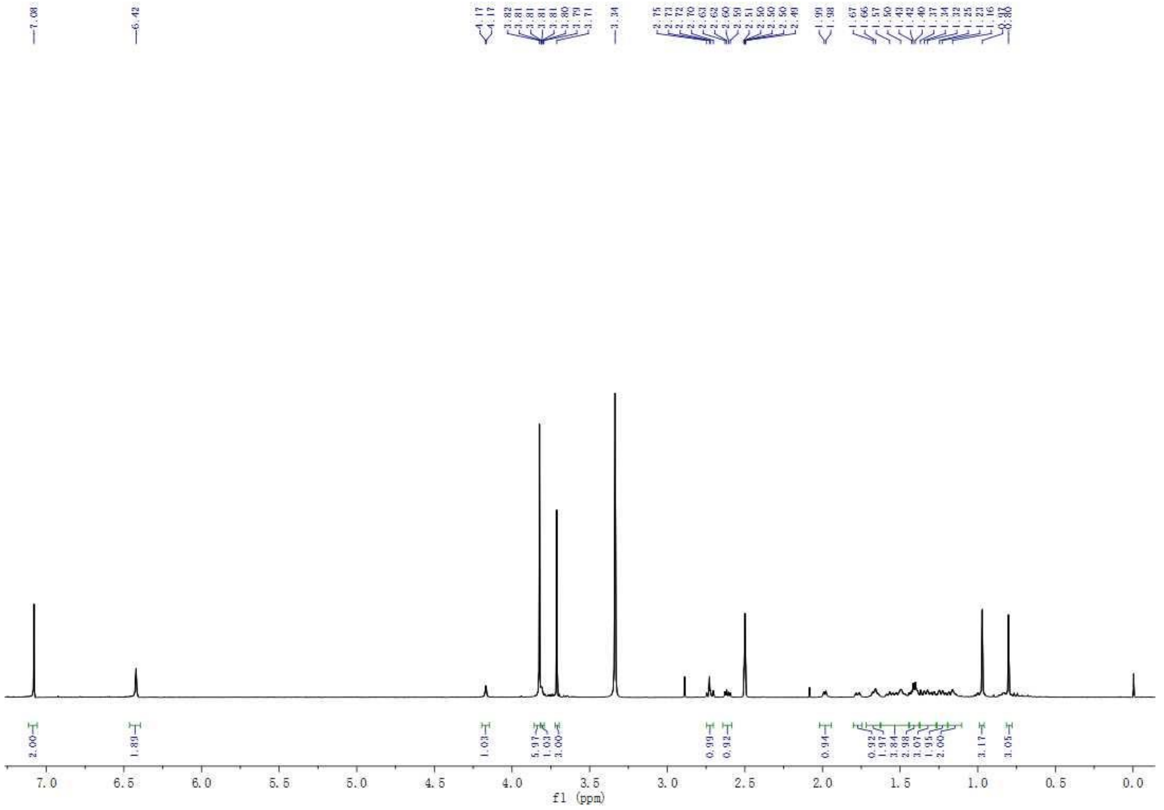

Figure S45: ^{1}H NMR spectrum for compound 6k.

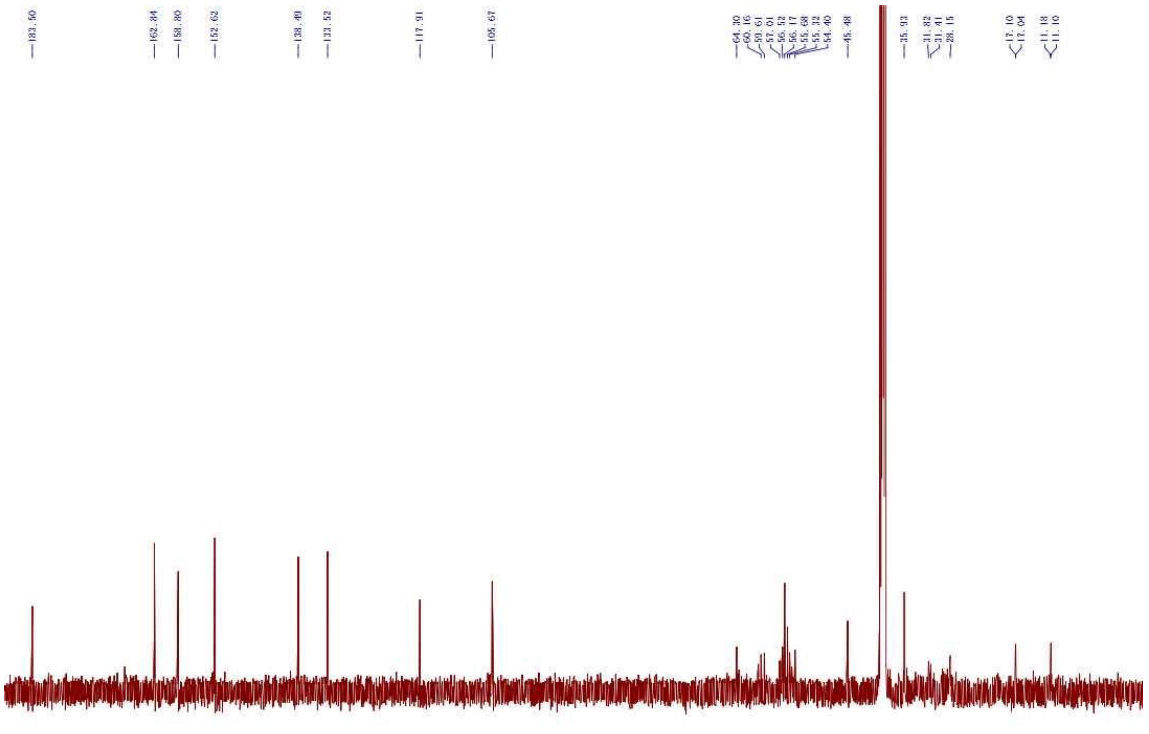

Figure S46: ^{13}C NMR spectrum for compound 6k.

Compd 6l

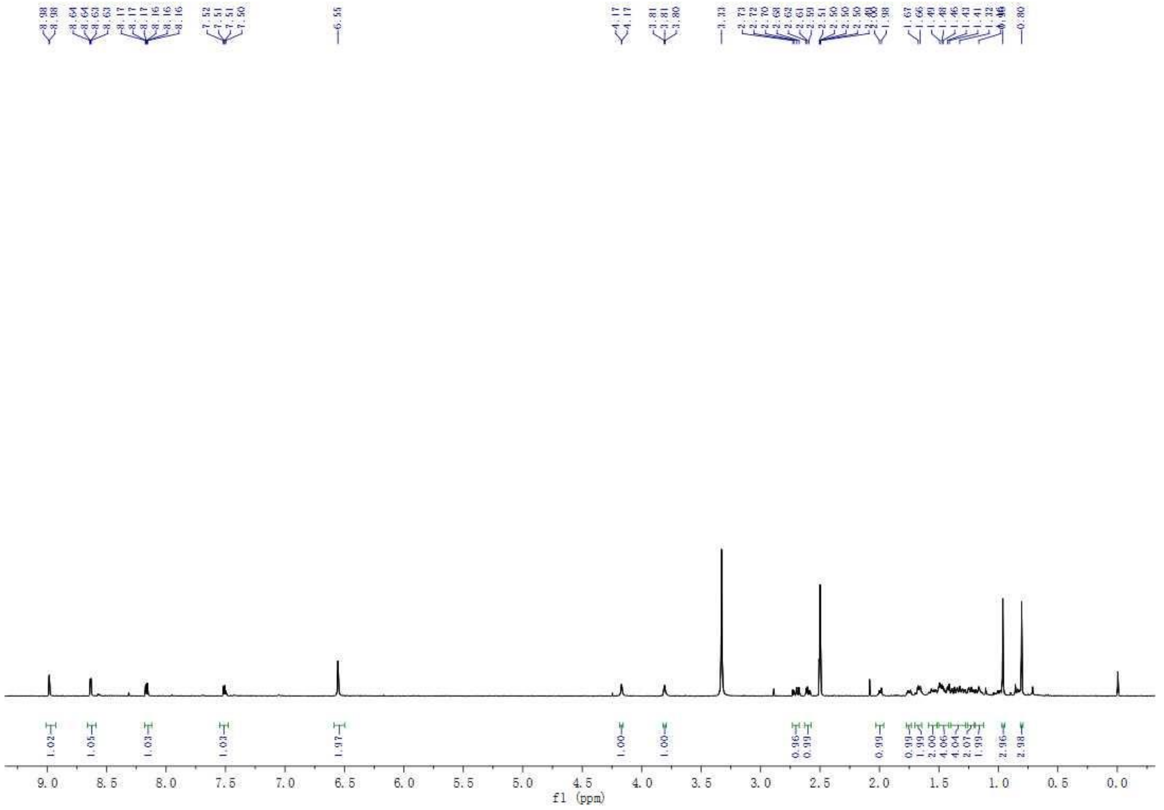

Figure S47: ^1H NMR spectrum for compound 6l.

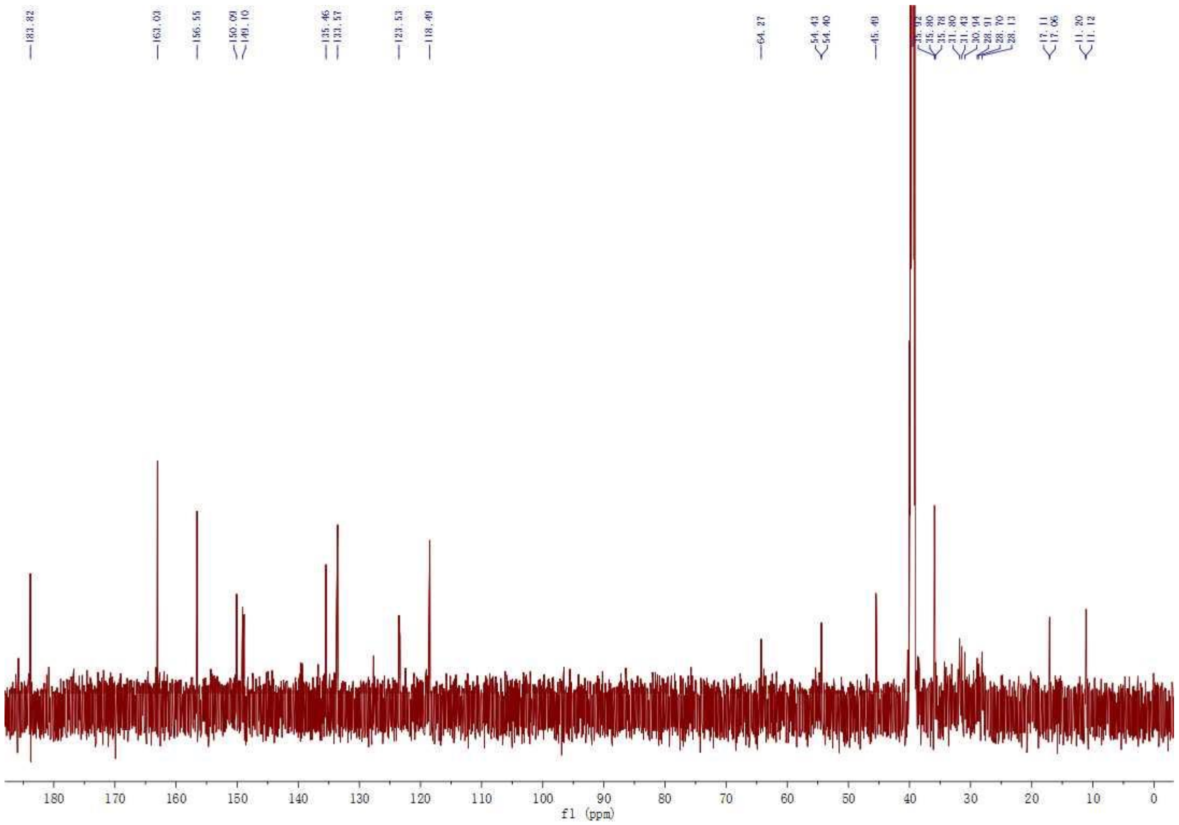

Figure S48: ^{13}C NMR spectrum for compound 6l.
